# Major Hemorrhage Risk Associated with Direct Oral Anticoagulants in Non-Valvular Atrial Fibrillation: A Systematic Review and Meta-Analysis

**DOI:** 10.31083/j.rcm2310334

**Published:** 2022-10-10

**Authors:** Paraschos Archontakis-Barakakis, Damianos G. Kokkinidis, Sanjana Nagraj, Vipul Gidwani, Theodoros Mavridis, George Ntaios

**Affiliations:** ^1^Northeast Internal Medicine Associates, LaGrange, IN 46845, USA; ^2^Section of Cardiovascular Medicine, Yale University School of Medicine, Yale New Haven Hospital, New Haven, CT 06510, USA; ^3^Department of Medicine, Albert Einstein College of Medicine/Jacobi Medical Center, Bronx, NY 10461, USA; ^4^1st Department of Neurology, Eginition Hospital, 11528 Athens, Greece; ^5^Department of Internal Medicine, University of Thessaly, 38221 Larissa, Greece

**Keywords:** non-valvular atrial fibrillation, major hemorrhage, direct oral anticoagulants, vitamin K antagonists

## Abstract

**Background:**

Real-world, observational studies have investigated the safety profile of Direct Oral Anticoagulants (DOACs) on Major Hemorrhage (MH) used for stroke prevention in Non-Valvular Atrial Fibrillation (NVAF). We performed a systematic review and meta-analysis to investigate the comparative safety of DOACs versus other DOACs and versus Vitamin K Antagonists (VKAs) adhering to PRISMA guidelines. We defined MH according to the International Society on Thrombosis and Haemostasis statement or as the composite outcome of intracranial, gastrointestinal, genitourinary, respiratory, cavitary and musculoskeletal bleeding in case of studies using International Statistical Classification of Diseases codes for patient selection.

**Methods:**

We systematically investigated two databases (Medline, Embase) until April of 2021, gathered observational studies and extracted hazard ratios (HRs) with 95% confidence intervals (CI) on our outcome of interest. Additional subgroup analyses according to DOAC dosing, prior diagnosis of chronic kidney disease, prior diagnosis of stroke, history of previous use of VKA, the users’ age, the users’ gender and study population geographic region were conducted. All analyses were performed with a random-effects model.

**Results:**

From this search, 55 studies were included and 76 comparisons were performed. The MH risk associated with Rivaroxaban use was higher than the risk with Dabigatran use (HR: 1.32, 95% CI: 1.21–1.45, $I^{2}$: 12.39%) but similar to VKA use (HR: 0.94, 95% CI: 0.87–1.02, $I^{2}$: 76.57%). The MH risk associated with Dabigatran use was lower than the risk with VKA use (HR: 0.75, 95% CI: 0.64–0.90, $I^{2}$: 87.57%). The MH risk associated with Apixaban use was lower than the risk with Dabigatran use (HR: 0.75, 95% CI: 0.64–0.88, $I^{2}$: 58.66%), with Rivaroxaban use (HR: 0.58, 95% CI: 0.50–0.68, $I^{2}$: 74.16%) and with VKA use (HR: 0.60, 95% CI: 0.55–0.65, $I^{2}$: 58.83%). Our aforementioned subgroup analyses revealed similar results.

**Conclusions:**

All in all, Apixaban was associated with a reduced MH risk compared to Dabigatran, Rivaroxaban and VKA. Dabigatran was associated with a reduced MH risk compared to both Rivaroxaban and VKA.

## 1. Introduction

Stroke is one of the most common and potentially debilitating medical conditions [1] and places a substantial financial burden on healthcare systems worldwide [2]. At least one third of strokes is caused by atrial fibrillation (AF) [3, 4]. AF is associated with higher severity strokes compared to other common etiologies, such as carotid disease, because of their larger volume and commonly multi-territorial nature [5, 6].

International guidelines and expert opinion agree on incorporating anticoagulation in regimens prescribed to patients with Non-Valvular AF (NVAF) for stroke prevention purposes [7, 8]. Direct Oral Anticoagulants (DOACs) and Vitamin K Antagonists (VKAs) are used to achieve this goal [9]. DOACs seem to be safer than VKAs in regard to the hemorrhagic risk associated with the use of anticoagulation [10, 11, 12, 13].

Except for these randomized, clinical trials (RCTs), observational studies have also demonstrated the effectiveness and safety of DOACs [14, 15, 16]. These studies have performed comparisons between DOACs and VKA, an investigation similar in nature to the RCTs’ methodology, and comparisons between DOAC agents. They have also focused on a variety of age groups, on specific comorbidities in addition to AF, on dosing regimens and have included population samples from different geographic locations (**Supplementary Material**).

We performed a systematic review and meta-analysis of observational (prospective and retrospective) studies to investigate the comparative risk of major hemorrhage (MH) between different DOAC agents and between DOACs and VKA in patients with NVAF. We defined MH according to the International Society on Thrombosis and Haemostasis (ISTH) statement or as the composite outcome of intracranial, gastrointestinal, genitourinary, respiratory, cavitary and musculoskeletal bleeding in case of studies using International Statistical Classification of Diseases and Related Health Problems (ICD) codes for patient selection [17].

## 2. Materials and Methods 

We adhered to the Preferred Reporting Items for Systematic Reviews and Meta-Analyses (PRISMA) guidelines to produce this study [18].

### 2.1 Study Selection

Two independent researchers (DKG, SN) systematically searched two large, online databases (Medline, Embase) until April of 2021. Consensus was reached via the intervention of a reviewer (PAB) if a disagreement between the initial researchers was identified. The search terms we used for our online investigation were (“novel oral anticoagulants” OR “direct oral anticoagulants” OR “non-vitamin K antagonist oral anticoagulants” OR NOAC OR DOAC OR dabigatran OR rivaroxaban OR apixaban OR warfarin OR coumadin OR “vitamin K antagonist”) AND (atrial fibrillation OR AF OR AFIB) AND (real-world OR “real world” OR observational OR cohort OR post-approval). As evident by our algorithm, Edoxaban was not included in our study. This decision of ours was based on our intention to make our results as generalizable to the worldwide population as possible. At the time of our search the use of this agent was lagging in Europe compared to other areas, research data from the specific geographic location was scarce and thus this agent was excluded from investigation. We also assessed the eligibility of studies used as references in observational studies and in literature reviews. Our inclusion criteria were: (i) retrospective or prospective observational studies, (ii) studies comparing at least one DOAC to another DOAC or studies comparing at least one DOAC to VKA, (iii) studies providing results in the form of Hazard Ratio (HR) with 95% Confidence Intervals (95% CI) on MH. Our exclusion criteria were (i) RCTs, (ii) studies investigating anticoagulation for valvular AF, (iii) studies investigating the effect of DOACs prescribed for another indication (e.g., venous thromboembolism).

### 2.2 Data Extraction and Outcomes

Two independent researchers (PAB and DGK) performed the data extraction using a pre-constructed form. Upon identification of a discrepancy, a reviewer (TM) was involved in order to reach consensus.

The single outcome of our investigation was MH. For each study, we assessed the authors’ definition of MH to verify appropriate alignment with the ISTH statement. When our source studies used databases to create their population sample, the complete alignment with the ISTH definition was deemed unrealistic and we focused on the provided ICD codes to ensure that their documented MH definition appropriately included bleeding in critical areas or organs.

HRs with 95% CIs comparing DOACs to other DOACs and DOACs to VKA were extracted. The specific pairwise comparisons of interest were Dabigatran to Rivaroxaban, Apixaban to Dabigatran and Apixaban to Rivaroxaban, Dabigatran to VKA, Rivaroxaban to VKA, Apixaban to VKA and finally DOACs (combination of Dabigatran, Rivaroxaban and Apixaban) to VKA. In addition, HRs on dosing regimens were extracted and separate categories were created. The “Low Dose” category was created to register results for the lower dose of a DOAC, e.g., 2.5 mg of apixaban two times a day, the “Normal Dose” category for the higher dose and the “Combined Doses” category for results on users of both doses or in the event of a study not providing a distinction. Except for data on our main analysis comparisons and dose specific comparisons, separate HRs were extracted for several subgroups. Our choice of subgroups was based on clinical criteria in order to assist providers prescribing anticoagulants around the world and on our intension to control potential bias and reduce the heterogeneity in our analyses. The subgroups that were formed were (i) patients with chronic kidney disease (CKD), (ii) patients who had already sustained a stroke (Post-stroke patients), (iii) patients previously prescribed VKA (Experienced users), (iv) users aged >65 years old, (v) users aged >75 years old, (vi) male users, (vii) female users, (viii) Asian users, (ix) American users and (x) European users.

### 2.3 Risk of Bias Assessment 

The risk of bias assessment was performed for each study with the Risk of Bias in Non-Randomized Studies of Interventions (ROBINS-I) tool by two, independent to each other researchers (DGK, VG) [19].

### 2.4 Statistical Analysis

Our main priority prior to any analysis was to ensure the removal of possible duplicate populations from each comparison. If such a possibility was appreciated (e.g., among studies using the same source/database, collecting data in the same time frame), we did not analyze the respective HRs together. On each such occasion, we chose to maintain the population sample that was best representative of the intended population or subgroup and to eliminate the other sample or samples. The mutually exclusive subgroups from a study (e.g., male and female patients) were included independently in each comparison, even alongside another group from the same study, to best avoid duplicate patients and to utilize the maximal possible and most representative population.

As the populations of the included studies varied significantly from a geographic location, gender distribution etc. perspective, we used by default a random effects model. Heterogeneity was quantified with calculation of the Higgins I-square ($I^{2}$) statistic, the Q value and *p*-value for Q. A cutoff of $I^{2}$>75% was used to indicate significant heterogeneity. We created forest plots to visually depict each comparison. We used both the Egger’s test and Funnel plots for risk of bias assessment. However, the latter method was used only when more than nine studies or study groups were included in the specific comparison. A *p* value of <0.05 was considered statistically significant. The statistical analysis was performed with R (version 4.2.1, R Foundation for Statistical Computing, Vienna, Austria) and RStudio (version 2022.07.1, RStudio Team, RStudio: Integrated Development for R. RStudio, PBC, Boston, MA, USA).

## 3. Results

**Fig. 1.**
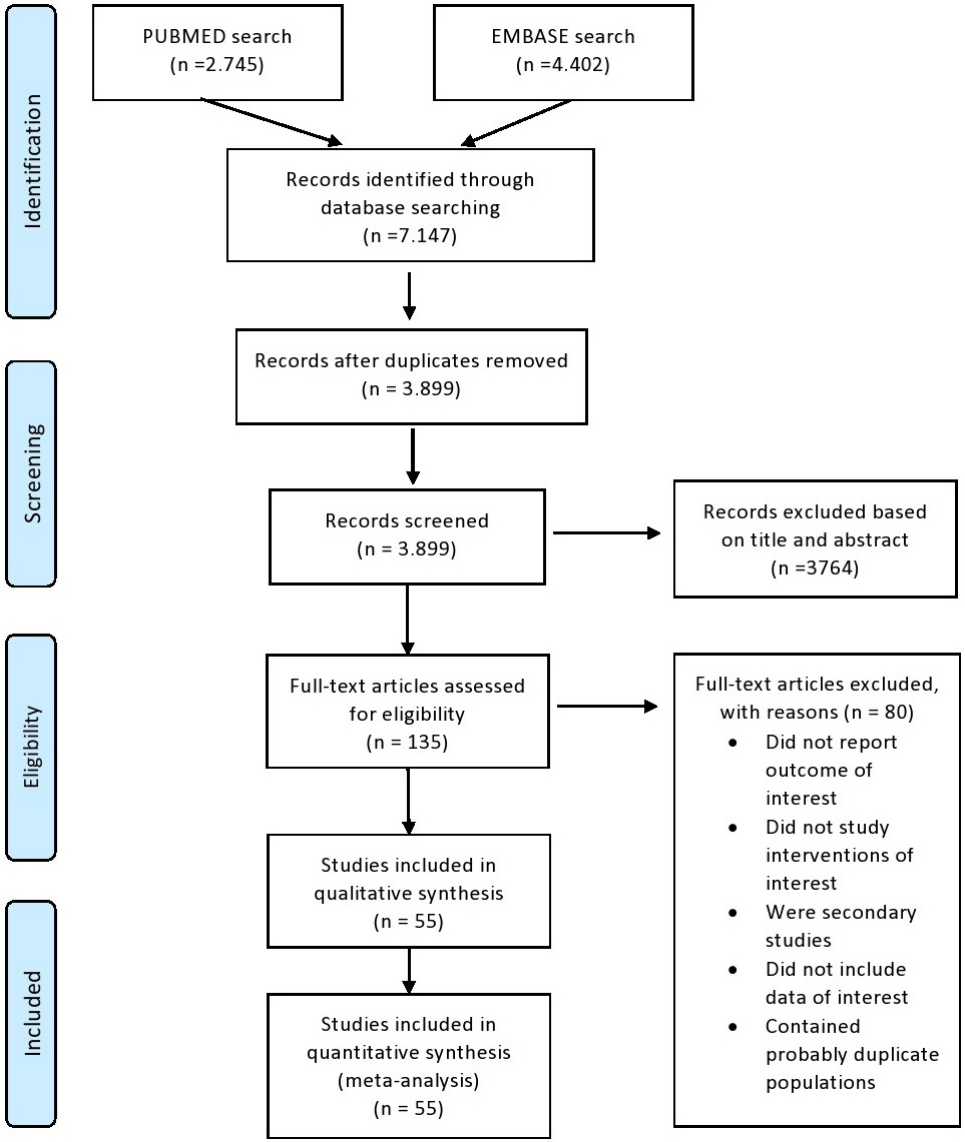
**PRISMA flowchart**.

Our search process concluded with 7.014 records screened, 135 full text articles assessed for eligibility and 55 studies finally deemed appropriate for inclusion (**Supplementary Material**). A PRISMA flowchart with this process is presented in Fig. 1.

The total sum of the populations from all the included studies exceeded 2,179,000 patients. The methodologic characteristics of each study as well as the baseline characteristics of their populations are presented in **Supplementary Table 1**.

As our study’s comparisons were numerous, two tables, one for the DOAC to DOAC comparisons (Table 1) and one for the DOAC to VKA comparisons (Table 2), were created to cumulatively present our results. This section is mainly dedicated to the presentation of statistically significant results.

**Table 1.** **Presentation of DOAC versus DOAC comparisons**.

MH:	Rivaroxaban (Combined) vs. Dabigatran	Rivaroxaban (Low Dose) vs. Dabigatran	Rivaroxaban (Normal Dose) vs. Dabigatran	Apixaban (Combined) vs. Dabigatran	Apixaban (Low Dose) vs. Dabigatran	Apixaban (Normal Dose) vs. Dabigatran	Apixaban (Combined) vs. Rivaroxaban	Apixaban (Low Dose) vs. Rivaroxaban	Apixaban (Normal Dose) vs. Rivaroxaban
Main Analysis	5; 1.32 (1.21–1.45); 12.39%		3; 1.33 (1.20–1.48); 0.00%	8; 0.75 (0.64–0.88); 58.66%		3; 0.82 (0.66–1.03); 54.97%	8; 0.58 (0.50–0.68); 74.16%		3; 0.60 (0.50–0.71); 71.93%
Patients with CKD	3; 1.20 (0.87–1.67); 77.80%			3; 0.73 (0.58–0.91); 0.00%			3; 0.63 (0.45–0.89); 77.46%		
Experienced Users									
Post-Stroke Patients									
Users Aged >65 years old	7; 1.38 (1.28–1.48); 0.00%			7; 0.82 (0.73–0.91); 29.89%			7; 0.61 (0.54–0.69); 76.25%		
Users Aged >75 years old	4; 1.36 (1.25–1.48); 0.00%			4; 0.78 (0.69–0.88); 18.10%			4; 0.59 (0.50–0.70); 81.77%		
Male Users				3; 0.91 (0.79–1.04); 36.28%			3; 0.65 (0.54–0.80); 78.37%		
Female Users				3; 0.76 (0.68–0.85); 0.00%			3; 0.64 (0.50–0.83); 84.70%		
Asian Users									
American Users	3; 1.33 (1.14–1.56); 48.00%			6; 0.67 (0.57–0.80); 26.13%			6; 0.53 (0.46–0.61); 41.70%		
European Users									

In Each Cell: Number of Studies included in the Comparison; Hazard Ratio (95% Confidence Interval); $I^{2}$.

**Table 2.** **Presentation of DOAC versus VKA comparisons**.

MH:	Dabigatran (Combined) vs. VKA	Dabigatran (Low Dose) vs. VKA	Dabigatran (Normal Dose) vs. VKA	Rivaroxaban (Combined) vs. VKA	Rivaroxaban (Low Dose) vs. VKA	Rivaroxaban (Normal Dose) vs. VKA	Apixaban (Combined) vs. VKA	Apixaban (Low Dose) vs. VKA	Apixaban (Normal Dose) vs. VKA	DOACs (Combined) vs. VKA
Main Analysis	16; 0.75 (0.64–0.90); 87.57%	9; 0.83 (0.76–0.91); 22.92%	10; 0.71 (0.61–0.81); 66.91%	15; 0.94 (0.87–1.02); 76.57%	9; 0.96 (0.86–1.09); 73.39%	11; 0.99 (0.90–1.08); 56.92%	18; 0.60 (0.55–0.65); 58.83%	8; 0.65 (0.57–0.74); 64.05%	8; 0.58 (0.53–0.64); 36.87%	12; 0.89 (0.69–1.15); 94.05%
Patients with CKD	3; 0.74 (0.65–0.84); 0.00%			5; 0.94 (0.74–1.20); 60.99%			4; 0.60 (0.50–0.72); 51.56%			
Experienced Users										
Post-Stroke Patients	3; 0.76 (0.59–0.96); 32.89%			3; 0.96 (0.73–1.27); 64.37%			4; 0.72 (0.59–0.88); 46.41%			3; 0.90 (0.75–1.06); 0.00%
Users Aged >65 years old	8; 0.78 (0.62–0.99); 91.36%		3; 0.75 (0.55–1.01); 85.62%	6; 1.02 (0.91–1.15); 83.75%	3; 0.88 (0.57–1.36); 92.69%	3; 1.09 (0.93–1.27); 59.26%	7; 0.60 (0.54–0.67); 51.05%			5; 0.86 (0.80–0.92); 13.82%
Users Aged >75 years old	4; 0.79 (0.70–0.90); 51.08%			4; 1.05 (0.92–1.21); 81.82%	3; 0.88 (0.57–1.36); 92.69%		5; 0.60 (0.52–0.70); 66.90%			3; 0.87 (0.82–0.93); 0.00%
Male Users							3; 0.71 (0.51–0.99); 91.56%			
Female Users							3; 0.64 (0.60–0.69); 0.00%			
Asian Users	6; 0.62 (0.55–0.70); 0.00%			4; 0.70 (0.54–0.90); 74.44%	3; 0.67 (0.40–1.13); 86.24%	4; 0.73 (0.53–1.00); 55.89%	4; 0.60 (0.45–0.79); 78.03%			7; 0.83 (0.79–0.87); 0.00%
American Users	6; 0.89 (0.66–1.20); 94.29%		4; 0.81 (0.65–0.99); 79.34%	8; 1.01 (0.95–1.08); 38.86%		4; 1.06 (0.93–1.22); 54.31%	7; 0.58 (0.52–0.64); 47.05%	3; 0.61 (0.52–0.72); 46.58%	3; 0.59 (0.55–0.62); 0.00%	
European Users	4; 0.70 (0.57–0.87); 55.99%	5; 0.86 (0.78–0.94); 0.00%	4; 0.60 (0.51–0.71); 20.86%	3; 1.01 (0.89–1.15); 55.48%	4; 1.08 (0.95–1.23); 49.82%	3; 1.02 (0.94–1.11); 0.00%	6; 0.64 (0.55–0.75); 52.51%	3; 0.72 (0.64–0.82); 0.00%	3; 0.57 (0.49–0.67); 25.33%	4; 0.91 (0.48–1.74); 97.01%

In Each Cell: Number of Studies included in the Comparison; Hazard Ratio (95% Confidence Interval); $I^{2}$.

### 3.1 Rivaroxaban versus Dabigatran

**Fig. 2.**
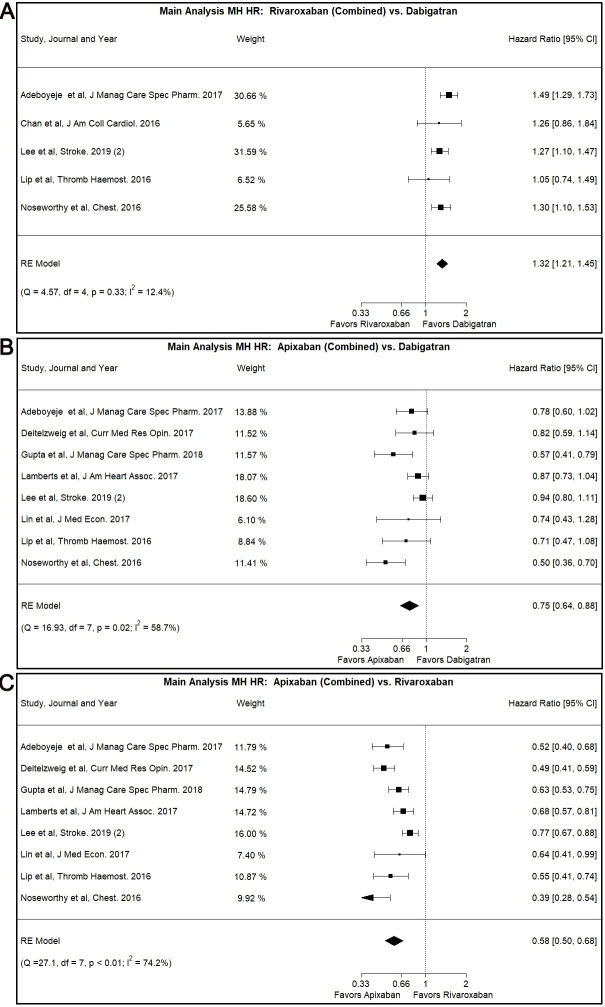
**Main Analysis Major Hemorrhage Risk, DOAC versus DOAC comparisons**. (A) Comparison between Rivaroxaban (Combined) and Dabigatran. (B) Comparison between Apixaban (Combined) and Dabigatran. (C) Comparison between Apixaban (Combined) and Rivaroxaban.

In our main analysis, Rivaroxaban was associated with higher risk for MH compared to Dabigatran (Combined: HR: 1.32, 95% CI: 1.21–1.45, $I^{2}$: 12.39%) (Fig. 2A). The same result was identified for the Normal Dose (HR: 1.33, 95% CI: 1.20–1.48, $I^{2}$: 0.00%), patients with CKD subgroup (Combined: HR: 1.20, 95% CI: 0.87–1.67, $I^{2}$: 77.80%), users aged >65 years old subgroup (Combined: HR: 1.38, 95% CI: 1.28–1.48, $I^{2}$: 0.00%), users aged >75 years old subgroup (Combined: HR: 1.36, 95% CI: 1.25–1.48, $I^{2}$: 0.00%), and American users subgroup (Combined: HR: 1.33, 95% CI: 1.14–1.56, $I^{2}$: 48.00%) analyses. (**Supplementary Fig. 1A–E**).

### 3.2 Apixaban versus Dabigatran

Our main analysis showed lower MH risk associated with the use of Apixaban as opposed to use of Dabigatran (Combined: HR: 0.75, 95% CI: 0.64–0.88, $I^{2}$: 58.66%) (Fig. 2B). The patients with CKD subgroup (Combined: HR: 0.73, 95% CI: 0.58–0.91, $I^{2}$: 0.00%), users aged >65 years old subgroup (Combined: HR: 0.82, 95% CI: 0.73–0.91, $I^{2}$: 29.89%), users aged >75 years old subgroup (Combined: HR: 0.78, 95% CI: 0.69–0.88, $I^{2}$: 18.10%), female users subgroup (Combined: HR: 0.76, 95% CI: 0.68–0.85, $I^{2}$: 0.00%) and American users subgroup (Combined: HR: 0.67, 95% CI: 0.57–0.80, $I^{2}$: 26.13%) analyses produced similar results (**Supplementary Fig. 2A–G**).

The Egger’s test was positive (*p *< 0.05) for the Combined category of the main analysis.

### 3.3 Apixaban versus Rivaroxaban

The use of Apixaban was shown to have decreased MH risk compared to Rivaroxaban use in our main analysis (Combined: HR: 0.58, 95% CI: 0.50–0.68, $I^{2}$: 74.16%/Normal Dose: HR: 0.60, 95% CI: 0.50–0.71, $I^{2}$: 71.93%) (Fig. 2C, **Supplementary Fig. 3A**).

This result was also demonstrated in the patients with CKD subgroup (Combined: HR: 0.63, 95% CI: 0.45–0.89, $I^{2}$: 77.46%), users aged >65 years old subgroup (Combined: HR: 0.61, 95% CI: 0.54–0.69, $I^{2}$: 76.25%), users aged >75 years old subgroup (Combined: HR: 0.59, 95% CI: 0.50–0.70, $I^{2}$: 81.77%), male users subgroup (Combined: HR: 0.65, 95% CI: 0.54–0.80, $I^{2}$: 78.37%), female users subgroup (Combined: HR: 0.64, 95% CI: 0.50–0.83, $I^{2}$: 84.70%) and American users subgroup (Combined: HR: 0.53, 95% CI: 0.46–0.61, $I^{2}$: 41.70%) analyses (**Supplementary Fig. 3B–G**).

### 3.4 Dabigatran versus VKA

**Fig. 3.**
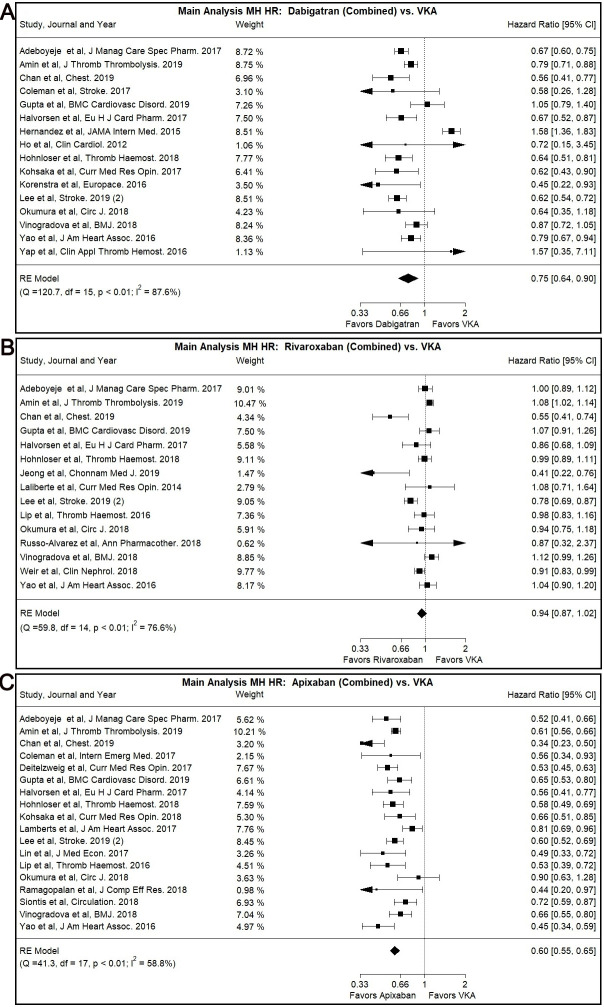
**Main Analysis Major Hemorrhage Risk, DOAC versus VKA comparisons**. (A) Comparison between Rivaroxaban (Combined) and VKA. (B) Comparison between Apixaban (Combined) and VKA. (C) Comparison between Apixaban (Combined) and VKA.

Dabigatran use was associated with lower MH risk compared to VKA use in our main analysis (Combined: HR: 0.75, 95% CI: 0.64–0.90, $I^{2}$: 87.57%/Low dose: HR: 0.83, 95% CI: 0.76–0.91, $I^{2}$: 22.92%/Normal Dose: HR: 0.71, 95% CI: 0.61–0.81, $I^{2}$: 66.91%) (Fig. 3A, **Supplementary Fig. 4A,B**).

Most subgroup analyses, specifically patients with CKD subgroup (Combined: HR: 0.74, 95% CI: 0.65–0.84, $I^{2}$: 0.00%), Post-Stroke patients subgroup (Combined: HR: 0.76, 95% CI: 0.59–0.96, $I^{2}$: 32.89%), users aged >65 years old subgroup (Combined: HR: 0.78, 95% CI: 0.62–0.99, $I^{2}$: 91.36%), users aged >75 years old subgroup (Combined: HR: 0.79, 95% CI: 0.70–0.90, $I^{2}$: 51.08%), Asian users subgroup (Combined: HR: 0.62, 95% CI: 0.55–0.70, $I^{2}$: 0.00%), American users subgroup (Normal Dose: HR: 0.81, 95% CI: 0.65–0.99, $I^{2}$: 79.34%), European users subgroup (Combined: HR: 0.70, 95% CI: 0.57–0.87, $I^{2}$: 55.99%/Low Dose: HR: 0.86, 95% CI: 0.78–0.94, $I^{2}$: 0.00%/Normal Dose: HR: 0.60, 95% CI: 0.51–0.71, $I^{2}$: 20.86%), resulted in similar findings (**Supplementary Fig. 4C–M**).

The Egger’s test was positive (*p *< 0.05) for the Combined and Normal Dose categories of the users aged >65 years old subgroup analyses.

### 3.5 Rivaroxaban versus VKA

Rivaroxaban was not associated with significantly different MH risk compared to VKA in our main or subgroup analyses with the notable exception of Asian users subgroup (Combined: HR: 0.70, 95% CI: 0.54–0.90, $I^{2}$: 74.44%) (Fig. 3B, **Supplementary Fig. 5A–Q**).

The Egger’s test was positive (*p *< 0.05) for all categories of the main analysis, for the users aged >65 years old subgroup analysis and for the users aged >75 years old subgroup analysis.

### 3.6 Apixaban versus VKA

The use of Apixaban was shown to have significantly lower MH risk compared to VKA use (Combined: HR: 0.60, 95% CI: 0.55–0.65, $I^{2}$: 58.83%/Low Dose: HR: 0.65, 95% CI: 0.57–0.74, $I^{2}$: 64.05%/Normal Dose: HR: 0.58, 95% CI: 0.53–0.64, 36.87%) with our main analysis (Fig. 3C, **Supplementary Fig. 6A,B**).

A similar trend was observed with many of our subgroup analyses, namely the patients with CKD subgroup (Combined: HR: 0.60, 95% CI: 0.50–0.72, $I^{2}$: 51.56%), the Post-Stroke patients subgroup (Combined: HR: 0.72, 95% CI: 0.59–0.88, $I^{2}$: 46.41%), the users aged >65 years old subgroup (Combined: HR: 0.60, 95% CI: 0.54–0.67, $I^{2}$: 51.05%), the users aged >75 years old subgroup (Combined: HR: 0.60, 95% CI: 0.52–0.70, $I^{2}$: 66.90%), the male users subgroup (Combined: HR: 0.71, 95% CI: 0.51–0.99, $I^{2}$: 91.56%), the female users subgroup (Combined: HR: 0.64, 95% CI: 0.60–0.69, $I^{2}$: 0.00%), the Asian users subgroup (Combined: HR: 0.60, 95% CI: 0.45–0.79, $I^{2}$: 78.03%), the American users subgroup (Combined, HR: 0.58. 95% CI: 0.52–0.64, $I^{2}$: 47.05%/Low Dose: HR: 0.61, 95% CI: 0.52–0.72, $I^{2}$: 46.58%/Normal Dose: HR: 0.59, 95% CI: 0.55–0.62, $I^{2}$: 0.00%) and the European users subgroup (Combined: HR: 0.64, 95% CI: 0.55–0.75, $I^{2}$: 52.51%/Low Dose: HR: 0.72, 95% CI: 0.64–0.82, $I^{2}$: 0.00%/Normal Dose: HR: 0.57, 95% CI: 0.49–0.67, $I^{2}$: 25.33%) (**Supplementary Fig. 6C–O**).

### 3.7 DOACs versus VKA

No statistically different risk of MH was identified by the comparison of DOACs use (combination of Dabigatran, Rivaroxaban and Apixaban) versus VKA.

Lower risk of MH was observed both for the users aged >65 years old subgroup (Combined: HR: 0.86, 95% CI: 0.80–0.92, $I^{2}$: 13.82%), users aged >75 years old (Combined: HR: 0.87, 95% CI: 0.82–0.93, $I^{2}$: 0.00%) and Asian users subgroup (Combined: HR: 0.83, 95% CI: 0.79–0.87, $I^{2}$: 0.00%) analyses (**Supplementary Fig. 7A–F**).

### 3.8 Publication and Other Types of Bias Assessment

The Egger’s test was negative, suggestive of possible absence of publication bias, for most of the comparisons with the entire list of exceptions presented in **Supplementary Table 2**.

All our comparisons with 10 or more included studies resulted in additional Funnel Plot creation. All of them are presented in **Supplementary Fig. 8A–F**.

Because of the nature of our included studies, all of them were deemed to reach moderate or higher level of bias. This assessment is further expanded in our Discussion section.

## 4. Discussion

Our study was a systematic review and meta-analysis of 55 “real-world” studies comparing the MH risk associated with the use of DOACs versus other DOACs and VKA.

We concluded that (i) Rivaroxaban was associated with higher MH risk compared to Dabigatran (ii) Apixaban was associated with lower MH risk compared to Dabigatran (iii) Apixaban was associated with lower MH risk compared to Rivaroxaban (iv) Dabigatran was associated with a lower MH risk compared to VKA (vi) Rivaroxaban was not associated with a significantly different MH risk compared to VKA (vii) Apixaban was associated with a lower MH risk compared to VKA (iv) DOACs as a whole were not associated with a significantly different MH risk compared to VKA.

While the superiority of DOACS compared to VKA regarding several outcomes, including MH, is established, the main research focus is pivoting towards the comparison between different DOACs. As no RCT has performed a head-to-head comparison between DOACs, both observational studies and meta-analyses of observational studies provide valuable insight on this specific question. Our study demonstrates that Apixaban is associated with a lower risk for MH compared to both Rivaroxaban and Dabigatran. Dabigatran was also associated with a lower risk for MH compared to Rivaroxaban. According to our previously noted calculations, no significant source of bias was deemed to affect our results. At the same time, it needs to be underlined that no “one-size-fits-all” solution exists and that clinicians would be advised to individually assess each patient’s profile and tailor the anticoagulation to best fit their needs. For example, the use of dabigatran is associated with decreased risk of intracranial hemorrhage and this agent might be a better choice for a patient at increased risk of such a complication [20].

Placing our study’s conclusions against conclusions reached by large-scale RCTs, we identified certain similarities. The first RCT on this subject (the Randomized Evaluation of Long-Term Anticoagulation Therapy, RE-LY) investigated the efficacy and safety of Dabigatran versus VKA. The researchers identified a lower rate of MH with the use of the Low Dose of Dabigatran compared to VKA but were unable to prove a statistically significant result for the Normal Dose [10]. Our results agree with the first of those findings and reinforce the statistical significance of the latter. The Rivaroxaban Once Daily Oral Direct Factor Xa Inhibition Compared with Vitamin K Antagonism for Prevention of Stroke and Embolism Trial in Atrial Fibrillation trial introduced rivaroxaban as an anticoagulant for the management of NVAF. There was no statistically significant difference on MH risk appreciated between the rivaroxaban and the warfarin groups, a conclusion corroborated by our results [11]. Finally, the last of our investigated DOAC agents, apixaban, was introduced by the Apixaban for Reduction in Stroke and Other Thromboembolic Events in Atrial Fibrillation (ARISTOTLE) trial. In this trial, it was demonstrated that patients with NVAF anticoagulated with Apixaban had a lower MH risk compared to those anticoagulated with warfarin [12]. Our results align adequately with these findings.

Our results are also in agreement with previously conducted observational studies (**Supplementary Material**) and meta-analyses. For example, it was shown in a recent meta-analysis that Dabigatran use had decreased risk of MH compared to VKA use, although this result did not reach statistical significance, that Rivaroxaban use had similar risk compared to VKA use and that Apixaban use had lower risk compared to VKA use [21]. Prior to our study, the efficacy and safety of DAOCs among the Asian population was investigated by Li *et al*. [22]. All in all, it seems that the efficacy and safety profile of DOACs demonstrated by RCTs is rather well supported by the effectiveness and safety profile demonstrated by observational studies and meta-analyses.

### 4.1 Strengths and Limitations 

There are several strengths appreciated in our study. First, we adhered strictly to systematic review and meta-analysis methodology. Second, we implemented a narrow focus on MH and attempted to provide answers to a specific and clinically relevant question. Third, all our investigations and analyses were performed according to our initial plan thus avoiding the addition of bias to our study. Fourth, we were able to search, collect, screen and analyze a large number of studies and thus a substantial patient population. All in all, we were able to perform the largest to-date real-world data meta-analysis on this topic.

Despite these strengths, we would also like to acknowledge certain weaknesses of our study. First, since our primary data is derived from observational studies, our study is restricted by certain limitations linked to this type of research. The most pertinent of them would be the possible presence of unmeasured and uncontrolled confounding factors especially considering that most of our source material studies formed their respective populations from databases using International Statistical Classification of Diseases and Related Health Problems (ICD) codes for patient selection. Such confounding factors would possibly persist the transfer to our study and translate to different types of bias, among which selection bias and bias by indication would be the most important. For example, we had little data available to determine the percentage of patients using the on-label dose of each DOAC in each study. At the same time, it is known that the off-label use of DOACs varies widely among different countries and is identified as a limiting factor of all observational studies on this subject [23]. As such, we used our dosing categories as described above without being able to verify the appropriate dosing in each category. Nonetheless, our goal of presenting “real-world” results on DOAC use led us to acknowledge and accept this possibility of bias. Second, a few of our analyses were primarily driven by a small number of studies. This phenomenon occurred either because of the high weight attributed to them or because of a lack of a higher number of studies to be included in that specific comparison. Third, we observed significant heterogeneity among studies while reporting data on MH. In order to mitigate this effect, both a random effect model was implemented and analyses on several and clinically relevant subgroups were performed. Fourth, we were unable to collect data on the DOACs users’ functional status and most importantly their frailty diagnosis because of the paucity of such information in our source material. Finally, we did not include comparisons of Edoxaban as explained above.

## 5. Conclusions 

In conclusion, this is the largest systematic review and meta-analysis on the comparison of DOACs versus other DOACs and versus VKA on MH risk. Apixaban was associated with a reduced MH risk compared to Dabigatran, Rivaroxaban and VKA. Also, Dabigatran was associated with a reduced MH risk compared to both Rivaroxaban and VKA.

## Data Availability

Our data is derived from public domain resources. All data source material that supports the findings of this study are available on Medline and Embrace.

## Supplementary Material

Supplementary material associated with this article can be found, in the online version, at https://doi.org/10.31083/j.rcm2310334.
