# Supplementary material for: Major Hemorrhage Risk Associated with Direct Oral Anticoagulants in Non-Valvular Atrial Fibrillation: A Systematic Review and Meta-Analysis
Source: Rev Cardiovasc Med. 2022 Oct 10;23(10):334. doi: 10.31083/j.rcm2310334 (PMC11267317; doi:10.31083/j.rcm2310334)
Supplement: Supplementary file 1 [file 2153-8174-23-10-334-s1.zip › eMaterial - 2.2.docx]

**Major Hemorrhage risk associated with Direct Oral Anticoagulants in Non-Valvular Atrial Fibrillation: A Systematic Review and Meta-analysis**

**Supplementary Material**

Contents

[**Supplementary Table 1** 2](#_Toc113436310)

[**Supplementary Figure 1** 15](#_Toc113436311)

[**Supplementary Figure 2** 17](#_Toc113436312)

[**Supplementary Figure 3** 19](#_Toc113436313)

[**Supplementary Figure 4** 21](#_Toc113436314)

[**Supplementary Figure 5** 25](#_Toc113436315)

[**Supplementary Figure 6** 30](#_Toc113436316)

[**Supplementary Figure 7** 34](#_Toc113436317)

[**Supplementary Figure 8** 36](#_Toc113436318)

[**Supplementary Table 2** 38](#_Toc113436319)

[**Supplementary References** 39](#_Toc113436320)

**Supplementary Table 1**

| **Study** | **Design** | **Setting/Data source** | **Country/ Region** | **Study Enrollement Period** | **No. VKA** | **No. Dabigatran (Low Dose)** | **No. Dabigatran (High Dose)** | **No. Rivaroxaban (High Dose)** | **No. Rivaroxaban (Low Dose)** | **No. Apixaban (High Dose)** | **No. Apixaban (Low Dose)** |
| --- | --- | --- | --- | --- | --- | --- | --- | --- | --- | --- | --- |
| Adeboyeje et al, J Manag Care Spec Pharm. 2017 | Retrospective cohort | HealthCore Integrated Research Environment (HIRE) | US | November 1, 2009 to January 31, 2016 | 23.431 | 8.539 | | 8.398 | | 3.689 | |
| Amin et al, J. Thromb. Thrombolysis 2019 | Retrospective cohort | Centers for Medicare & Medicaid Services (CMS) | US | January 1, 2012 to December 31, 2015 | 81.410 | 18.162 | | 60.133 | | 38.466 | |
| Arihiro et al, Int. J. Stroke 2016 | Prospective cohort | 18 stroke centers | Japan | September, 2011 to March, 2014 | 662 | 205 | | 245 | | 25 | |
| Chan et al, Chest 2019 | Retrospective cohort | Taiwan's National Health Insurance Research Database | Taiwan | June 1, 2012 to December 31, 2017 | 19.761 | 19.821 | 2.550 | 1.914 | 31.108 | 3.593 | 6.359 |
| Chan et al, J Am Coll Cardiol. 2016 | Retrospective cohort | Taiwan's National Health Insurance Research Database | Taiwan | February 1, 2013 to December 31, 2013 | 5.251 | 5.301 | 620 | 491 | 3.425 | NR/NA | NR/NA |
| Chan et al, Stroke. 2016 | Retrospective cohort | Taiwan's National Health Insurance Research Database | Taiwan | January 1, 1996 to December 31, 2013 | 9.913 | 9.940 | | NR/NA | NR/NA | NR/NA | NR/NA |
| Chao et al, Chest 2020 | Retrospective cohort | Taiwan's National Health Insurance Research Database | Taiwan | January 1, 2012 to December 31, 2015 | 18.537 | 45.632 | | | | | |
| Cho et al, Stroke. 2018 | Retrospective cohort | Korean National Health Insurance Service Database | Korea | July 1, 2015, and December 31, 2016 | 10.409 | 12.593 | | 21.000 | | 12.502 | |
| Coleman et al, Am J Med. 2019 | Retrospective cohort | Truven MarketScan Database | US | January, 2012 to December, 2017 | 4.848 | NR/NA | NR/NA | 1.896 | | NR/NA | NR/NA |

Supplementary Table 1 (Continued)

| **Study** | **Age VKA (mean, SD)** | **Age Low Dose Dab. (mean, SD)** | **Age High Dose Dab. (mean, SD)** | **Age High Dose Riv. (mean, SD)** | **Age Low Dose Riv. (mean, SD)** | **Age High Dose Api. (mean, SD)** | **Age Low Dose Api. (mean, SD)** | **Male (%) VKA** | **Male (%) Low Dose Dab.** | **Male (%) High Dose Dab.** | **Male (%) High Dose Riv.** | **Male (%) Low Dose Riv.** | **Male (%) High Dose Api.** | **Male (%) Low Dose Api.** |
| --- | --- | --- | --- | --- | --- | --- | --- | --- | --- | --- | --- | --- | --- | --- |
| Adeboyeje et al, J Manag Care Spec Pharm. 2017 | 70 (12.2) | 70 (12.3) | | 70 (12.3) | | 70 (12.6) | | 59,1 | 58,9 | | 58,7 | | 59,5 | |
| Amin et al, J. Thromb. Thrombolysis 2019 | 78.9 (7.5) | 77 (7) | | 77.6 (7.3) | | 72.79 (10.98) | | 50,4 | 51,4 | | 49,7 | | 48,3 | |
| Arihiro et al, Int. J. Stroke 2016 | 79.3 (9.7) | 74.4 (9.2) | | | | | | 51,2 | 64,4 | | | | | |
| Chan et al, Chest 2019 | 74.6 (10.7) | 74.7 (10.7) | | 74.7 (10.7) | | 74.8 (10.5) | | 56,7 | 57,4 | | 57,5 | | 57,6 | |
| Chan et al, J Am Coll Cardiol. 2016 | 71 (12) | 75 (9) | | 76 (9) | | NR/NA | NR/NA | 56 | 58 | | 54 | | NR/NA | NR/NA |
| Chan et al, Stroke. 2016 | 76 (10) | 75 (10) | | NR/NA | NR/NA | NR/NA | NR/NA | 58 | 58 | | NR/NA | NR/NA | NR/NA | NR/NA |
| Chao et al, Chest 2020 | 78.26 (7.86) | 78.65(7.44) | | | | | | 50,7 | 54 | | | | | |
| Cho et al, Stroke. 2018 | 70.8 (11.0) | 72.9 (8.9) | | 73.8 (8.8) | | 74.3 (8.9) | | 54 | 53,6 | | 50,9 | | 47,7 | |
| Coleman et al, Am J Med. 2019 | 72 [63-80] | NR/NA | NR/NA | 72 [63-80] | | NR/NA | NR/NA | 61,6 | NR/NA | NR/NA | 58,4 | | NR/NA | NR/NA |

Supplementary Table 1 (Continued)

| **Study** | **Design** | **Setting/Data source** | **Country/ Region** | **Study Enrollement Period** | **No. VKA** | **No. Dabigatran (Low Dose)** | **No. Dabigatran (High Dose)** | **No. Rivaroxaban (High Dose)** | **No. Rivaroxaban (Low Dose)** | **No. Apixaban (High Dose)** | **No. Apixaban (Low Dose)** |
| --- | --- | --- | --- | --- | --- | --- | --- | --- | --- | --- | --- |
| Coleman et al, Intern Emerg Med. 2017 | Retrospective cohort | IMS Disease Analyzer database | Germany | January 2013 to August 2014 | 723 | NR/NA | NR/NA | NR/NA | NR/NA | 723 | |
| Coleman et al, Stroke 2017 | Retrospective cohort | Truven MarketScan Database | US | January, 2012 to June, 2015 | NR/NA | NR/NA | 981 | 2.604 | NR/NA | 1.257 | NR/NA |
| Deitelzweig et al, Curr. Med. Res. Opin. 2017 | Retrospective cohort | Humana Research Database | US | January 1, 2013 to September 30, 2015 | 14.051 | 2.474 | | 11.082 | | 8.250 | |
| Fauchier et al, Europace. 2020 | Retrospective cohort | SNIIRAM and PMSI | France | 2013 to 2014 | 36.440 | 6.385 | NR/NA | NR/NA | 8.099 | NR/NA | NR/NA |
| Friberg et al, Open Heart. 2017 | Retrospective cohort | Swedish National Databases | Sweden | December 1, 2011 to December 31, 2014 | 49.418 | 18.638 | | | | | |
| Goriacko et al, Eur J Haematol. 2018 | Retrospective cohort | Montefiore | US | May 1, 2009 to May 1, 2016 | 158 | 75 | | | | | |
| Gupta et al, BMC Cardiovasc Disord. 2019 | Retrospective cohort | US DOD Database | US | January 1, 2012 to September 30, 2015 | NR/NA | 3.691 | | 8.226 | | 7.607 | |
| Gupta et al, J Manag Care Spec Pharm. 2018 | Retrospective cohort | US DOD Database | US | January 1, 2012 to September 30, 2015 | 7.607 | 4.129 | | 11.284 | | NR/NA | NR/NA |
| Halvorsen et al, Eur Heart J Cardiovasc Pharmacother. 2017 | Retrospective cohort | Norwegian Patient Registry and Prescription Database | Norway | January 1, 2013 to June 30, 2015 | 11 427 | 7.925 | | 6.817 | | 6.506 | |

Supplementary Table 1 (Continued)

| **Study** | **Age VKA (mean, SD)** | **Age Low Dose Dab. (mean, SD)** | **Age High Dose Dab. (mean, SD)** | **Age High Dose Riv. (mean, SD)** | **Age Low Dose Riv. (mean, SD)** | **Age High Dose Api. (mean, SD)** | **Age Low Dose Api. (mean, SD)** | **Male (%) VKA** | **Male (%) Low Dose Dab.** | **Male (%) High Dose Dab.** | **Male (%) High Dose Riv.** | **Male (%) Low Dose Riv.** | **Male (%) High Dose Api.** | **Male (%) Low Dose Api.** |
| --- | --- | --- | --- | --- | --- | --- | --- | --- | --- | --- | --- | --- | --- | --- |
| Coleman et al, Intern Emerg Med. 2017 | 75.8 (9.5) | NR/NA | NR/NA | NR/NA | NR/NA | 75.8 (9.8) | | 50,6 | NR/NA | NR/NA | NR/NA | NR/NA | 49,7 | |
| Coleman et al, Stroke 2017 | NR/NA | NR/NA | 73 [63-80] | 72 [63-81] | NR/NA | 74 [63-82] | NR/NA | NR/NA | NR/NA | 51,8 | 53,1 | NR/NA | 54 | NR/NA |
| Deitelzweig et al, Curr. Med. Res. Opin. 2017 | 78.2 (9.0) | 76.8 (8.3) | | 77.2 (8.6) | | 78.0 (9.0) | | 55,2 | 55,1 | | 52,5 | | 51,5 | |
| Fauchier et al, Europace. 2020 | 89.1 (3.2) | 88.2 (2.9) | NR/NA | NR/NA | 88.6 (3.0) | NR/NA | NR/NA | 35,5 | 37,9 | NR/NA | NR/NA | 37,1 | NR/NA | NR/NA |
| Friberg et al, Open Heart. 2017 | 73,7 | 73,4 | | | | | | 55 | 54,4 | | | | | |
| Goriacko et al, Eur J Haematol. 2018 | 65.00 [59.00, 72.75] | 66.00 [61.00, 75.00] | | | | | | 59,5 | 57,3 | | | | | |
| Gupta et al, BMC Cardiovasc Disord. 2019 | NR/NA | 74.0 (9.5) | | 76.5 (9.3) | | 76.5 (9.5) | | NR/NA | 60,8 | | 58,2 | | 58,2 | |
| Gupta et al, J Manag Care Spec Pharm. 2018 | 76.6 (9.8) | 73.0 (9.9) | | 75.3 (9.5) | | NR/NA | NR/NA | 58,2 | 61,5 | | 56,8 | | NR/NA | NR/NA |
| Halvorsen et al, Eur Heart J Cardiovasc Pharmacother. 2017 | 74.6 (11.9) | 70.8 (11.3) | | 74.7 (10.7) | | 74.5 (11.1) | | 59 | 62 | | 54,4 | | 55 | |

Supplementary Table 1 (Continued)

| **Study** | **Design** | **Setting/Data source** | **Country/ Region** | **Study Enrollement Period** | **No. VKA** | **No. Dabigatran (Low Dose)** | **No. Dabigatran (High Dose)** | **No. Rivaroxaban (High Dose)** | **No. Rivaroxaban (Low Dose)** | **No. Apixaban (High Dose)** | **No. Apixaban (Low Dose)** |
| --- | --- | --- | --- | --- | --- | --- | --- | --- | --- | --- | --- |
| Hernandez et al, Am J Cardiol. 2017 | Retrospective cohort | Centers for Medicare & Medicaid Services (CMS) | US | January 1, 2013 and December 31, 2014 | 12.353 | 1.415 | | 5.139 | | 2.358 | |
| Hernandez et al, JAMA Intern Med. 2015 | Retrospective cohort | Centers for Medicare & Medicaid Services (CMS) | US | October 1, 2010 to October 31, 2011 | 8.102 | 1.302 | | NR/NA | NR/NA | NR/NA | NR/NA |
| Ho et al, Clin. Cardiol. 2012 | Retrospective cohort | Prince of Wales Hospital | Hong Kong | January, 2010 to November, 2011 | 122 | 122 | | NR/NA | NR/NA | NR/NA | NR/NA |
| Hohnloser et al, Thromb. Haemost. 2018 | Retrospective cohort | Institute for Applied Health Research | Germany | January 1, 2013 to December 31, 2015 | 23.823 | 2.596 | 2.526 | 15.923 | 6.220 | 6.376 | 3.741 |
| Huybrechts et al, Clin Pharmacol Ther. 2020 | Retrospective cohort | 2 Databases (Optum, Marketscan) | US | October 2010 to September 2015 | 29.448 | 29.448 | | NR/NA | NR/NA | NR/NA | NR/NA |
| Jeong et al, Chonnam Med. J. 2019 | Retrospective cohort | Chonnam National University Hospital | Korea | January, 2014 to December, 2016 | 804 | NR/NA | NR/NA | 804 | | NR/NA | NR/NA |
| Kohsaka et al, Curr Med Res Opin. 2017 | Retrospective cohort | Medical Data Vision Co. Ltd | Japan | March 1, 2011 to March 31, 2016 | NR/NA | 5.090 | | NR/NA | 6.726 | 5977 | |
| Kohsaka et al, Curr Med Res Opin. 2018 | Retrospective cohort | Medical Data Vision Co. Ltd | Japan | March 1, 2011 to June 30, 2017 | 11.972 | NR/NA | NR/NA | NR/NA | NR/NA | 11.972 | |
| Korenstra et al, Europace 2016 | Retrospective cohort | Martini Hospital Groningen | Netherlands | January 1, 2010 to December 31, 2012 | 383 | 383 | | NR/NA | NR/NA | NR/NA | NR/NA |

Supplementary Table 1 (Continued)

| **Study** | **Age VKA (mean, SD)** | **Age Low Dose Dab. (mean, SD)** | **Age High Dose Dab. (mean, SD)** | **Age High Dose Riv. (mean, SD)** | **Age Low Dose Riv. (mean, SD)** | **Age High Dose Api. (mean, SD)** | **Age Low Dose Api. (mean, SD)** | **Male (%) VKA** | **Male (%) Low Dose Dab.** | **Male (%) High Dose Dab.** | **Male (%) High Dose Riv.** | **Male (%) Low Dose Riv.** | **Male (%) High Dose Api.** | **Male (%) Low Dose Api.** |
| --- | --- | --- | --- | --- | --- | --- | --- | --- | --- | --- | --- | --- | --- | --- |
| Hernandez et al, Am J Cardiol. 2017 | 76.0 (10.3) | 74.9 (8.7) | | 76.4 (8.6) | | 77.4 (8.6) | | 43,1 | 47 | | 43,7 | | 42,5 | |
| Hernandez et al, JAMA Intern Med. 2015 | 75.6 (9.5) | 75.1 (10.2) | | NR/NA | NR/NA | NR/NA | NR/NA | 41 | 42,1 | | NR/NA | NR/NA | NR/NA | NR/NA |
| Ho et al, Clin. Cardiol. 2012 | 70.1 (10.3) | 70.0 (11.4) | | NR/NA | NR/NA | NR/NA | NR/NA | 52,5 | 55,7 | | NR/NA | NR/NA | NR/NA | NR/NA |
| Hohnloser et al, Thromb. Haemost. 2018 | 75.2 (9.5) | 77.3 (9.5) | 66.0 (10.7) | 69.3 (11.6) | 79.1 (9.0) | 70.4 (10.9) | 81.6 (8.2) | 53,3 | 46,9 | 63,5 | 58,3 | 45,5 | 57,6 | 40,7 |
| Huybrechts et al, Clin Pharmacol Ther. 2020 | 67.65 12.07 | 67.84 11.87 | | NR/NA | NR/NA | NR/NA | NR/NA | 64,15 | 63,79 | | NR/NA | NR/NA | NR/NA | NR/NA |
| Jeong et al, Chonnam Med. J. 2019 | 70.4 (10.2) | NR/NA | NR/NA | 71.4 (10.5) | | NR/NA | NR/NA | 60,4 | NR/NA | NR/NA | 63,3 | | NR/NA | NR/NA |
| Kohsaka et al, Curr Med Res Opin. 2017 | NR/NA | 73.1 ± 9.9 | | NR/NA | 75.8 ± 10.0 | 77.4 ± 10.0 | | NR/NA | 65,9 | | NR/NA | 62 | 59,4 | |
| Kohsaka et al, Curr Med Res Opin. 2018 | 77.7 (10.0) | NR/NA | NR/NA | NR/NA | NR/NA | 77.6 (10.0) | | 58,5 | NR/NA | NR/NA | NR/NA | NR/NA | 58,3 | |
| Korenstra et al, Europace 2016 | 72.3 (9.3) | 70.6 (8.9) | | NR/NA | NR/NA | NR/NA | NR/NA | 51,2 | 53,5 | | NR/NA | NR/NA | NR/NA | NR/NA |

Supplementary Table 1 (Continued)

| **Study** | **Design** | **Setting/Data source** | **Country/ Region** | **Study Enrollement Period** | **No. VKA** | **No. Dabigatran (Low Dose)** | **No. Dabigatran (High Dose)** | **No. Rivaroxaban (High Dose)** | **No. Rivaroxaban (Low Dose)** | **No. Apixaban (High Dose)** | **No. Apixaban (Low Dose)** |
| --- | --- | --- | --- | --- | --- | --- | --- | --- | --- | --- | --- |
| Laliberté et al, Curr. Med. Res. Opin. 2014 | Retrospective cohort | Symphony Health Solutions’ Database | US | May, 2011 to July, 2012 | 14.616 | NR/NA | NR/NA | 3.654 | | NR/NA | NR/NA |
| Lamberts et al, J Am Heart Assoc. 2017 | Retrospective cohort | National Patient Registry | Denmark | August 22, 2011 to December 31, 2015 | 24.230 | 15.413 | | 6.715 | | 7.963 | |
| Larsen et al, Am J Med. 2014 | Retrospective cohort | Danish Registries | Denmark | August 1, 2009 to May 30, 2013 | 8.504 | 2.038 | 2.214 | NR/NA | NR/NA | NR/NA | NR/NA |
| Larsen et al, BMJ 2016 | Retrospective cohort | Danish National Databases | Denmark | August 1, 2011 to 30 November 30, 2015 | 35.436 | NR/NA | 12.701 | 7.192 | NR/NA | 6.349 | NR/NA |
| Laugesen et al, Thromb. J. 2019 | Retrospective cohort | Danish National Databases | Denmark | August 22, 2011 to June 30, 2017 | 1.008 | 552 | | | | | |
| Lee et al, Int J Cardiol. 2018 | Retrospective cohort | Taiwan's National Health Insurance Research Database | Taiwan | January 1, 2010 to December 31, 2016 | 16.000 | 14.971 | NR/NA | NR/NA | NR/NA | NR/NA | NR/NA |
| Lee et al, Stroke 2019 (2) | Retrospective cohort | Korean National Health Insurance Service Database | Korea | January, 2015 to December, 2017 | 25.420 | 17.745 | | 35.965 | | 22.177 | |
| Li et al, Thromb Haemost. 2017 | Retrospective cohort | 4 Databases (Truven MarketScan, IMS, Optum, Humana) | US | January 1, 2013 to September 30, 2015 | 38.470 | NR/NA | NR/NA | NR/NA | NR/NA | 38.470 | |
| Lin et al, J Med Econ. 2017 | Retrospective cohort | IMS Pharmetrics Plus database | US | January 1, 2013 and September 30, 2015 | 4.847 | 2.684 | | 4.062 | | NR/NA | NR/NA |

Supplementary Table 1 (Continued)

| **Study** | **Age VKA (mean, SD)** | **Age Low Dose Dab. (mean, SD)** | **Age High Dose Dab. (mean, SD)** | **Age High Dose Riv. (mean, SD)** | **Age Low Dose Riv. (mean, SD)** | **Age High Dose Api. (mean, SD)** | **Age Low Dose Api. (mean, SD)** | **Male (%) VKA** | **Male (%) Low Dose Dab.** | **Male (%) High Dose Dab.** | **Male (%) High Dose Riv.** | **Male (%) Low Dose Riv.** | **Male (%) High Dose Api.** | **Male (%) Low Dose Api.** |
| --- | --- | --- | --- | --- | --- | --- | --- | --- | --- | --- | --- | --- | --- | --- |
| Laliberté et al, Curr. Med. Res. Opin. 2014 | 73.7 (8.3) | NR/NA | NR/NA | 73.3 (8.4) | | NR/NA | NR/NA | 48,5 | NR/NA | NR/NA | 40 | | NR/NA | NR/NA |
| Lamberts et al, J Am Heart Assoc. 2017 | 73 [65, 80] | 71 [65, 79] | | 74 [67, 83] | | 76 [68, 84] | | 58,4 | 56,7 | | 52 | | 50,8 | |
| Larsen et al, Am J Med. 2014 | 74 [67-81] | 82 [77-86] | 69 [64-73] | NR/NA | NR/NA | NR/NA | NR/NA | 61,6 | 45,6 | 64,8 | NR/NA | NR/NA | NR/NA | NR/NA |
| Larsen et al, BMJ 2016 | 72.4 [64.7-79.8] | NR/NA | 67.6 [62.0-72.4] | 71.8 (65.7-78.9) | NR/NA | 71.3 [65.8-77.2] | NR/NA | 58,8 | NR/NA | 66,1 | 56,9 | NR/NA | 60,3 | NR/NA |
| Laugesen et al, Thromb. J. 2019 | 78 [71-84] | 80 [72-86] | | | | | | 64 | 56,9 | | | | | |
| Lee et al, Int J Cardiol. 2018 | 78 (10) | 78 (10) | NR/NA | NR/NA | NR/NA | NR/NA | NR/NA | 52 | 52 | NR/NA | NR/NA | NR/NA | NR/NA | NR/NA |
| Lee et al, Stroke 2019 (2) | 71.2 (11.1) | 70.9 (10.5) | | 70.9 (10.7) | | 70.9 (11.0) | | 73 [65-79] | 72 [65-78] | | 72 [65-78] | | 72 [65-79] | |
| Li et al, Thromb Haemost. 2017 | 70.9 (11.9) | NR/NA | NR/NA | NR/NA | NR/NA | 70.9 (12.0) | | 59,8 | NR/NA | NR/NA | NR/NA | NR/NA | 59,7 | |
| Lin et al, J Med Econ. 2017 |  | 63.0 (9.3) | | 62.0 (8.4) | | NR/NA | NR/NA | 70,6 | 72,5 | | 70,4 | | NR/NA | NR/NA |

Supplementary Table 1 (Continued)

| **Study** | **Design** | **Setting/Data source** | **Country/ Region** | **Study Enrollement Period** | **No. VKA** | **No. Dabigatran (Low Dose)** | **No. Dabigatran (High Dose)** | **No. Rivaroxaban (High Dose)** | **No. Rivaroxaban (Low Dose)** | **No. Apixaban (High Dose)** | **No. Apixaban (Low Dose)** |
| --- | --- | --- | --- | --- | --- | --- | --- | --- | --- | --- | --- |
| Lip et al, Stroke 2018 | Retrospective cohort | 5 Databases (Medicare/Medicaid, Truven MarketScan, IMS, Optum, Humana) | US | January 1, 2013 to September 30, 2015 | 167.413 | 37.724 | | 153.002 | | 108.852 | |
| Lip et al, Thromb Haemost. 2016 | Retrospective cohort | Truven MarketScan Database | US | January 1, 2013 to December 31, 2014 | NR/NA | 4.515 | | 12.625 | | 6.964 | |
| Loo et al, BMJ Open. 2018 | Retrospective cohort | Clinical Practice Research Datalink | UK | August 1, 2011 to September 30, 2016 | 6.731 | 6.731 | | | | | |
| Mueller et al, Pragmat Obs Res. 2018 | Retrospective cohort | 3 German Health Insurance Databases (AOK) | Germany | January 1, 2010 to June 30, 2014 | 37.439 | 37.439 | | | | | |
| Nielsen et al, BMJ 2017 | Retrospective cohort | Danish National Databases | Denmark | August, 2011 to February, 2016 | 38.893 | 8.875 | NR/NA | NR/NA | 3.476 | NR/NA | 4.400 |
| Noseworthy et al, Chest. 2016 | Retrospective cohort | Optum Labs Data Warehouse | US | October 1, 2010 to February 28, 2015 | NR/NA | NR/NA | NR/NA | NR/NA | NR/NA | NR/NA | NR/NA |
| Okumura et al, Circ J. 2018 | Retrospective cohort | Multicenter Registry (SAKURA AF Registry) | Japan | September 1, 2013 and December 31, 2015 | 1.561 | 456 | | 761 | | 428 | |
| Palamaner et al, Circ Cardiovasc Qual Outcomes. 2017 | Retrospective cohort | Centers for Medicare & Medicaid Services (CMS) | US | November 1, 2011 to October 31, 2013 | 18.646 | NR/NA | 19 | 18.646 | NR/NA | NR/NA | NR/NA |
| Ramagopalan et al, J Comp Eff Res. 2018 | Retrospective cohort | Database of Italian Local Health Hnits (LHUs) | Italy | January 1, 2012 to December 31, 2015 | 8393 | NR/NA | NR/NA | NR/NA | NR/NA | 1521 | |

Supplementary Table 1 (Continued)

| **Study** | **Age VKA (mean, SD)** | **Age Low Dose Dab. (mean, SD)** | **Age High Dose Dab. (mean, SD)** | **Age High Dose Riv. (mean, SD)** | **Age Low Dose Riv. (mean, SD)** | **Age High Dose Api. (mean, SD)** | **Age Low Dose Api. (mean, SD)** | **Male (%) VKA** | **Male (%) Low Dose Dab.** | **Male (%) High Dose Dab.** | **Male (%) High Dose Riv.** | **Male (%) Low Dose Riv.** | **Male (%) High Dose Api.** | **Male (%) Low Dose Api.** |
| --- | --- | --- | --- | --- | --- | --- | --- | --- | --- | --- | --- | --- | --- | --- |
| Lip et al, Stroke 2018 | 76.7 (9.4) | 72.8 (10.7) | | 73.4 (10.8) | | 75.3 (10.4) | | 52,9 | 57,2 | | 55,3 | | 51,7 | |
| Lip et al, Thromb Haemost. 2016 | NR/NA | 66.9 (12.2) | | 69.7 (11.9) | | 69.1 (12.3) | | NR/NA | 60,9 | | 64,2 | | 61 | |
| Loo et al, BMJ Open. 2018 | 74.91 (10.29) | 74.91 (10.29) | | | | | | 55,3 | 55,3 | | | | | |
| Mueller et al, Pragmat Obs Res. 2018 | 78.16 (7.37) | 78.21 (7.40) | | | | | | 47,59 | 47,49 | | | | | |
| Nielsen et al, BMJ 2017 | 71.0 (12.6) | 79.9 (9.0) | NR/NA | NR/NA | 77.9 (13.5) | NR/NA | 83.9 (8.2) | 59,6 | 46,3 | NR/NA | NR/NA | 46,8 | NR/NA | 39,4 |
| Noseworthy et al, Chest. 2016 | NR/NA | NR/NA | NR/NA | NR/NA | NR/NA | NR/NA | NR/NA | NR/NA | NR/NA | NR/NA | NR/NA | NR/NA | NR/NA | NR/NA |
| Okumura et al, Circ J. 2018 | 72.2 (9.3) | 70.9 (9.5) | | 71.5 (9.1) | | 73.2 (10.1) | | 76,5 | 75,2 | | 73,2 | | 64,2 | |
| Palamaner et al, Circ Cardiovasc Qual Outcomes. 2017 | 75,98 | NR/NA | 76,43 | 76,1 | NR/NA | NR/NA | NR/NA | 41 | NR/NA | 41 | 41 | NR/NA | NR/NA | NR/NA |
| Ramagopalan et al, J Comp Eff Res. 2018 | 78 (20–104) | NR/NA | NR/NA | NR/NA | NR/NA | 79 (35–100) | | 52,3 | NR/NA | NR/NA | NR/NA | NR/NA | 49,2 | |

Supplementary Table 1 (Continued)

| **Study** | **Design** | **Setting/Data source** | **Country/ Region** | **Study Enrollement Period** | **No. VKA** | **No. Dabigatran (Low Dose)** | **No. Dabigatran (High Dose)** | **No. Rivaroxaban (High Dose)** | **No. Rivaroxaban (Low Dose)** | **No. Apixaban (High Dose)** | **No. Apixaban (Low Dose)** |
| --- | --- | --- | --- | --- | --- | --- | --- | --- | --- | --- | --- |
| Russo-Alvarez et al, Ann. Pharmacother. 2018 | Retrospective cohort | Cleveland Clinic Health System (CCHS) | US | January 1, 2012 to July 1, 2016 | 472 | NR/NA | NR/NA | 472 | | NR/NA | NR/NA |
| Siontis et al, Circulation. 2018 | Retrospective cohort | US Renal Data System (USRDS) | US | October 2010 to December 2015 | 7.053 | NR/NA | NR/NA | NR/NA | NR/NA | 2.351 | |
| Sjogren et al, PLoS One. 2017 | Retrospective cohort | Swedish National Databases | Sweden | July 1, 2011 to December 31, 2014 | 12.694 | 12.694 | | | | | |
| Vinogradova et al, BMJ 2018 | Prospective cohort | Qresearch or Clinical Practice Research Datalink | UK | January, 2011 to October, 2016 | 61.646 | 2.207 | | 21.316 | | 7.622 | |
| Wang et al, J Am Heart Assoc. 2018 | Retrospective cohort | Chang Gung Hospital System | Taiwan | 2009 to 2016 | NR/NA | NR/NA | NR/NA | NR/NA | NR/NA | NR/NA | NR/NA |
| Weir et al, Clin Nephrol. 2018 | Retrospective cohort | IMS Disease Analyzer | US | November, 2011 to June, 2015 | 1.012 | NR/NA | NR/NA | 931 | | NR/NA | NR/NA |
| Weir et al, Curr. Med. Res. Opin. 2017 | Retrospective cohort | Optum Labs Data Warehouse | US | May, 2011 to August, 2014 | 1.961 | NR/NA | NR/NA | 1.797 | | NR/NA | NR/NA |
| Yamashita et al, Circ. J. 2017 | Prospective cohort | Fushimi AF Registry | Japan | March, 2011 to November, 2015 | 1.728 | 270 | | | | | |
| Yao et al, JAHA 2016 | Retrospective cohort | Optum Labs Data Warehouse | US | October 1, 2010 to June 30, 2015 | NR/NA | 28.614 | | 32.350 | | 15.390 | |

Supplementary Table 1 (Continued)

| **Study** | **Age VKA (mean, SD)** | **Age Low Dose Dab. (mean, SD)** | **Age High Dose Dab. (mean, SD)** | **Age High Dose Riv. (mean, SD)** | **Age Low Dose Riv. (mean, SD)** | **Age High Dose Api. (mean, SD)** | **Age Low Dose Api. (mean, SD)** | **Male (%) VKA** | **Male (%) Low Dose Dab.** | **Male (%) High Dose Dab.** | **Male (%) High Dose Riv.** | **Male (%) Low Dose Riv.** | **Male (%) High Dose Api.** | **Male (%) Low Dose Api.** |
| --- | --- | --- | --- | --- | --- | --- | --- | --- | --- | --- | --- | --- | --- | --- |
| Russo-Alvarez et al, Ann. Pharmacother. 2018 | 73.6 (11.9) | NR/NA | NR/NA | 73.6 (11.5) | | NR/NA | NR/NA | 63,6 | NR/NA | NR/NA | 61,2 | | NR/NA | NR/NA |
| Siontis et al, Circulation. 2018 | 68.04 (11.90) | NR/NA | NR/NA | NR/NA | NR/NA | 68.87 (11.49) | | 54,9 | NR/NA | NR/NA | NR/NA | NR/NA | 54,4 | |
| Sjogren et al, PLoS One. 2017 | 72.3 (10.3) | 72.2 (10.3) | | | | | | 57,1 | 58,2 | | | | | |
| Vinogradova et al, BMJ 2018 | 66.45 (15.67) | 71.6 (12.87) | | 68.02 (15.8) | | 73.95 (13.59) | | 53,51 | 53,1 | | 48,6 | | 51,6 | |
| Wang et al, J Am Heart Assoc. 2018 | NR/NA | NR/NA | NR/NA | NR/NA | NR/NA | NR/NA | NR/NA | NR/NA | NR/NA | NR/NA | NR/NA | NR/NA | NR/NA | NR/NA |
| Weir et al, Clin Nephrol. 2018 | 64.7 (10) | NR/NA | NR/NA | 64.2 (9.8) | | NR/NA | NR/NA | 69,8 | NR/NA | NR/NA | 70 | | NR/NA | NR/NA |
| Weir et al, Curr. Med. Res. Opin. 2017 | NR/NA | NR/NA | NR/NA | NR/NA | NR/NA | NR/NA | NR/NA | 55,86 | NR/NA | NR/NA | 56,31 | | NR/NA | NR/NA |
| Yamashita et al, Circ. J. 2017 | 74.4 (9.1) | 72.0 (10.3) | | | | | | 62 | 62 | | | | | |
| Yao et al, JAHA 2016 | NR/NA | 70 [62-78] | | 72 [64-79] | | 73 [66-81] | | NR/NA | 60,3 | | 56,8 | | 53,1 | |

Supplementary Table 1 (Continued)

| **Study** | **Design** | **Setting/Data source** | **Country/ Region** | **Study Enrollement Period** | **No. VKA** | **No. Dabigatran (Low Dose)** | **No. Dabigatran (High Dose)** | **No. Rivaroxaban (High Dose)** | **No. Rivaroxaban (Low Dose)** | **No. Apixaban (High Dose)** | **No. Apixaban (Low Dose)** |
| --- | --- | --- | --- | --- | --- | --- | --- | --- | --- | --- | --- |
| Yap et al, Clin. Appl. Thromb. Hemost. 2016 | Retrospective cohort | Malaysia’s National Heart Institute | Malaysia | January, 2009 to December, 2013 | 500 | 205 | 295 | NR/NA | NR/NA | NR/NA | NR/NA |

Supplementary Table 1 (Continued)

| **Study** | **Age VKA (mean, SD)** | **Age Low Dose Dab. (mean, SD)** | **Age High Dose Dab. (mean, SD)** | **Age High Dose Riv. (mean, SD)** | **Age Low Dose Riv. (mean, SD)** | **Age High Dose Api. (mean, SD)** | **Age Low Dose Api. (mean, SD)** | **Male (%) VKA** | **Male (%) Low Dose Dab.** | **Male (%) High Dose Dab.** | **Male (%) High Dose Riv.** | **Male (%) Low Dose Riv.** | **Male (%) High Dose Api.** | **Male (%) Low Dose Api.** |
| --- | --- | --- | --- | --- | --- | --- | --- | --- | --- | --- | --- | --- | --- | --- |
| Yap et al, Clin. Appl. Thromb. Hemost. 2016 | 66.8 (11.3) | 65.3 (11.3) | | NR/NA | NR/NA | NR/NA | NR/NA | 60,2 | 62 | | NR/NA | NR/NA | NR/NA | NR/NA |

**Supplementary Figure 1**

Supplementary Figure 1A Supplementary Figure 1B


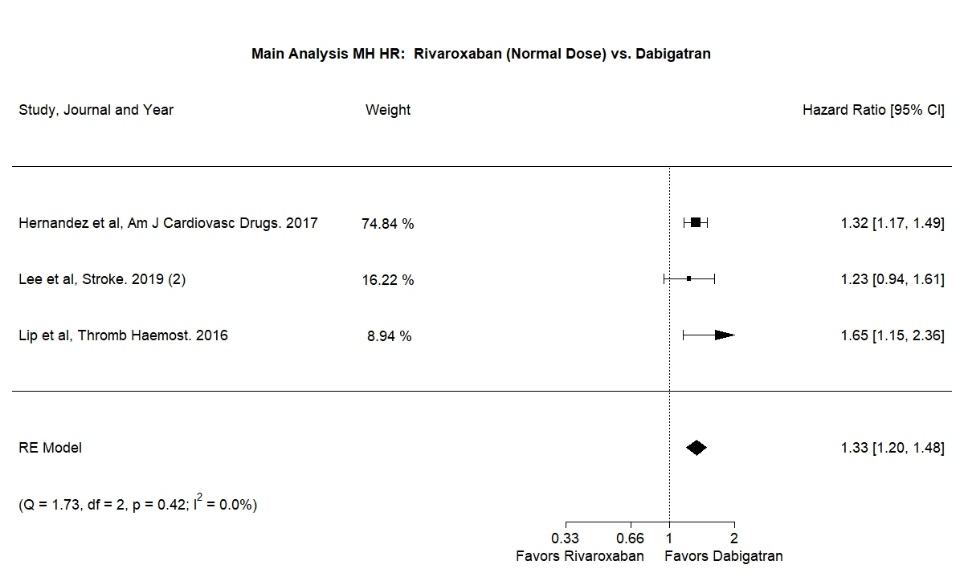

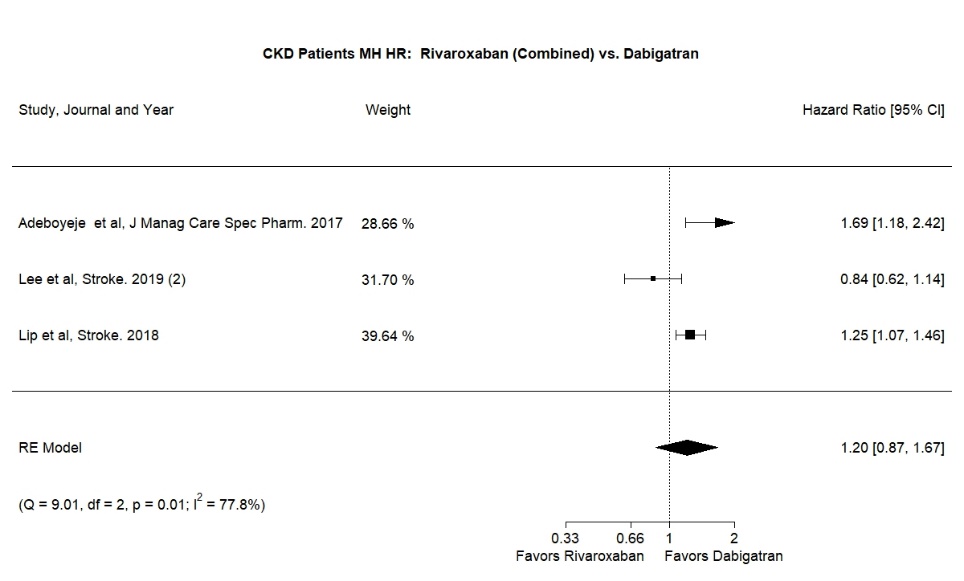


Supplementary Figure 1C Supplementary Figure 1D


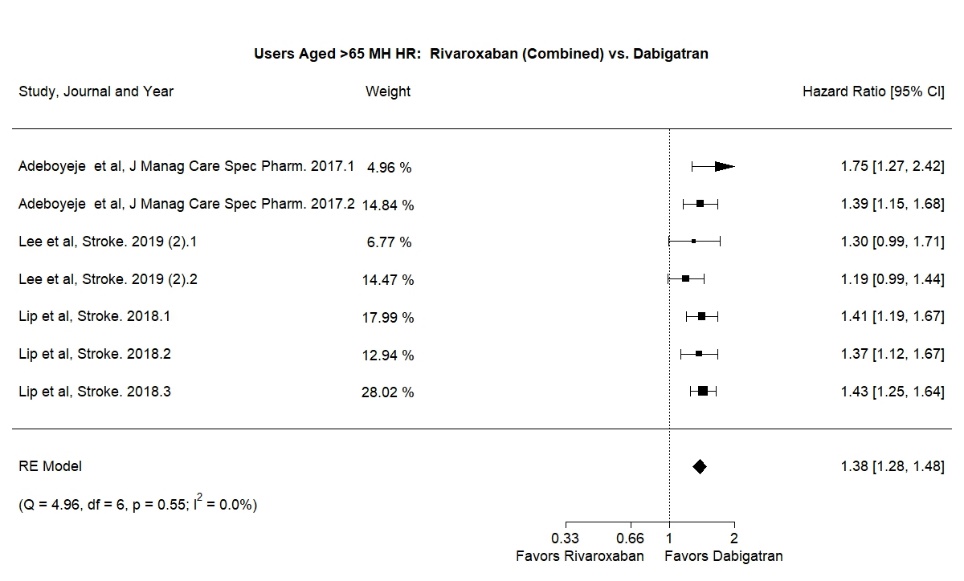

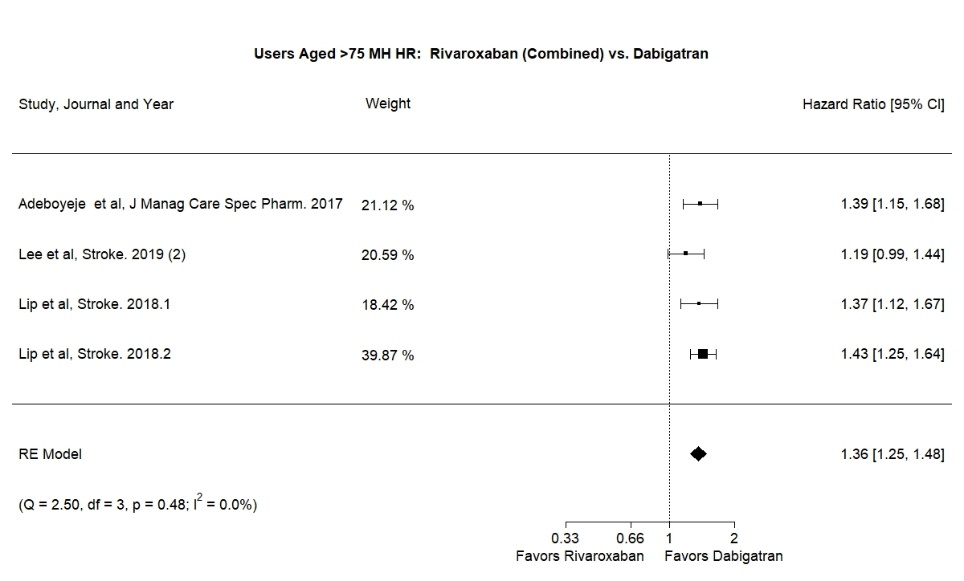


Supplementary Figure 1E


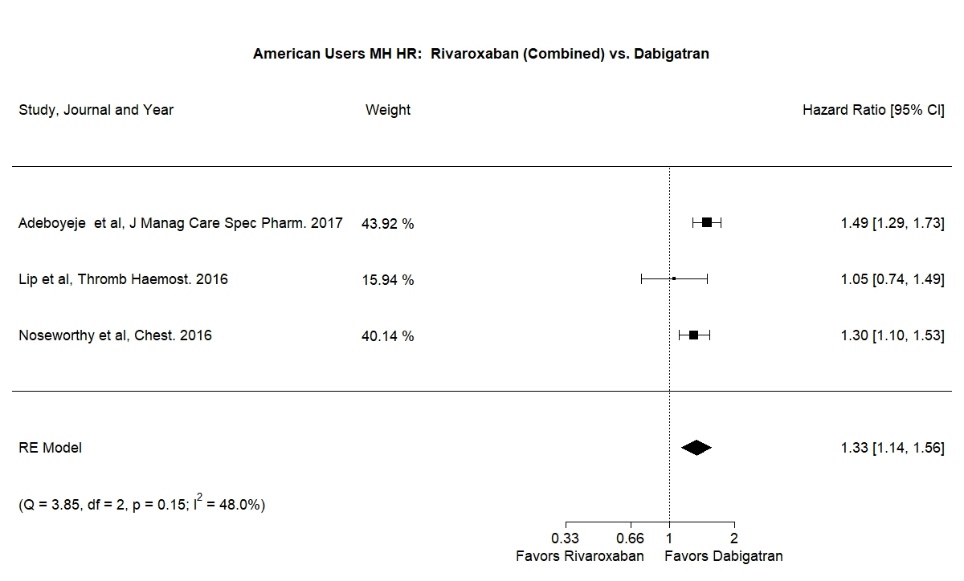


**Supplementary Figure 2**

Supplementary Figure 2A Supplementary Figure 2B


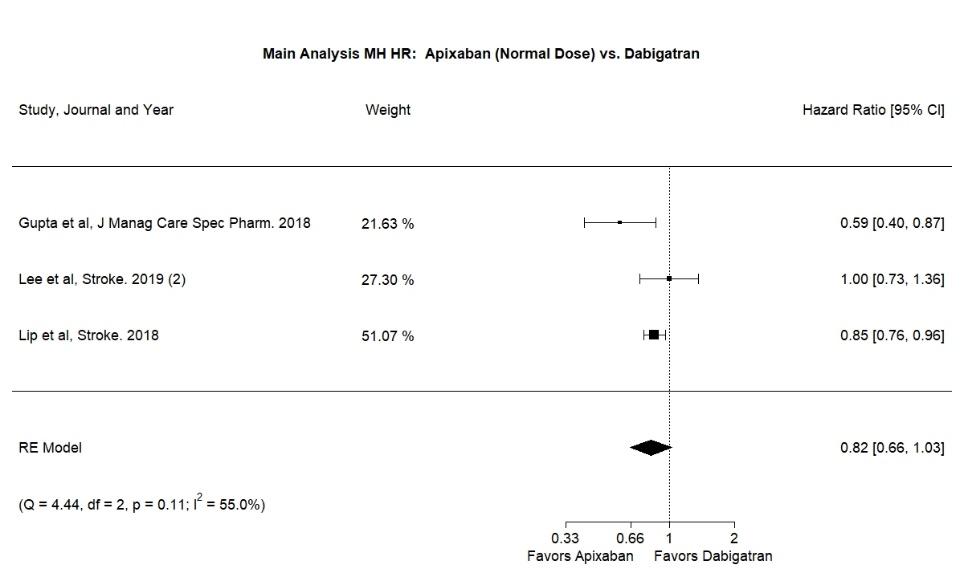

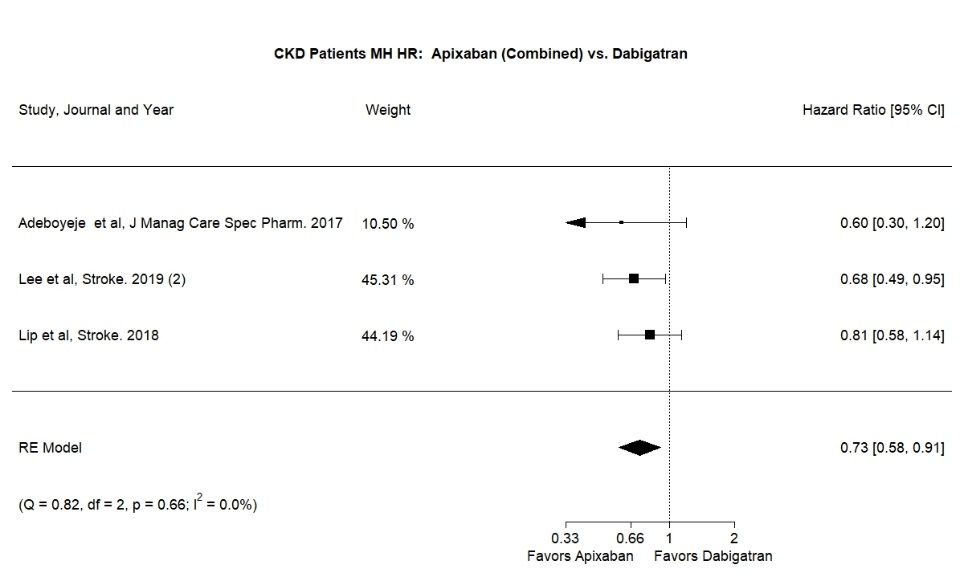


Supplementary Figure 2C Supplementary Figure 2D


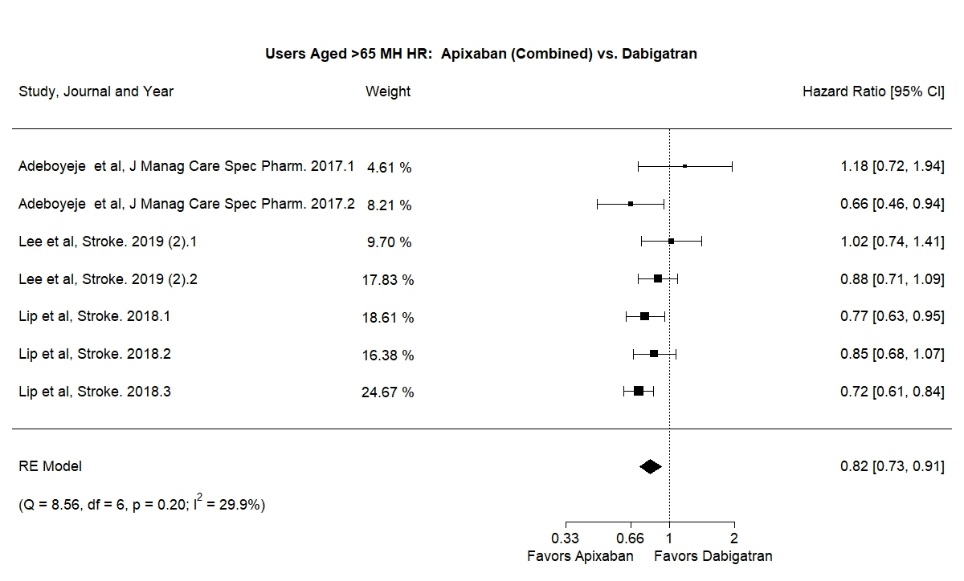

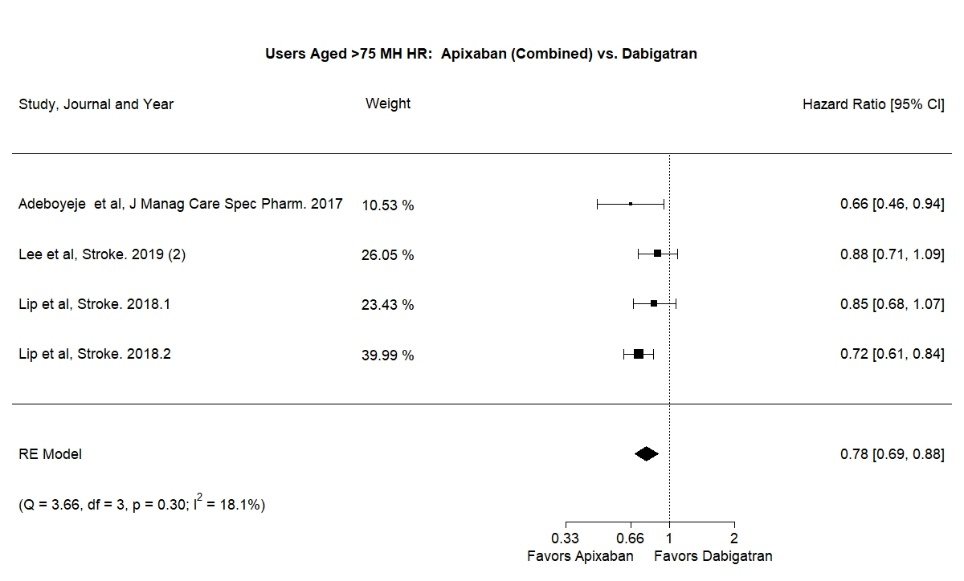


Supplementary Figure 2E Supplementary Figure 2F


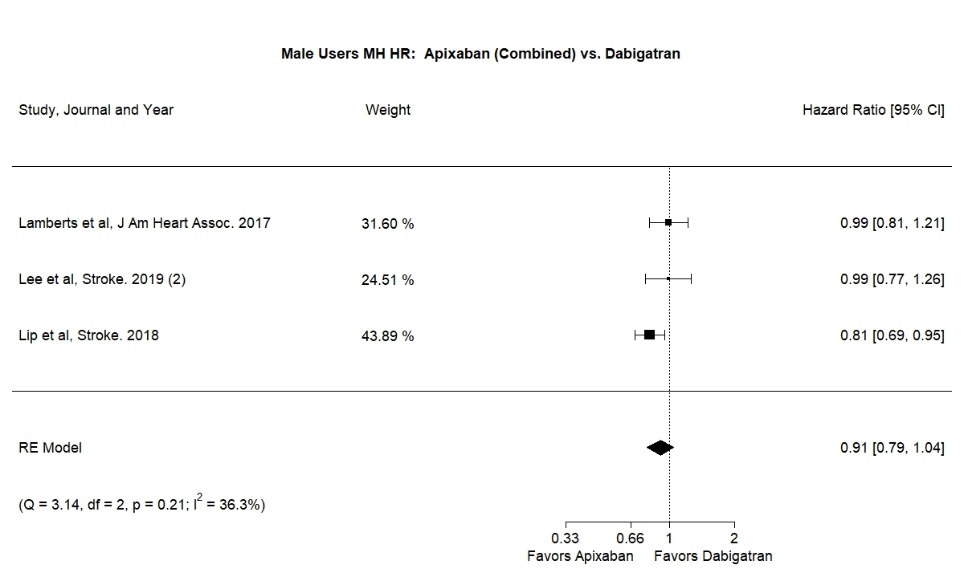

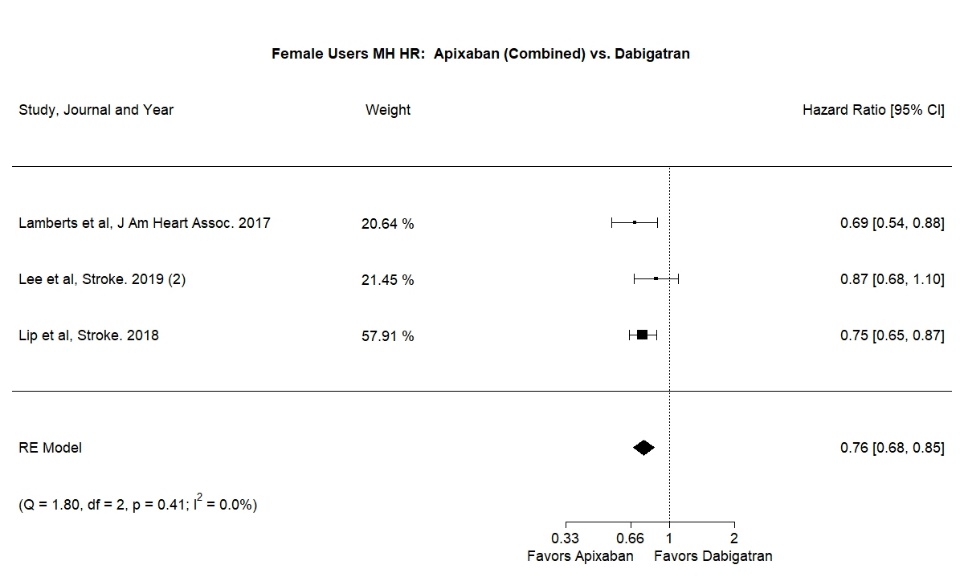


Supplementary Figure 2G


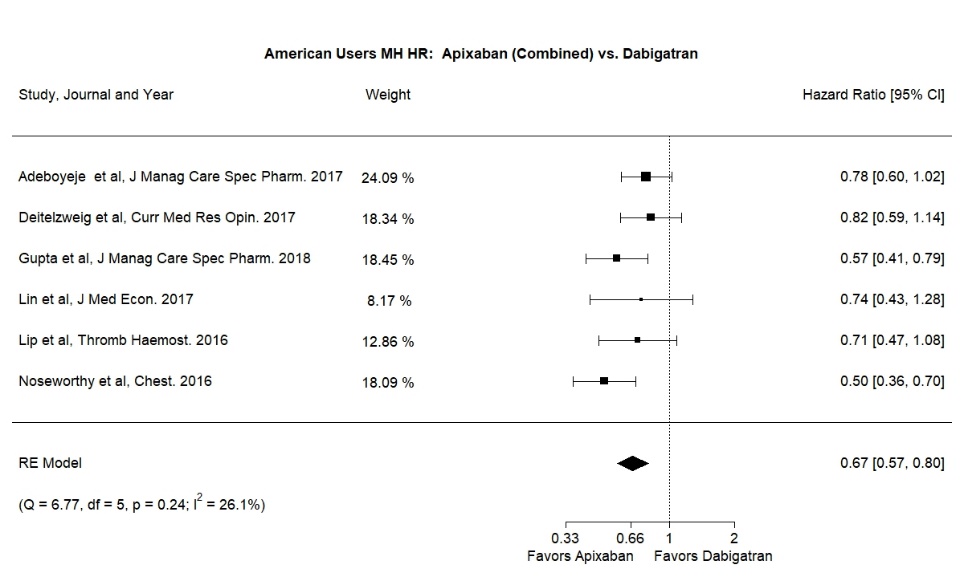


**Supplementary Figure 3**

Supplementary Figure 3A Supplementary Figure 3B

**
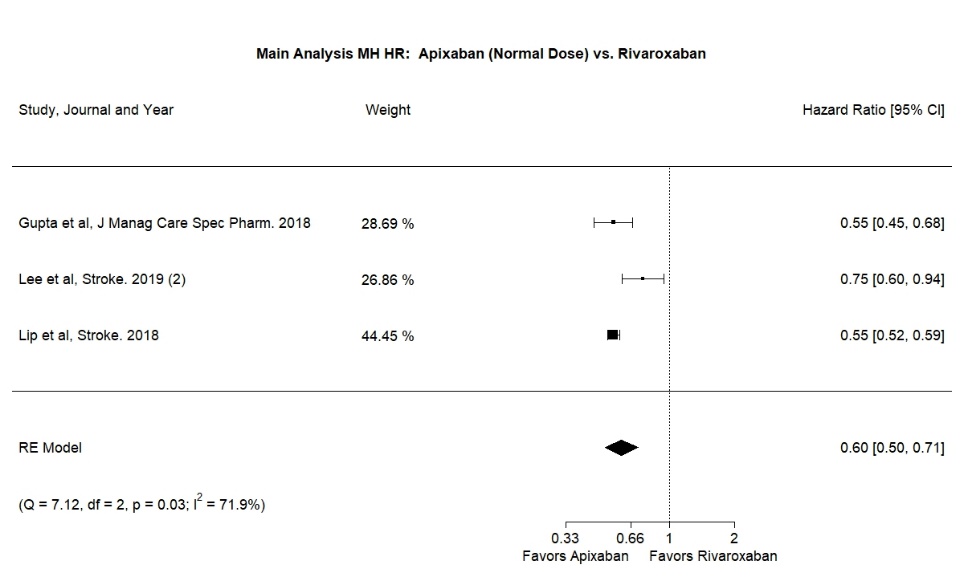

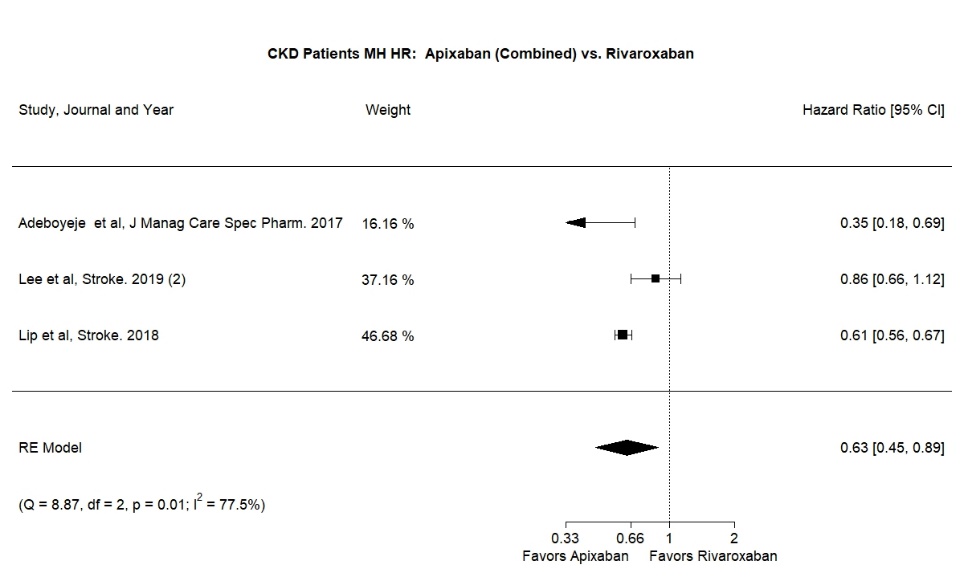
**

Supplementary Figure 3C Supplementary Figure 3D

**
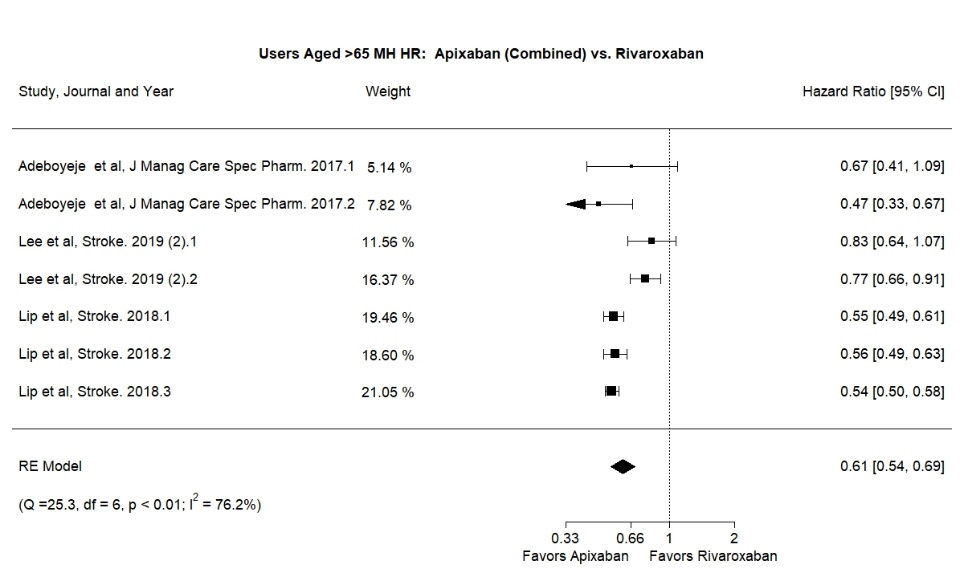

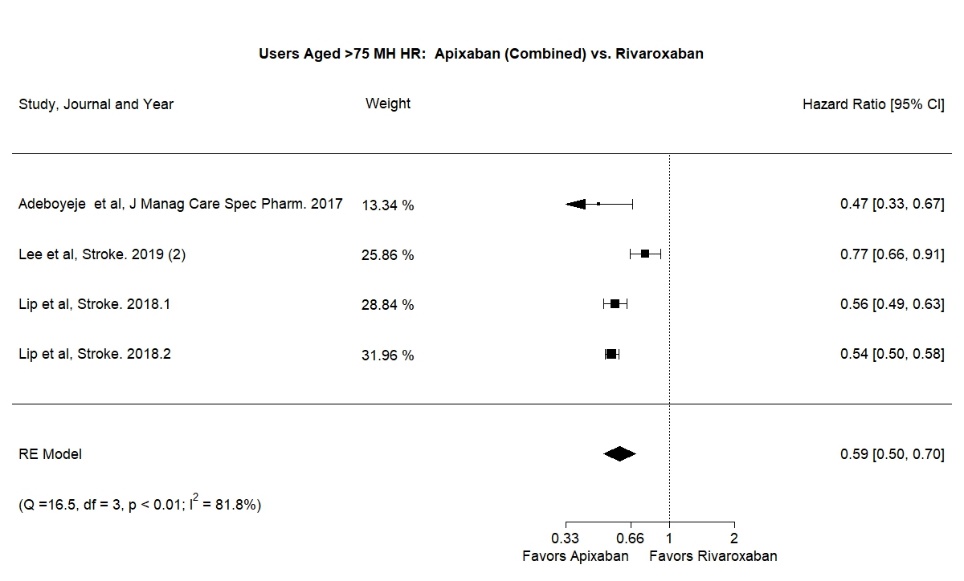
**

Supplementary Figure 3E Supplementary Figure 3F

**
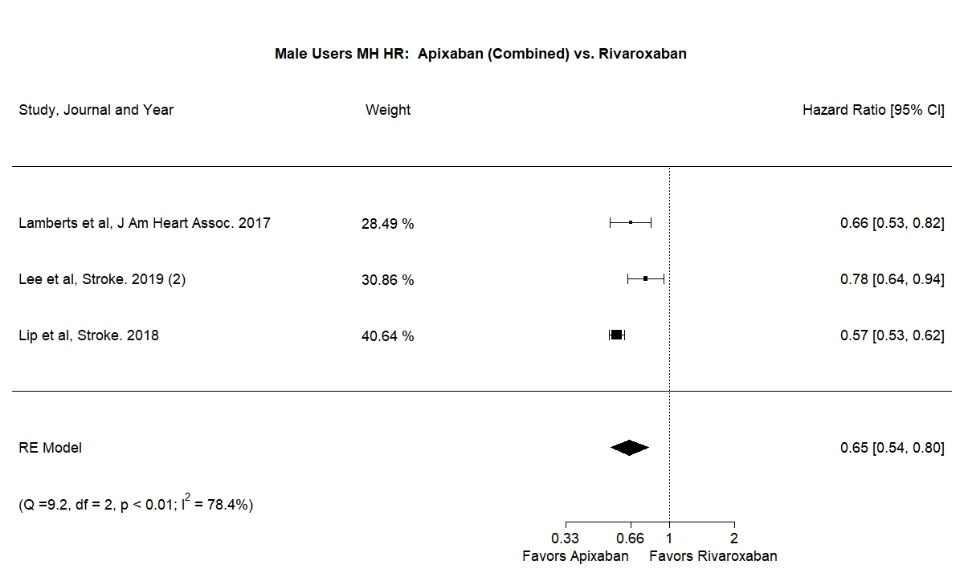

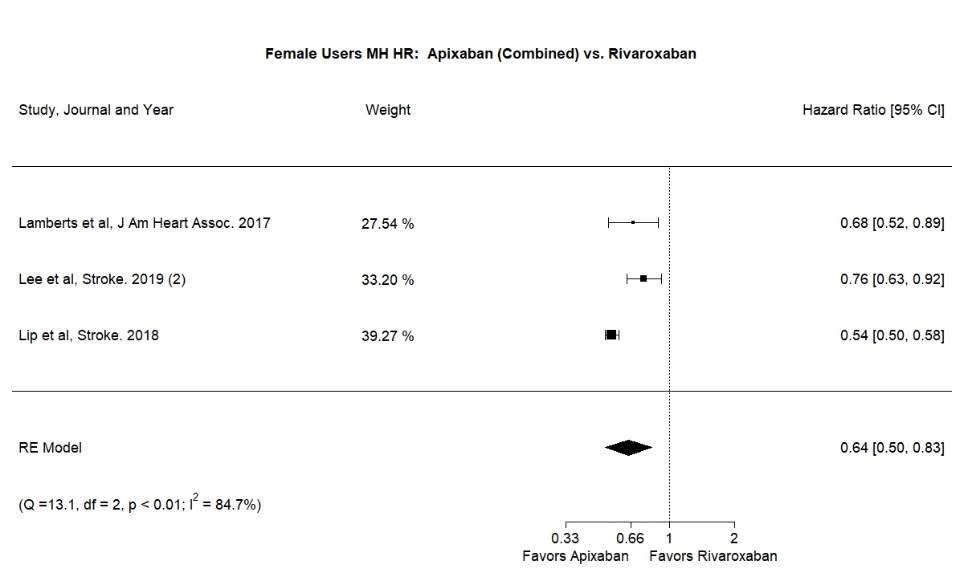
**

Supplementary Figure 3G

**
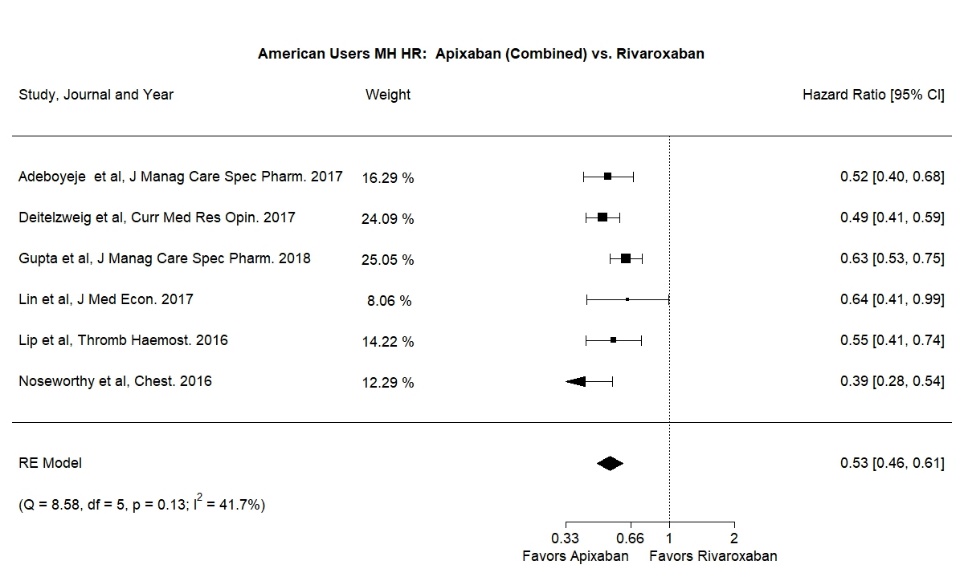
**

**Supplementary Figure 4**

Supplementary Figure 4A Supplementary Figure 4B


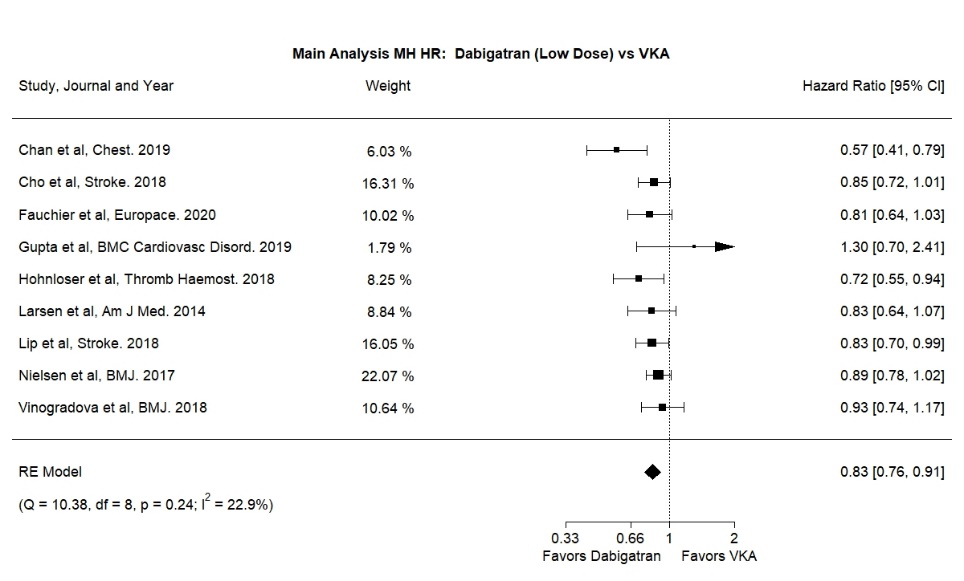

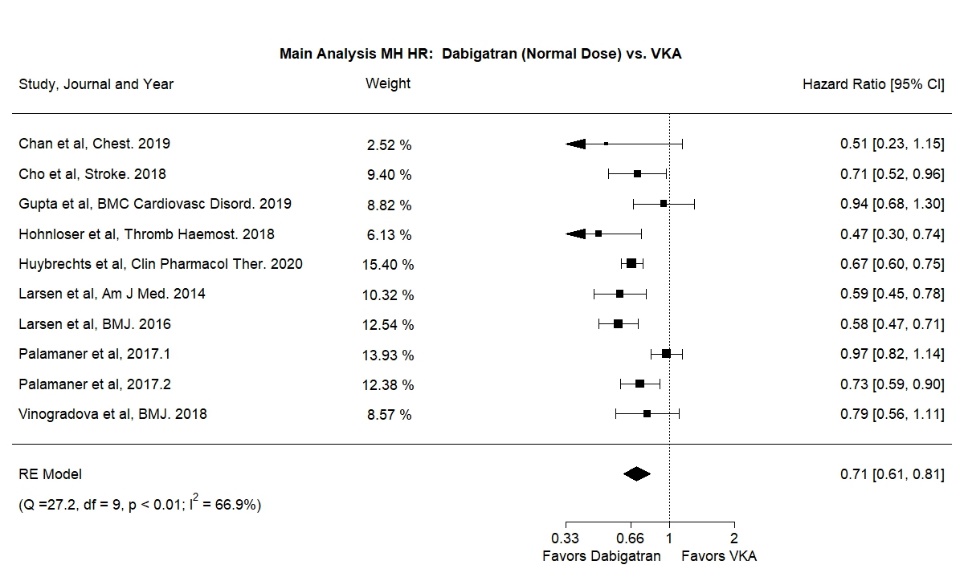


Supplementary Figure 4C Supplementary Figure 4D


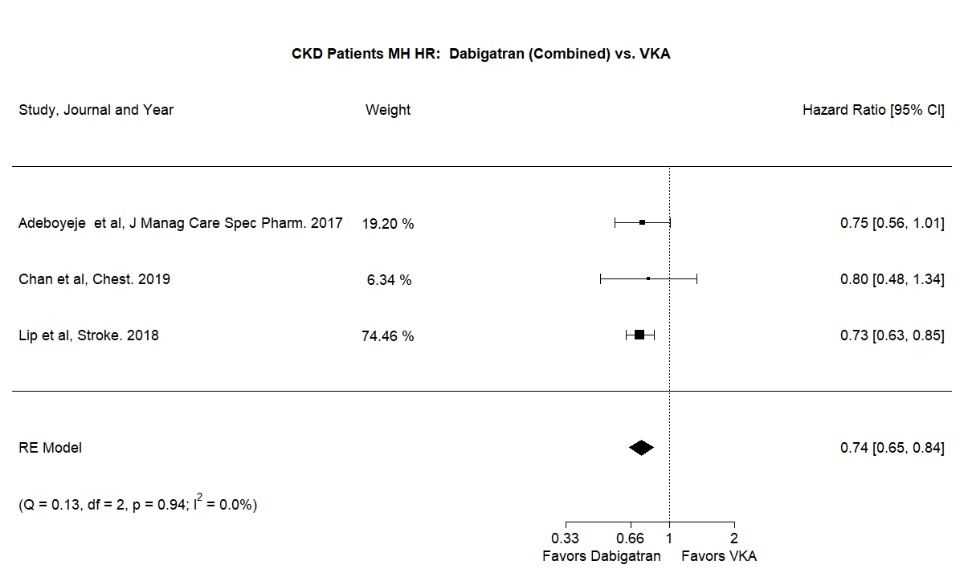

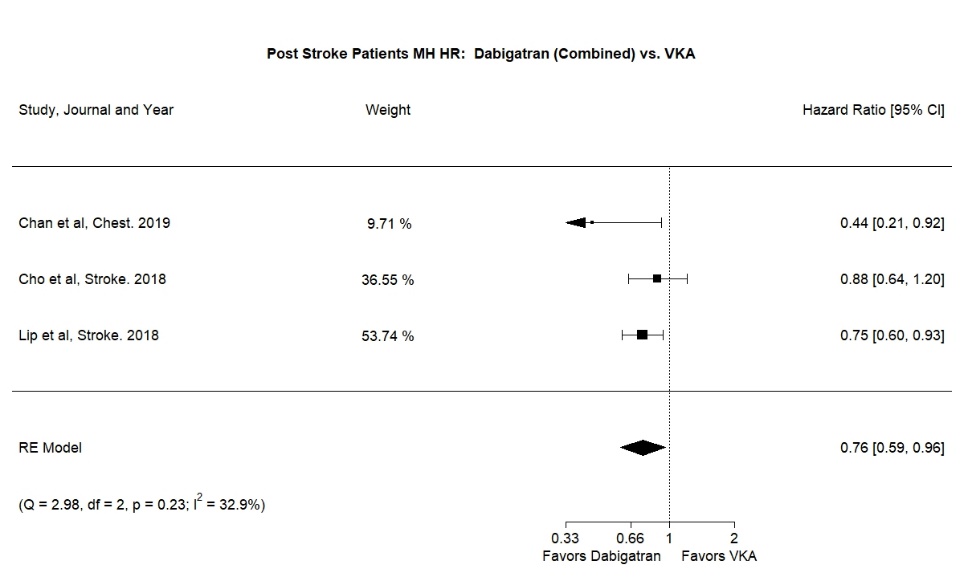


Supplementary Figure 4E Supplementary Figure 4F


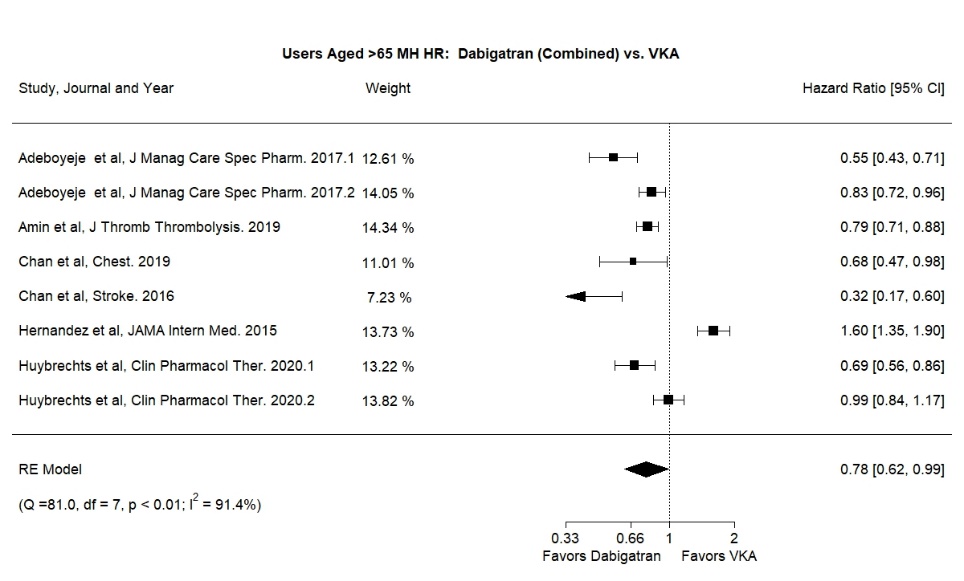

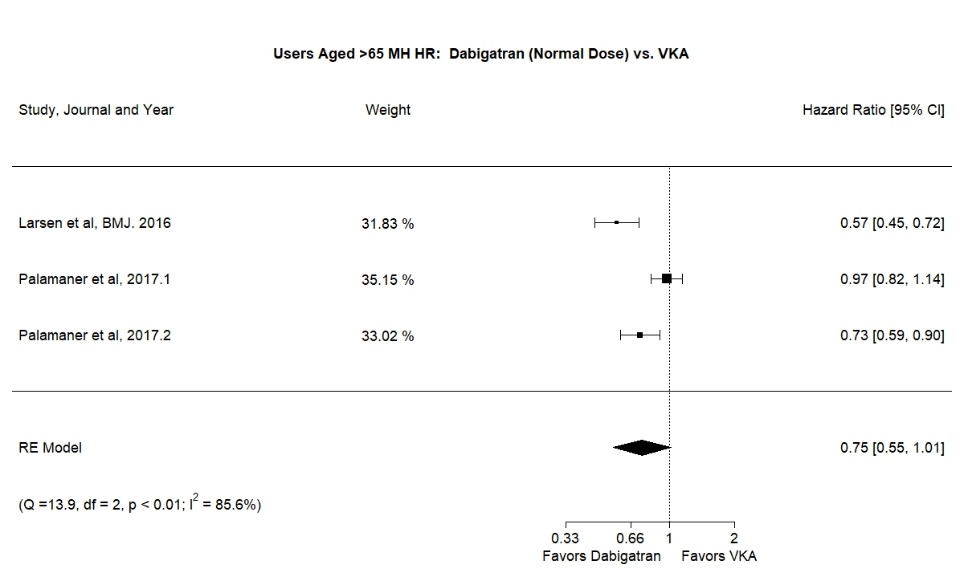


Supplementary Figure 4G Supplementary Figure 4H


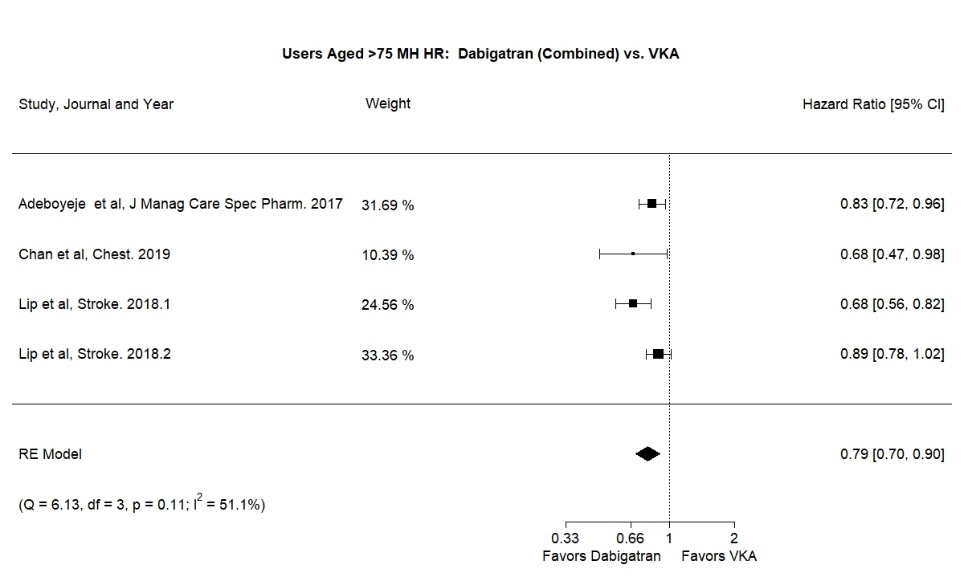

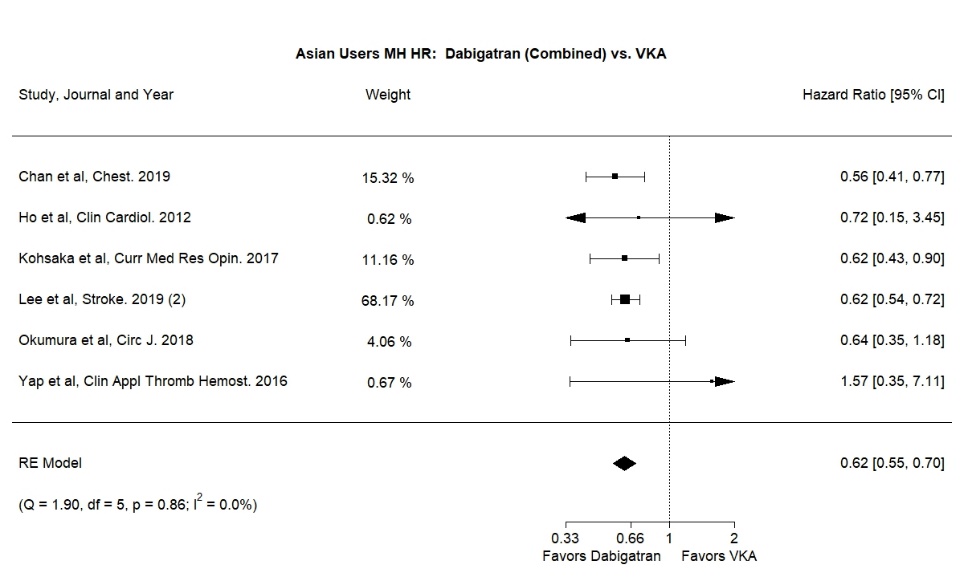


Supplementary Figure 4I Supplementary Figure 4J


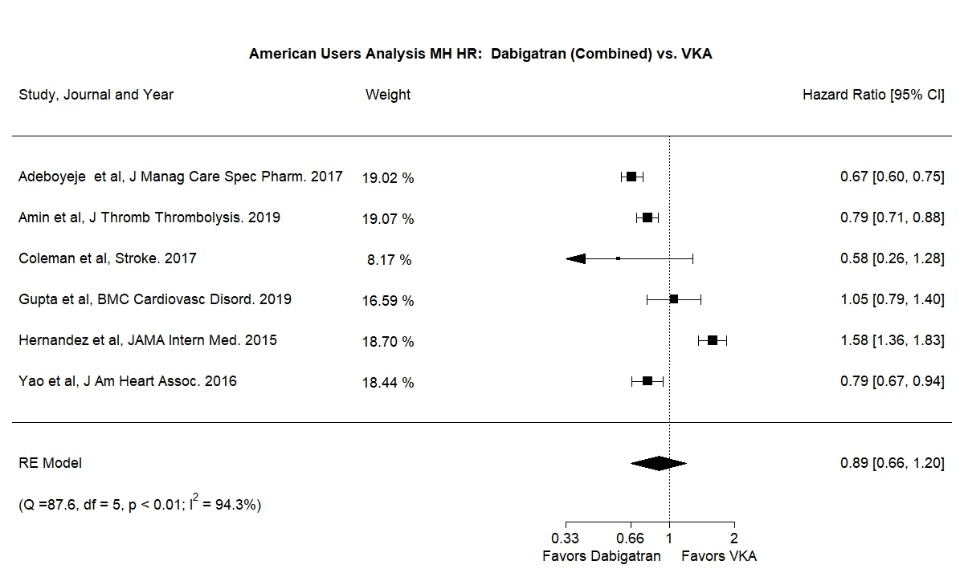

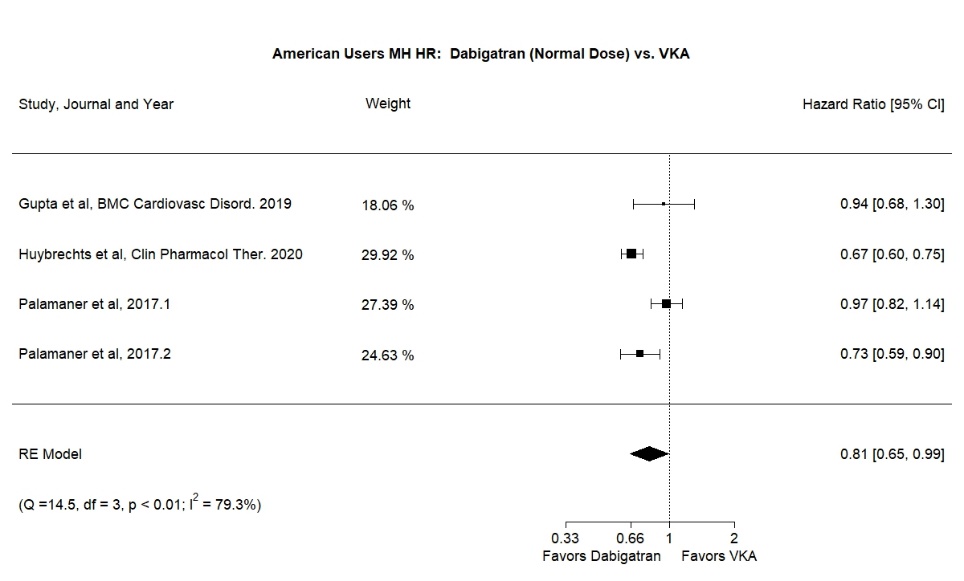


Supplementary Figure 4K Supplementary Figure 4L


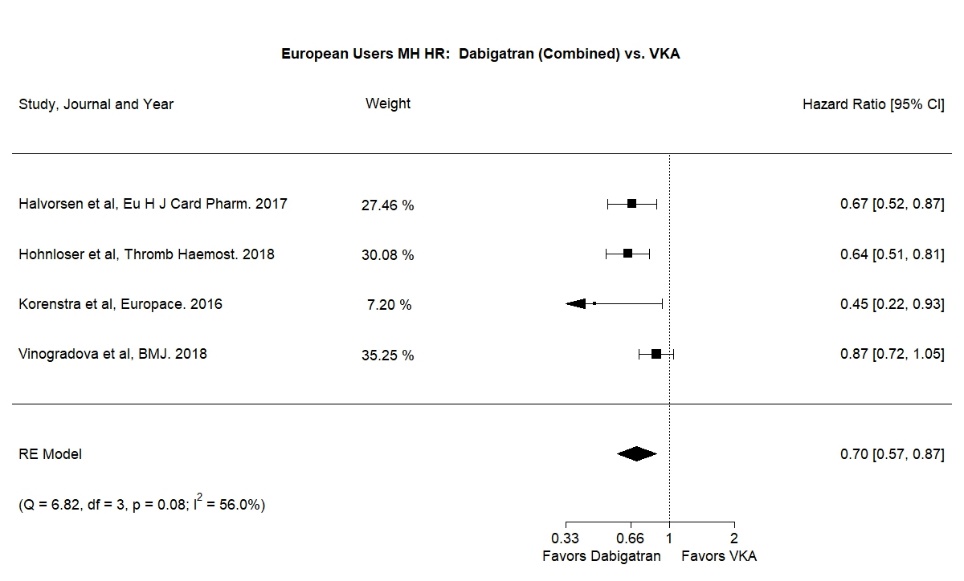

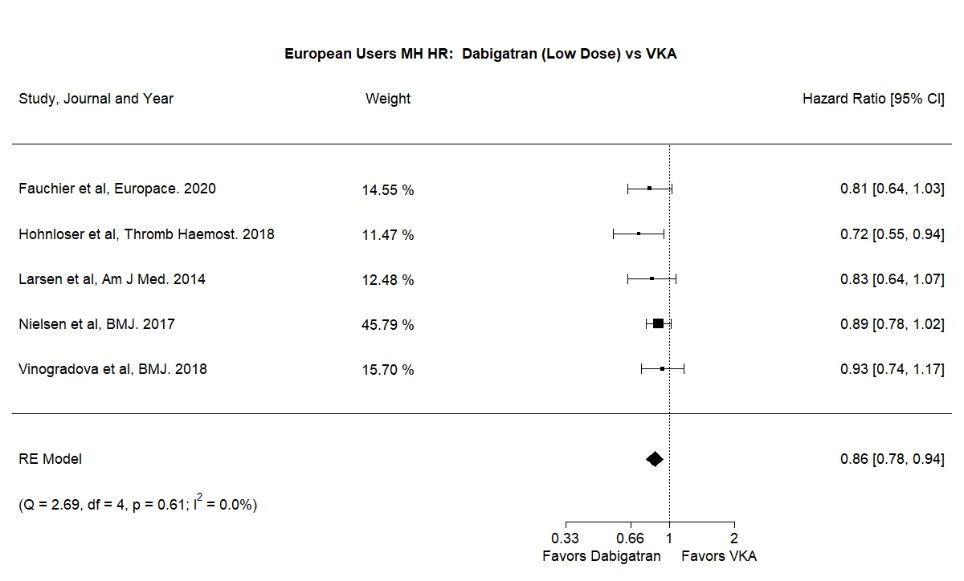


Supplementary Figure 4M

**
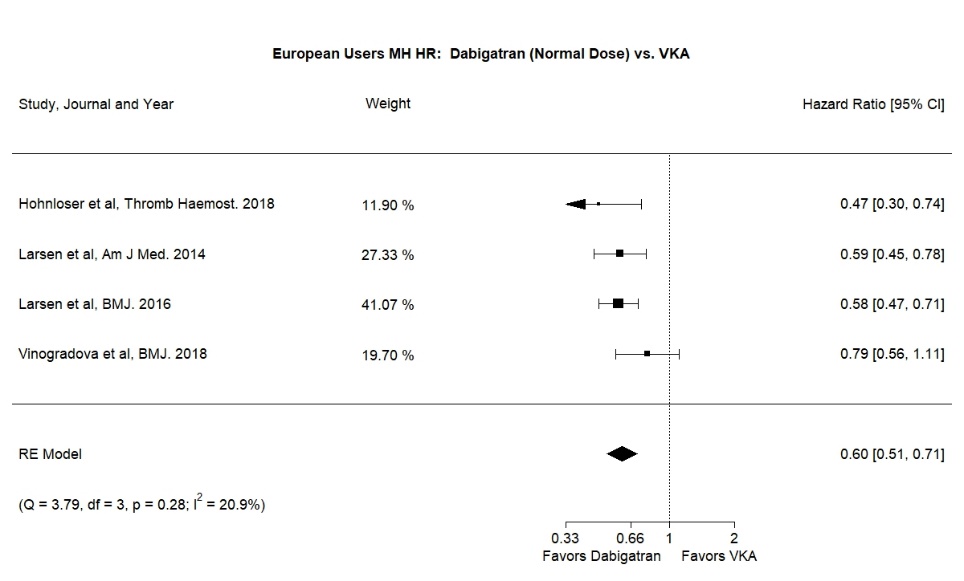
**

**Supplementary Figure 5**

Supplementary Figure 5A Supplementary Figure 5B


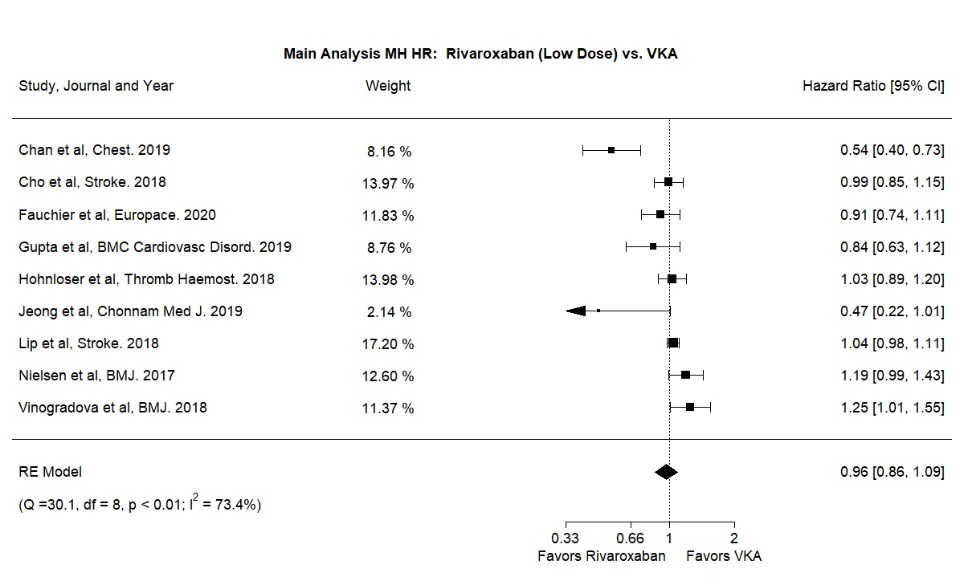

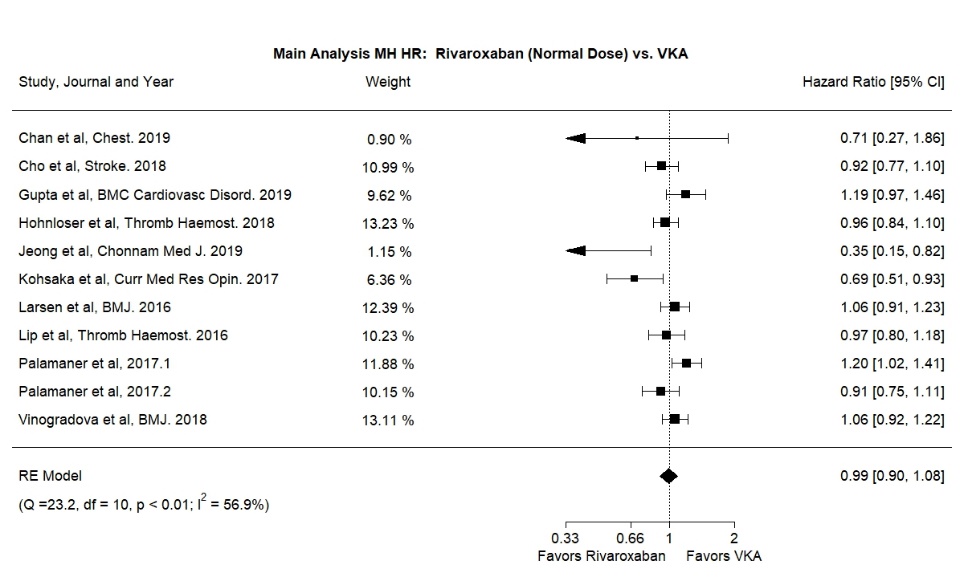


Supplementary Figure 5C Supplementary Figure 5D


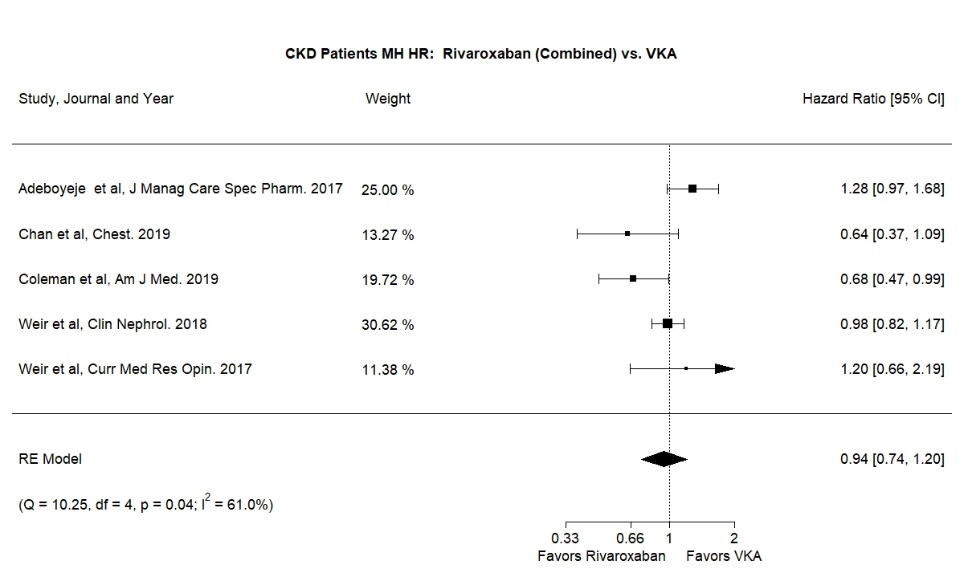

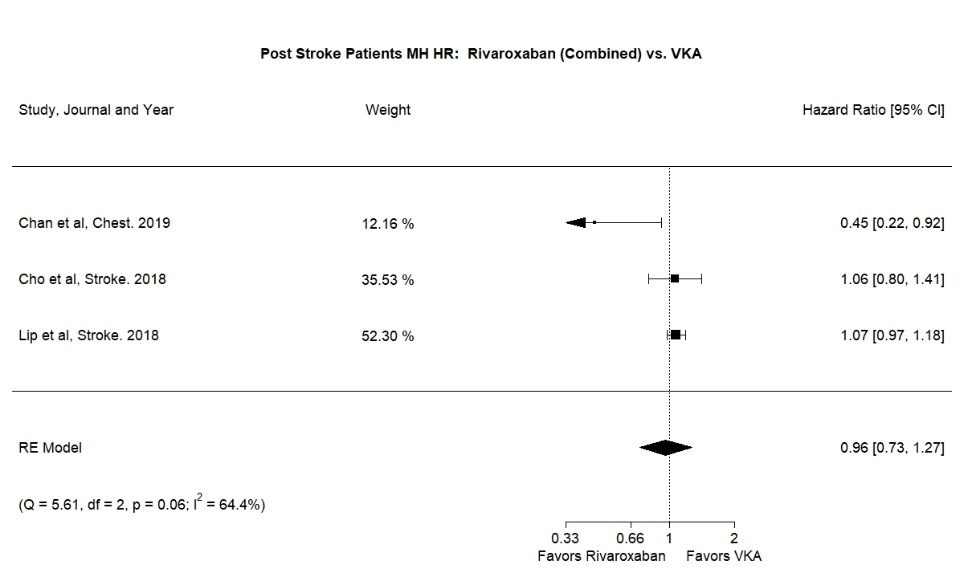


Supplementary Figure 5E Supplementary Figure 5F

**
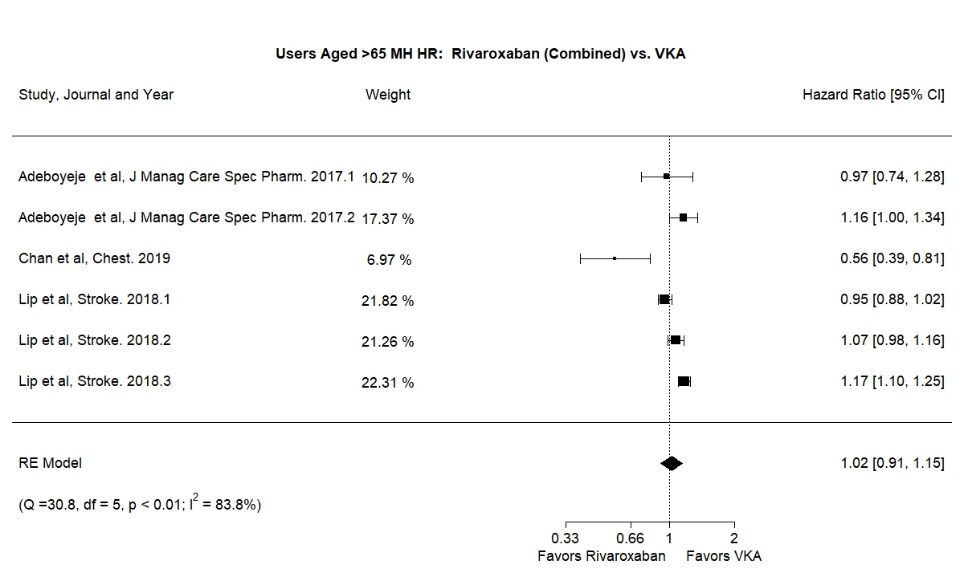

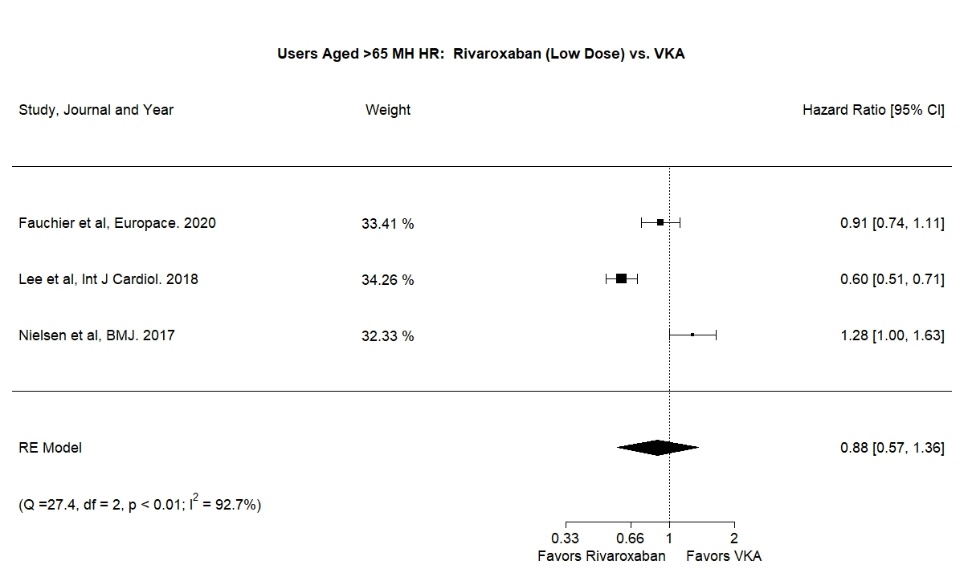
**

Supplementary Figure 5G Supplementary Figure 5H

**
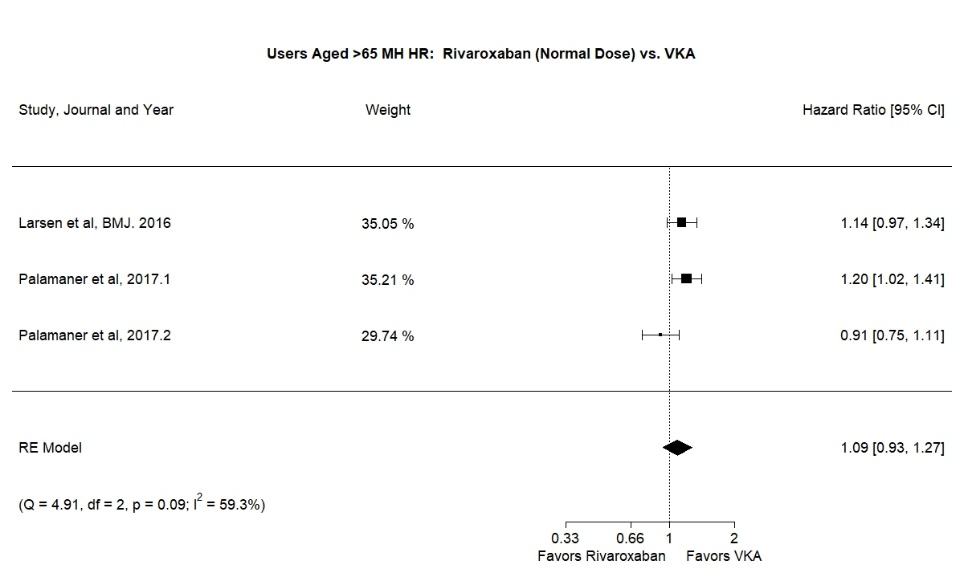

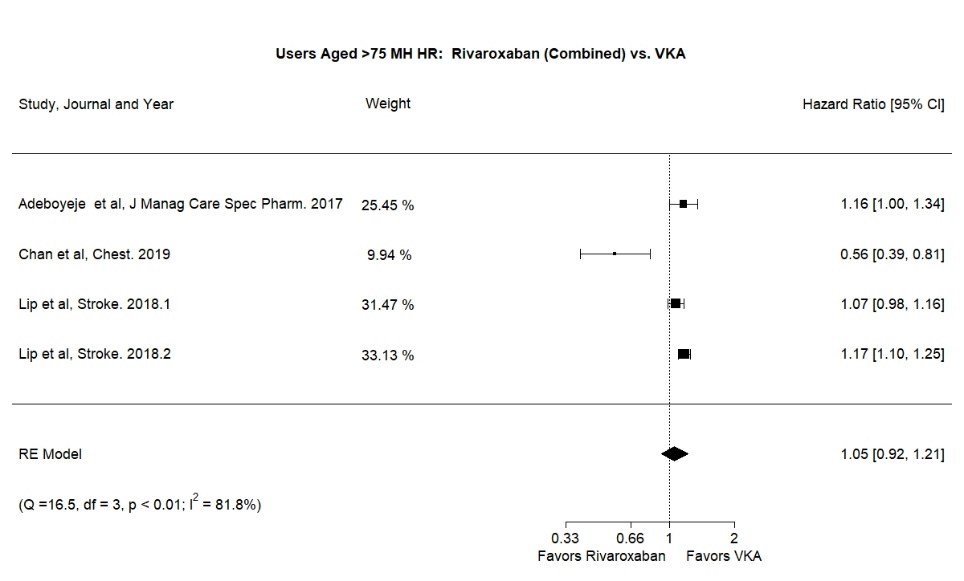
**

Supplementary Figure 5I Supplementary Figure 5J


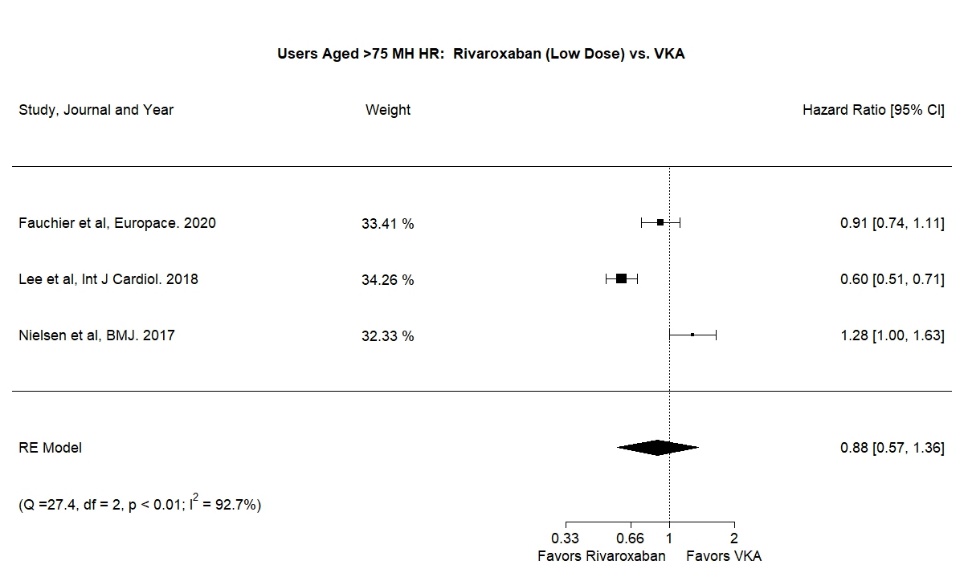

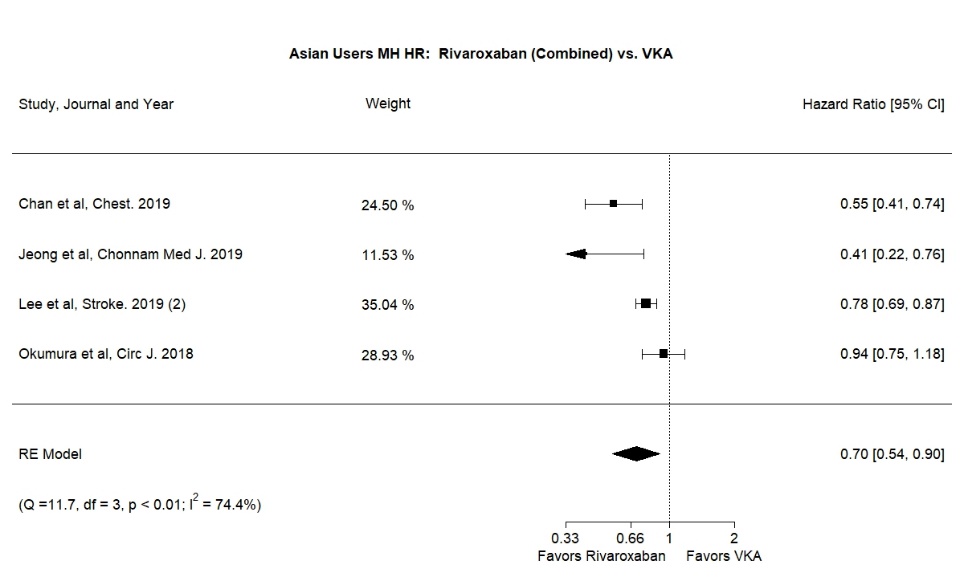


Supplementary Figure 5K Supplementary Figure 5L


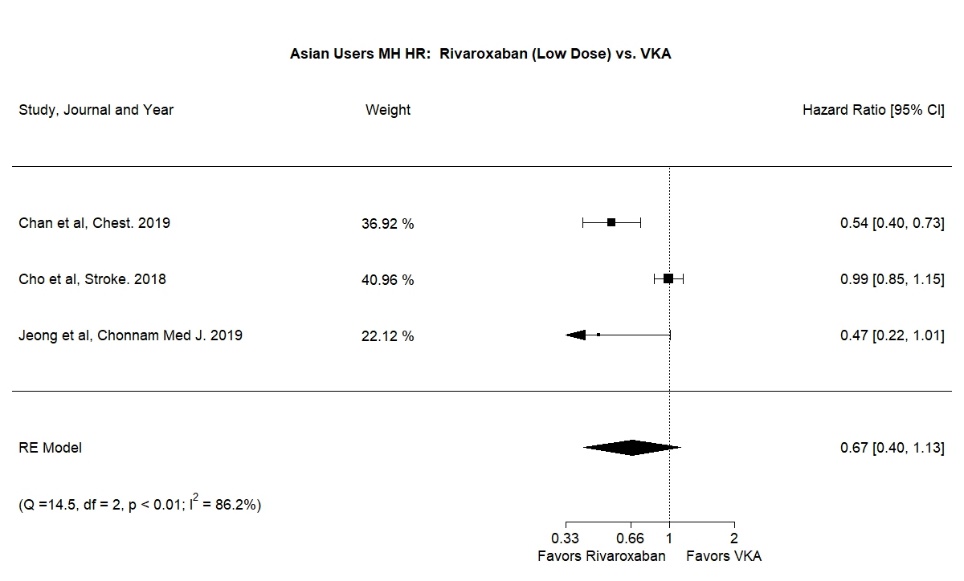

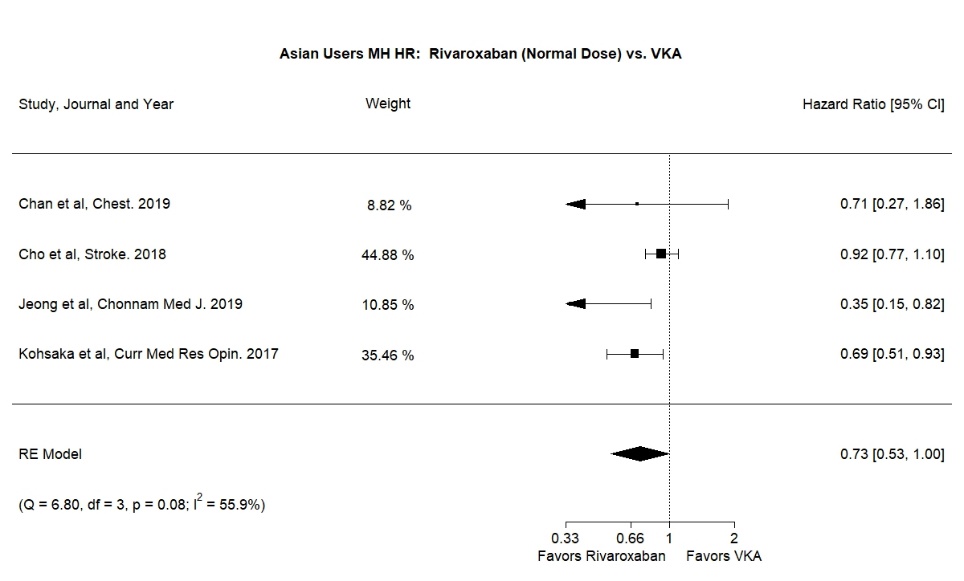


Supplementary Figure 5M Supplementary Figure 5N


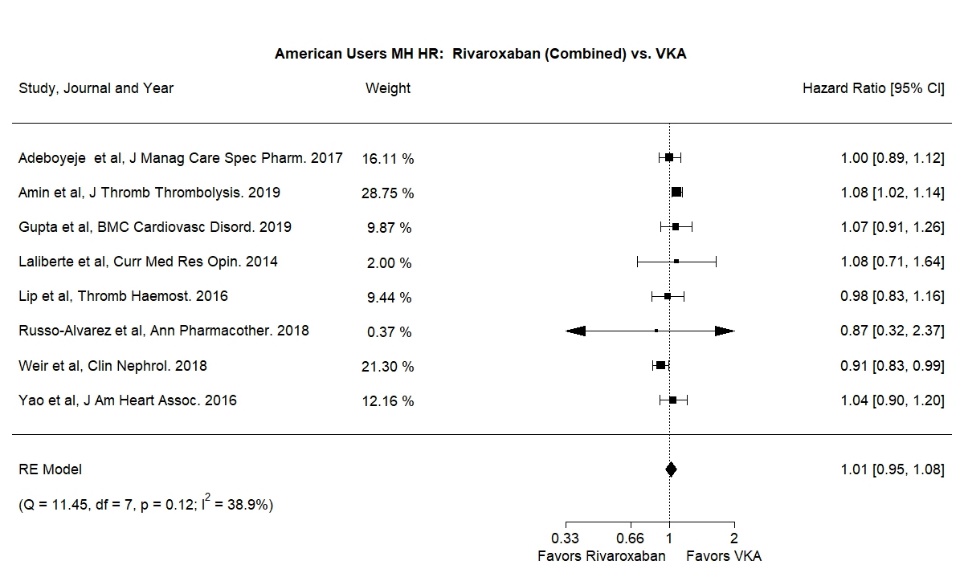

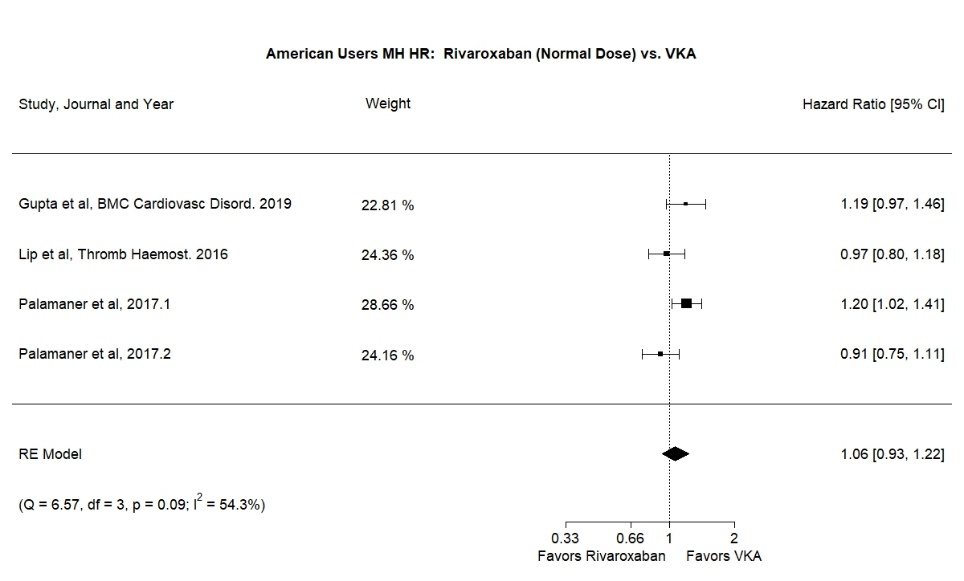


Supplementary Figure 5O Supplementary Figure 5P


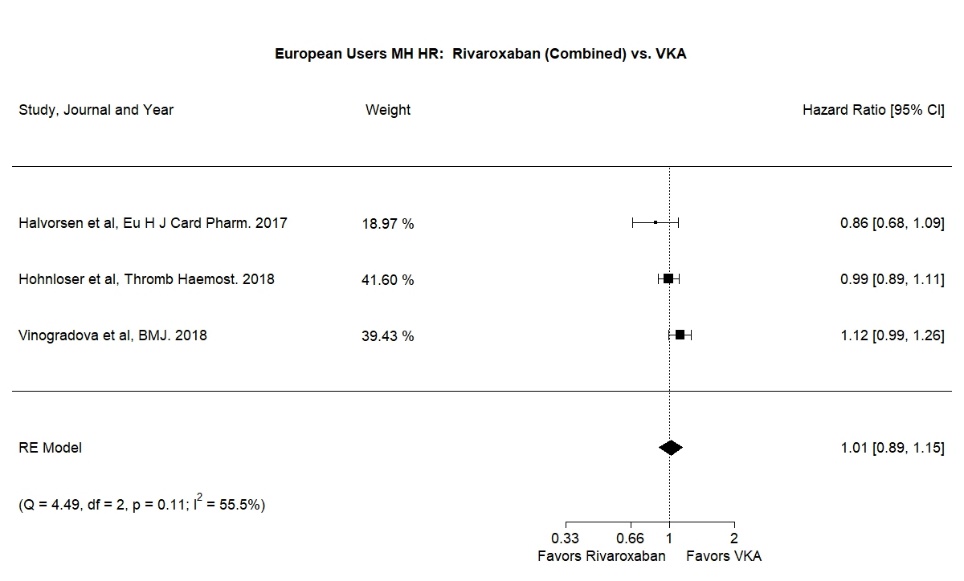

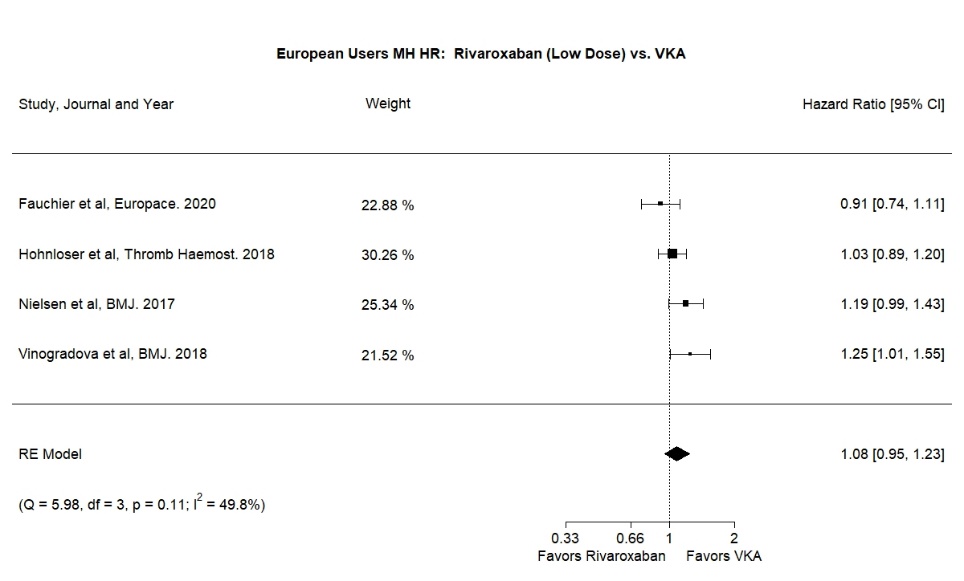


Supplementary Figure 5Q


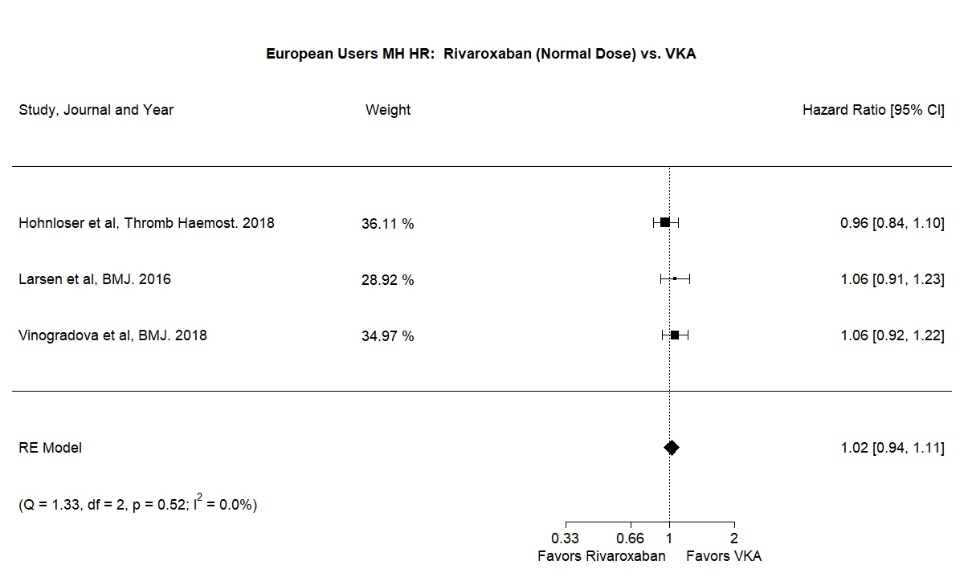


**Supplementary Figure 6**

Supplementary Figure 6A Supplementary Figure 6B


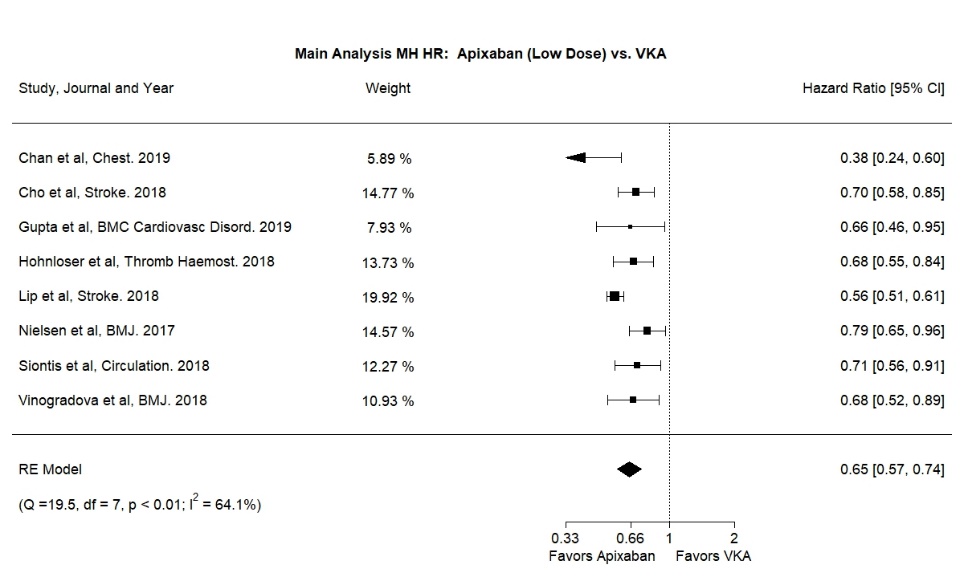

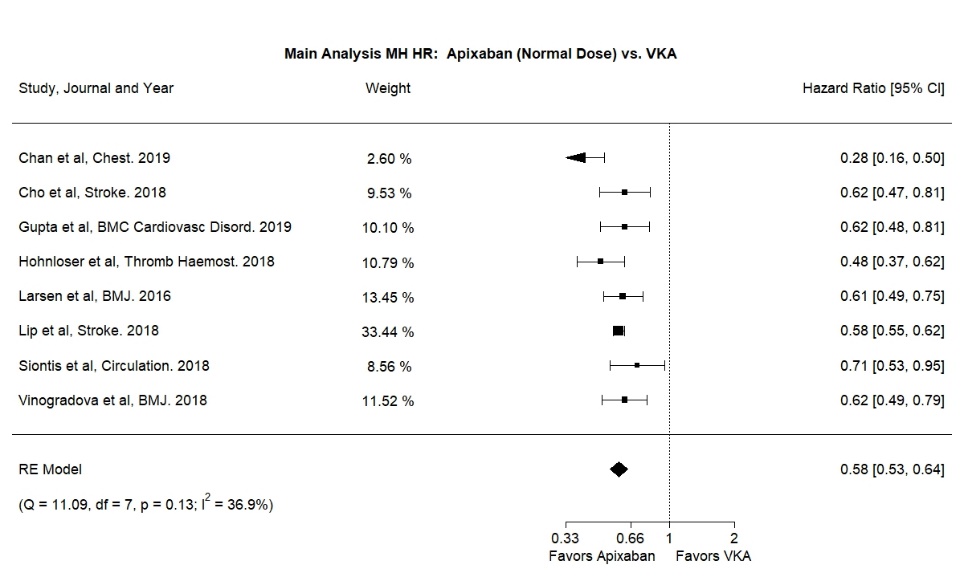


Supplementary Figure 6C Supplementary Figure 6D


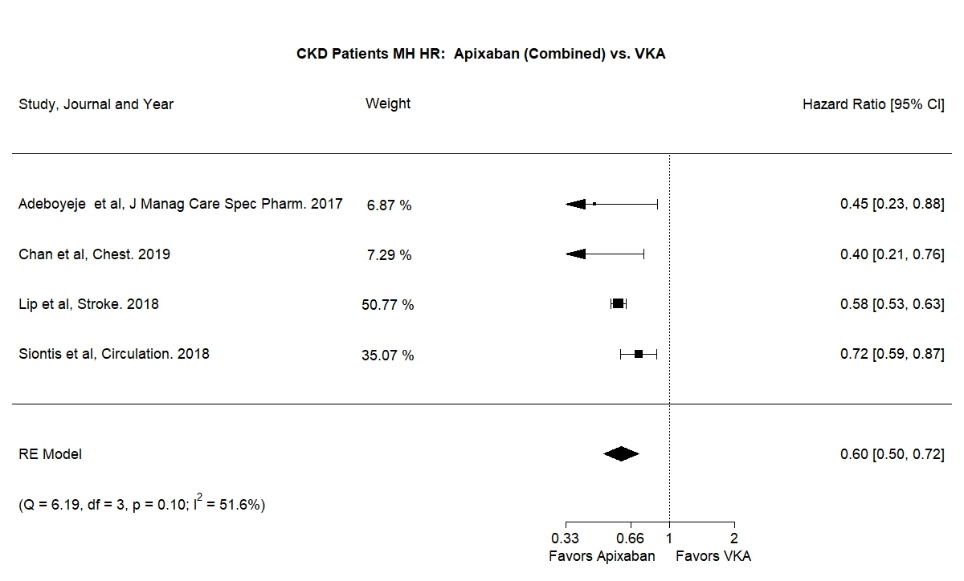

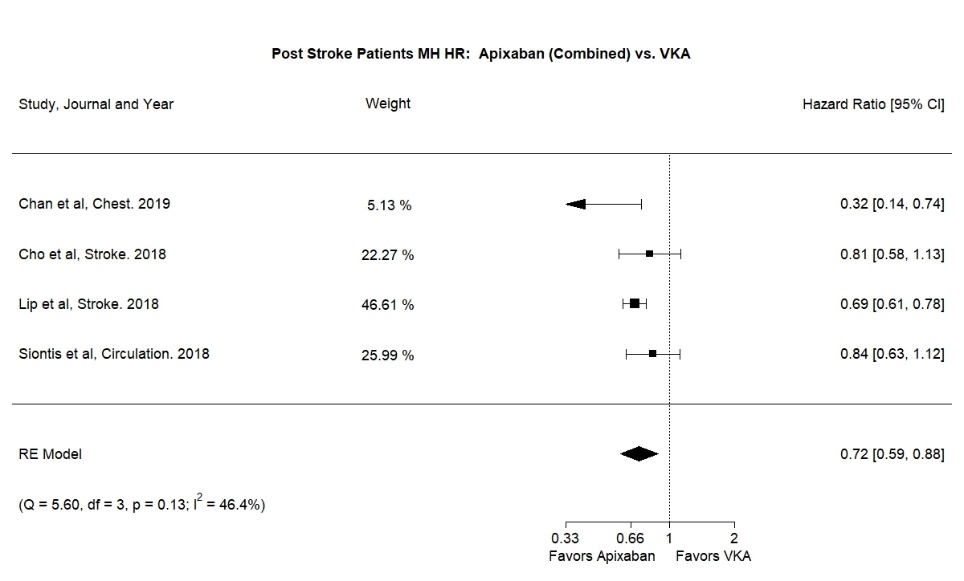


Supplementary Figure 6E Supplementary Figure 6F


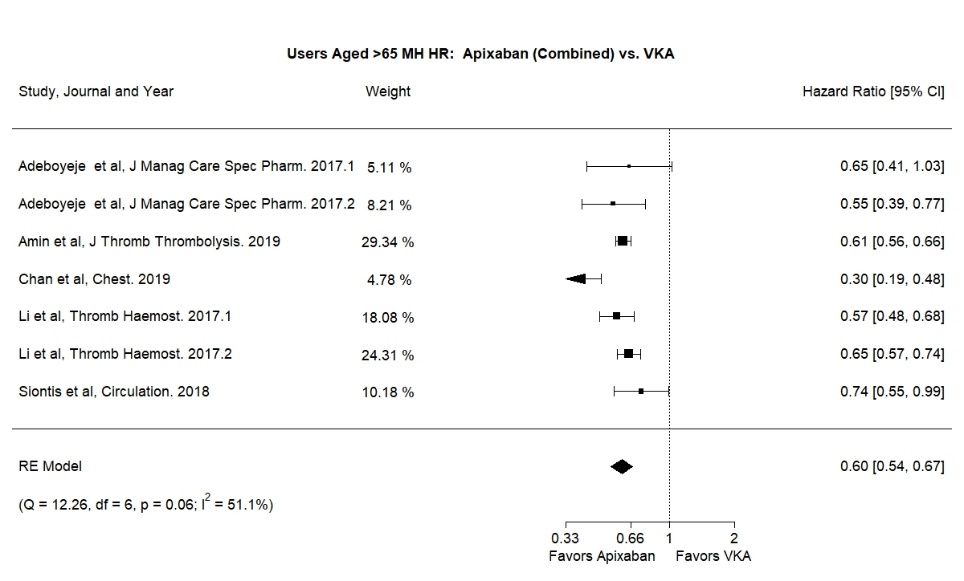

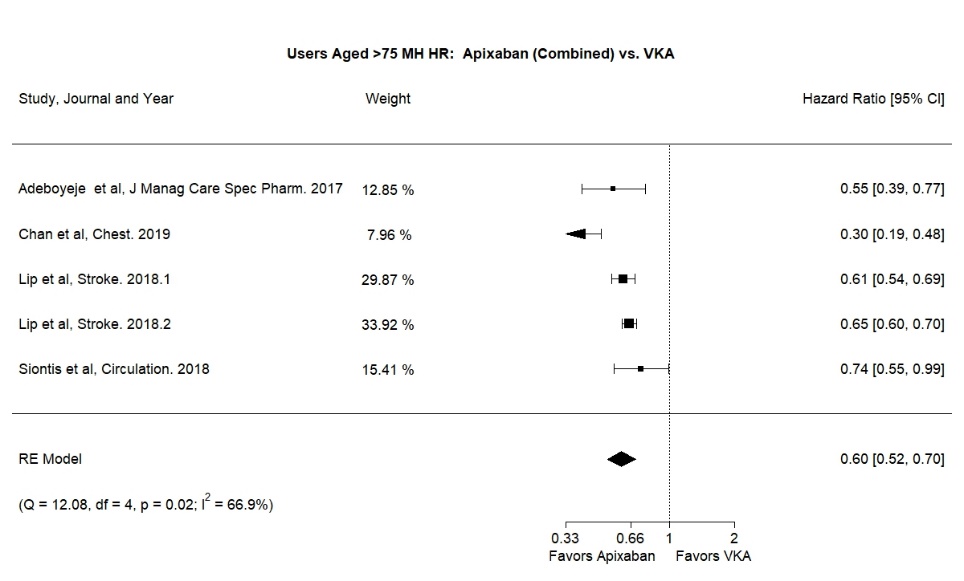


Supplementary Figure 6G Supplementary Figure 6H


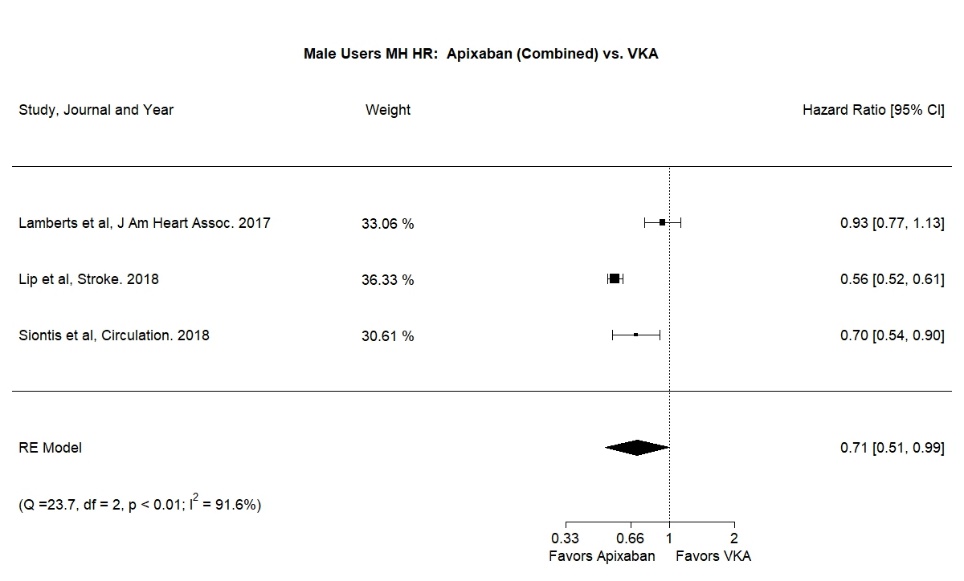

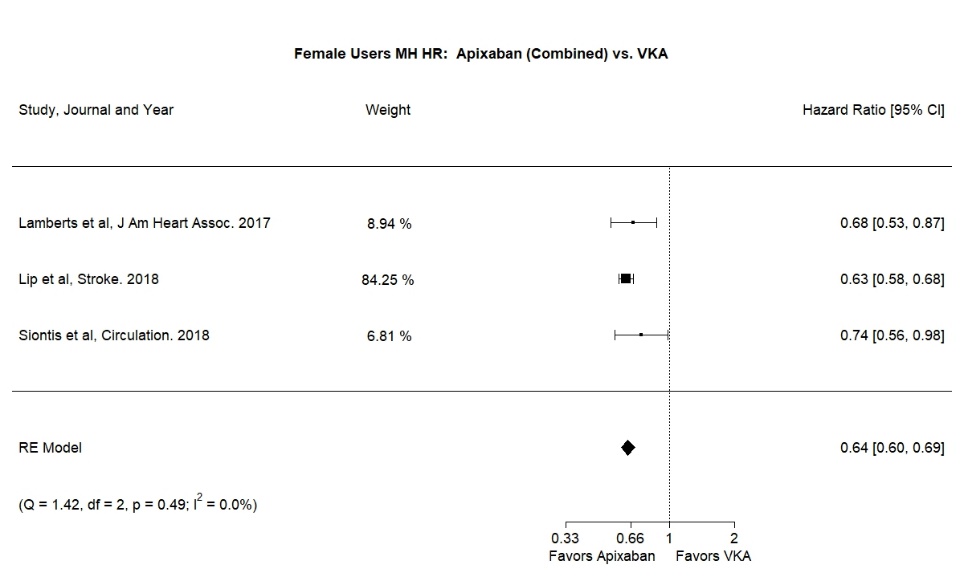


Supplementary Figure 6I Supplementary Figure 6J


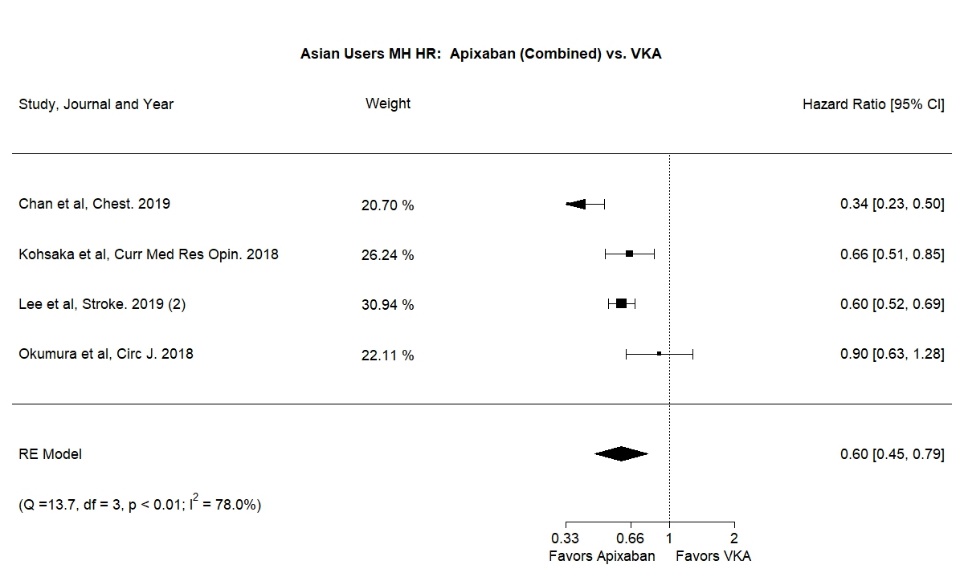

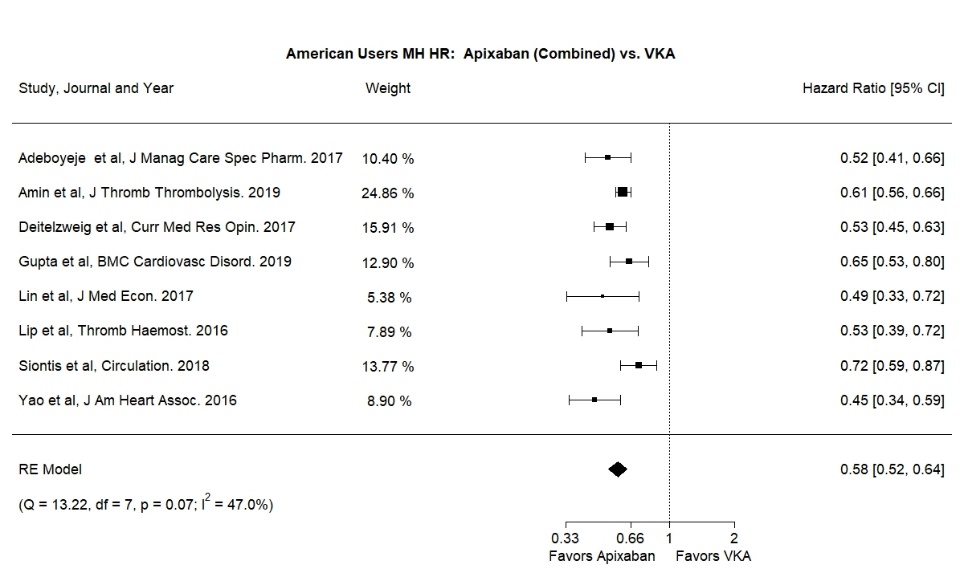


Supplementary Figure 6K Supplementary Figure 6L


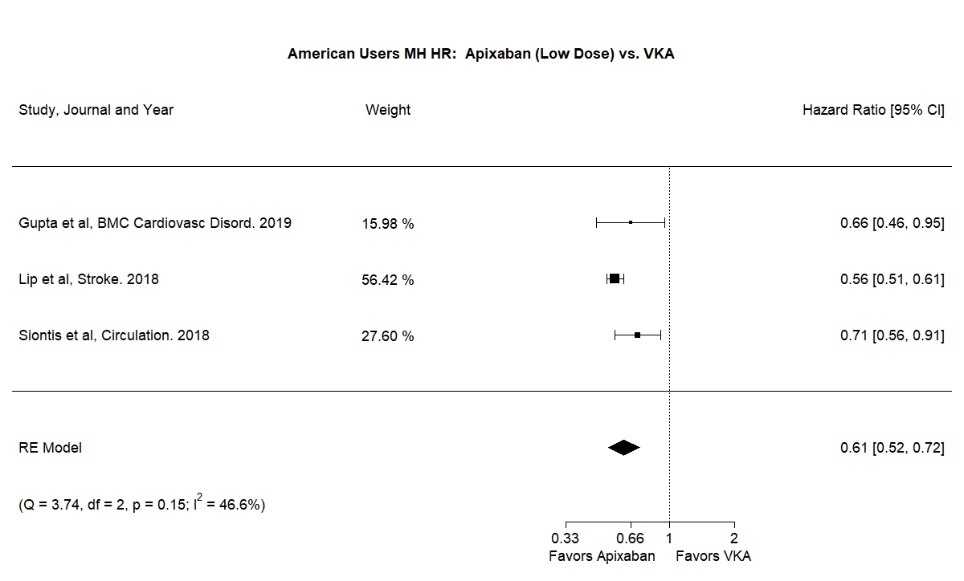

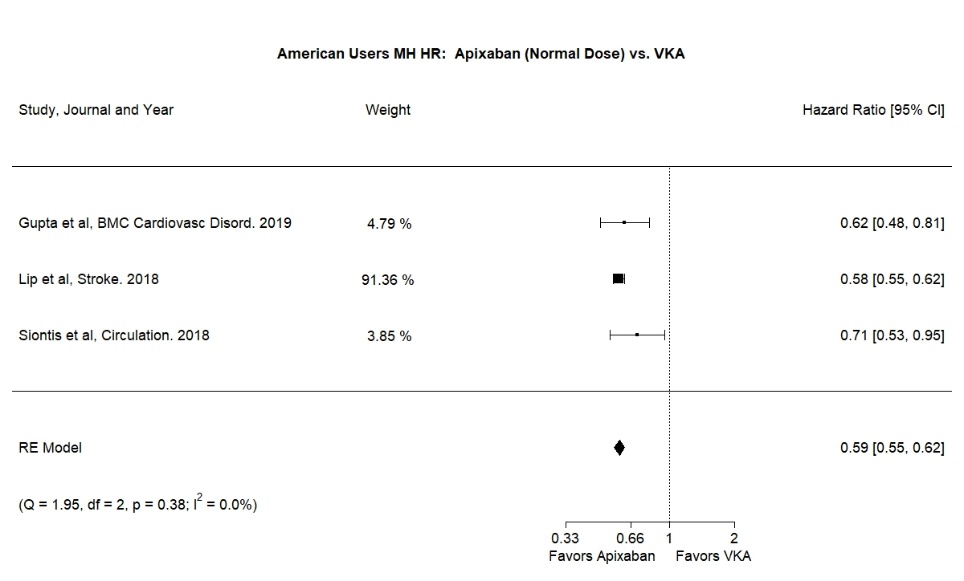


Supplementary Figure 6M Supplementary Figure 6N


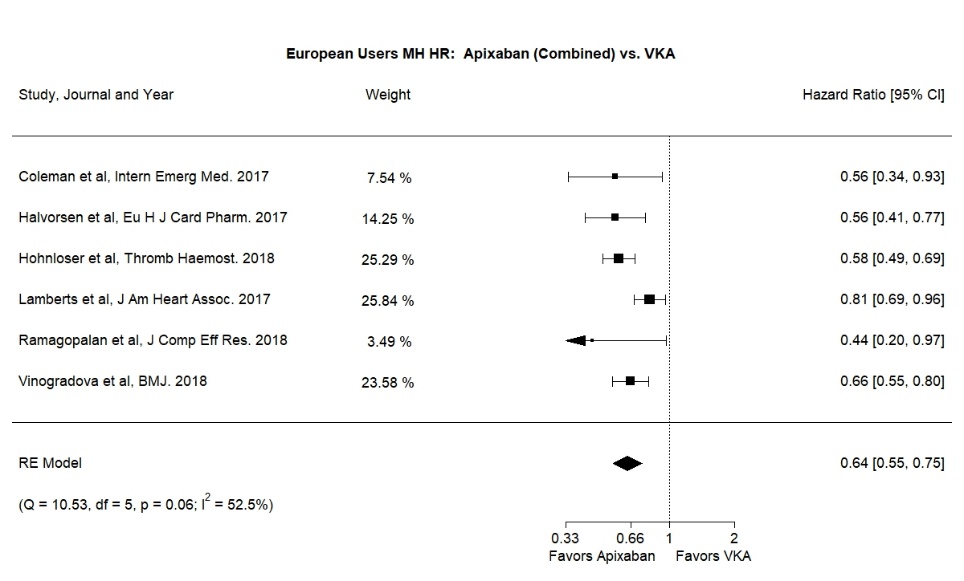

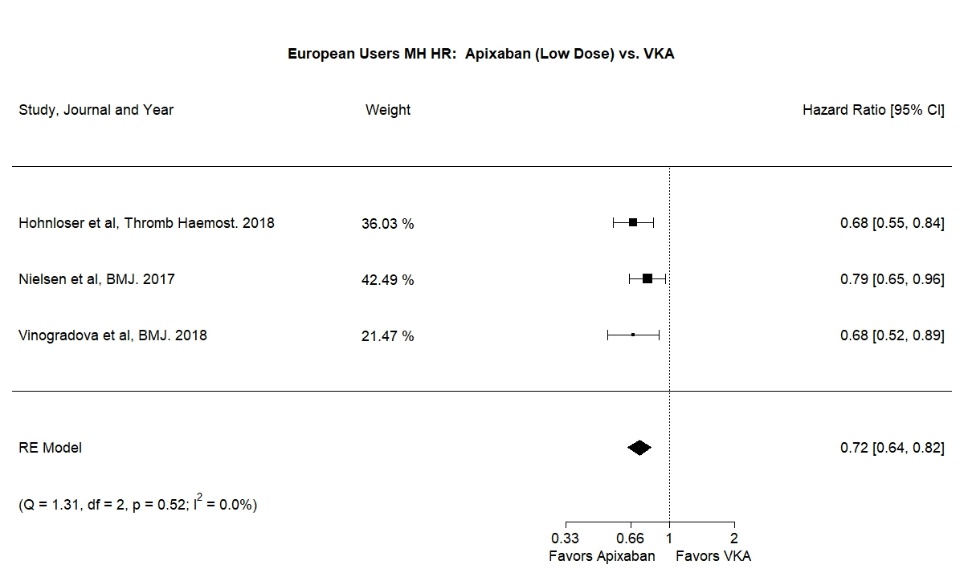


Supplementary Figure 6O


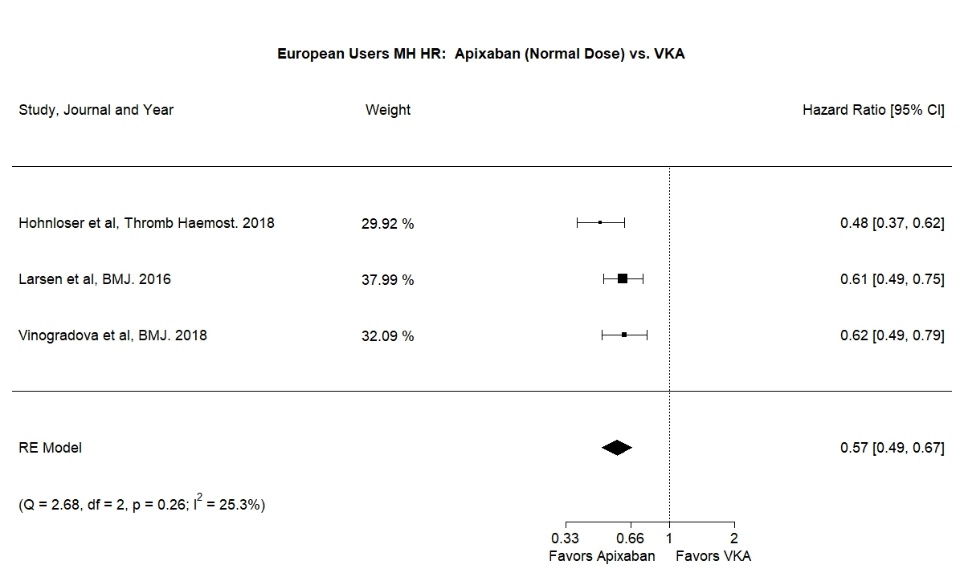


**Supplementary Figure 7**

Supplementary Figure 7A Supplementary Figure 7B


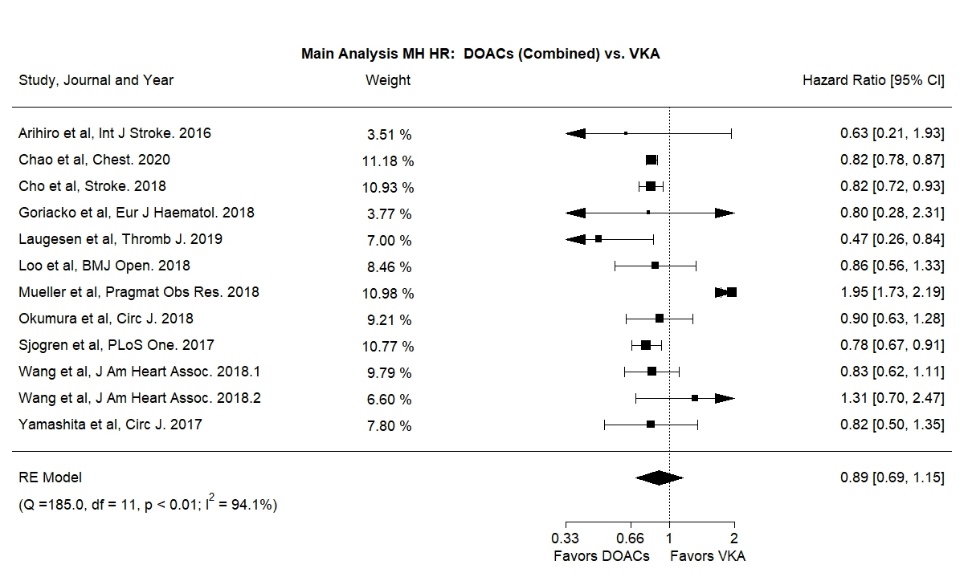

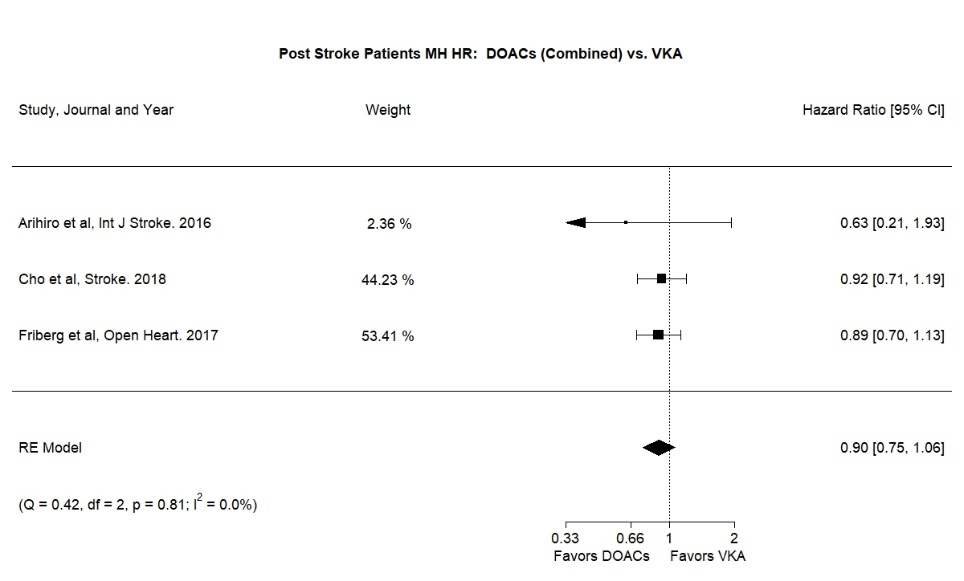


Supplementary Figure 7C Supplementary Figure 7D


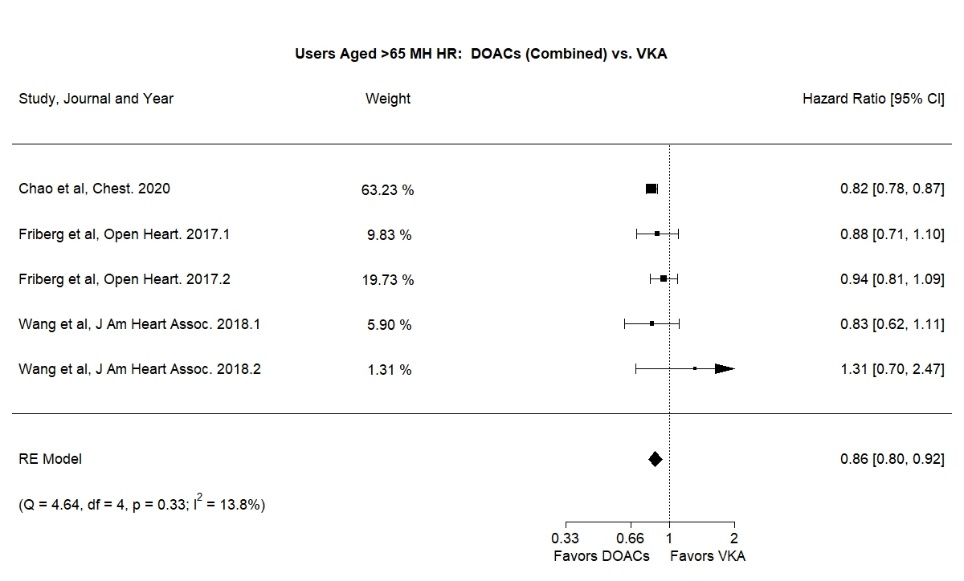

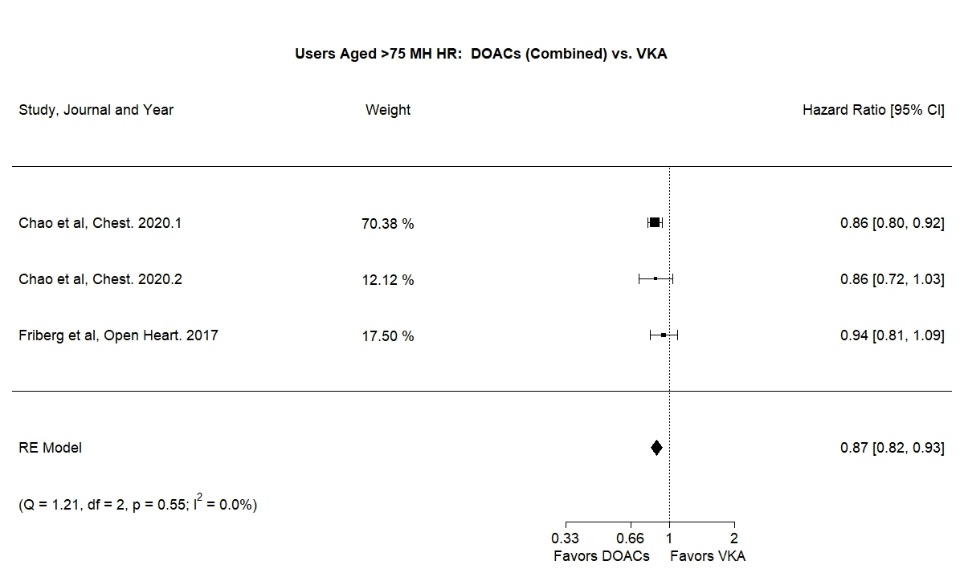


Supplementary Figure 7E Supplementary Figure 7F


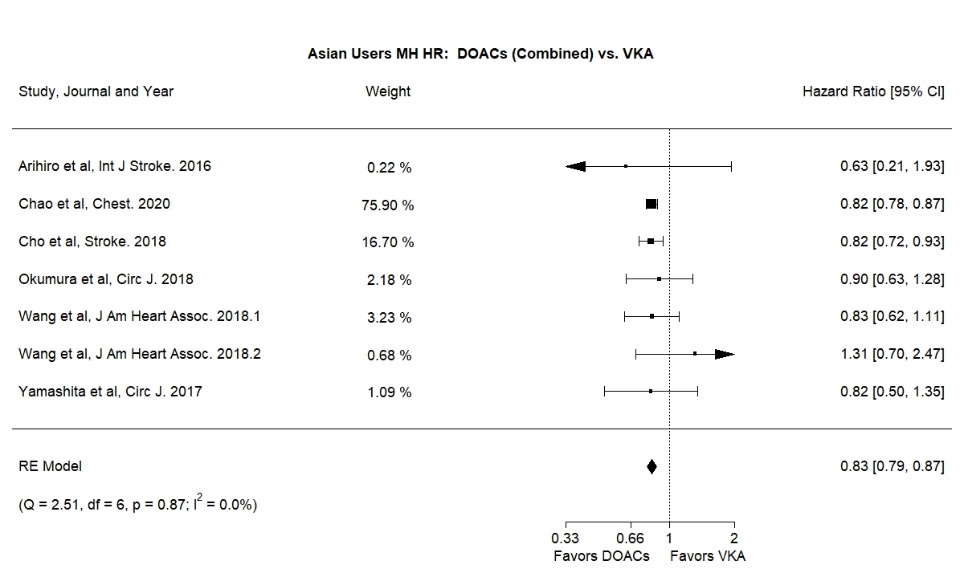

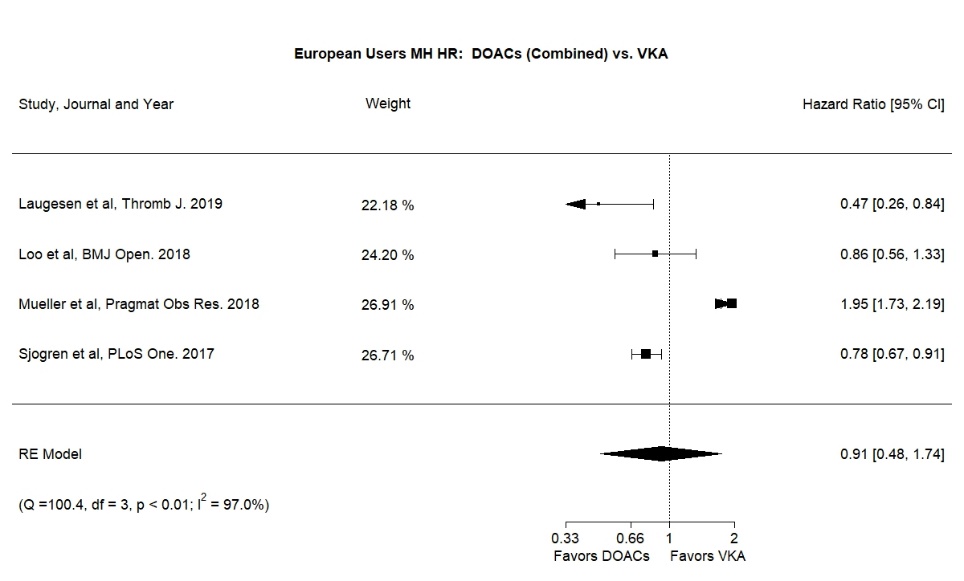


**Supplementary Figure 8**

Supplementary Figure 8A Supplementary Figure 8B


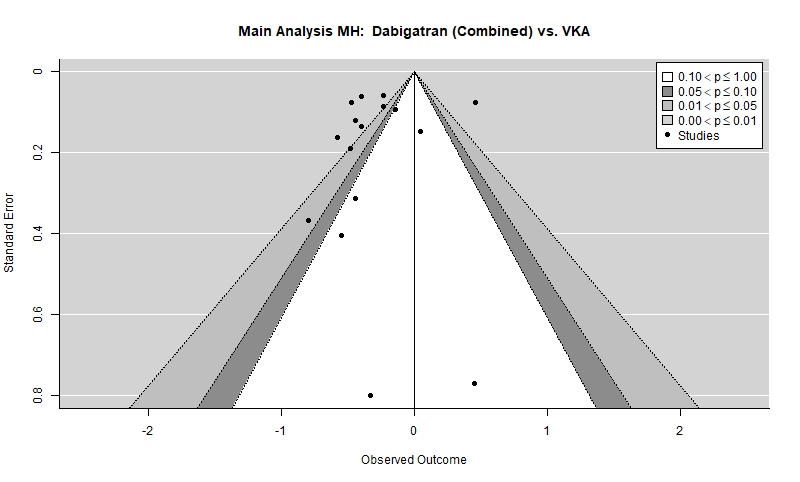

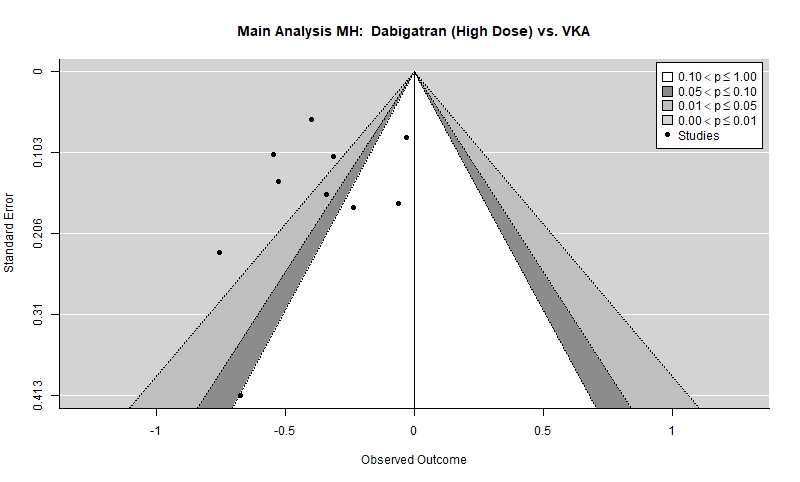


Supplementary Figure 8C Supplementary Figure 8D


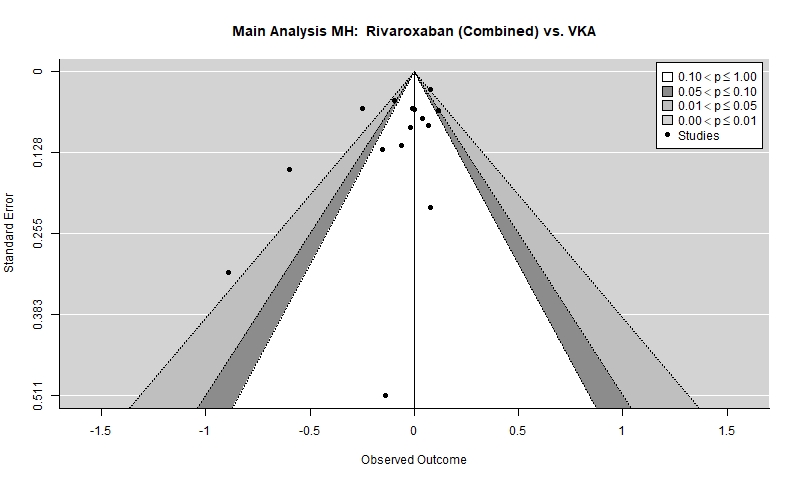

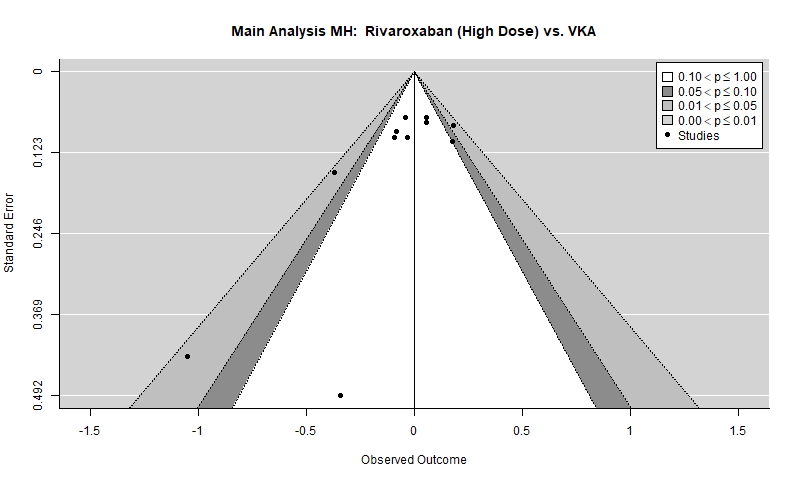


Supplementary Figure 8E Supplementary Figure 8F


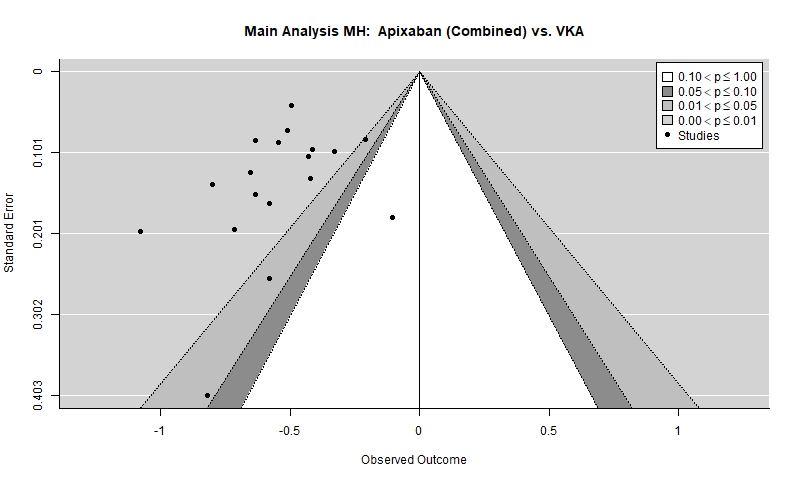

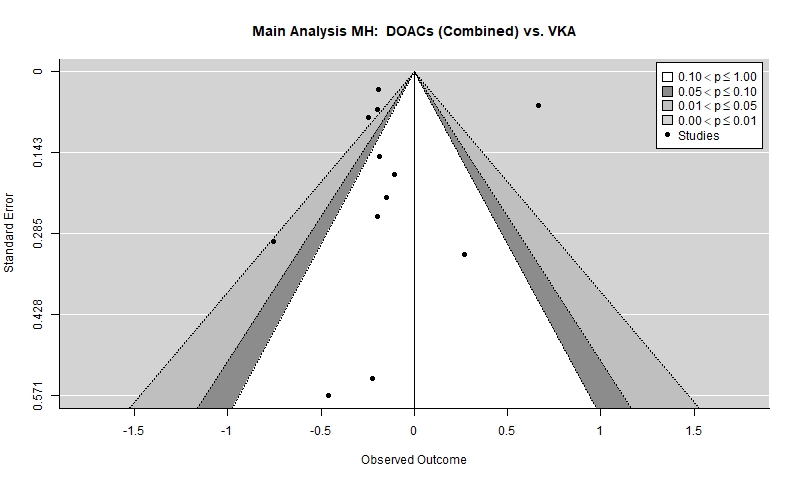


**Supplementary Table 2**

| **Comparison** | **P Value for Egger’s Test** |
| --- | --- |
| Main Analysis Apixaban (Combined) vs Dabigatran | p = 0.0427 |
| Users Aged >65 years old Dabigatran (Combined) vs VKA | p = 0.0191 |
| Users Aged >65 years old Dabigatran (High Dose) vs VKA | p = 0.0002 |
| Main Analysis Rivaroxaban (Combined) vs VKA | p = 0.0240 |
| Main Analysis Rivaroxaban (Low Dose) vs VKA | p = 0.0101 |
| Main Analysis Rivaroxaban (High Dose) vs VKA | p = 0.0067 |
| Users Aged >65 years old Rivaroxaban (Combined) vs VKA | p = 0.0232 |
| Users Aged >65 years old Rivaroxaban (Low Dose) vs VKA | p < .0001 |
| Users Aged >65 years old Rivaroxaban (High Dose) vs VKA | p = 0.0290 |
| Users Aged >75 years old Rivaroxaban (Combined) vs VKA | p = 0.0025 |
| Users Aged >75 years old Rivaroxaban (Low Dose) vs VKA | p < .0001 |

**Supplementary References**

(1-55)

1. Adeboyeje G, Sylwestrzak G, Barron JJ, White J, Rosenberg A, Abarca J, et al. Major Bleeding Risk During Anticoagulation with Warfarin, Dabigatran, Apixaban, or Rivaroxaban in Patients with Nonvalvular Atrial Fibrillation. J Manag Care Spec Pharm. 2017;23(9):968-78.

2. Amin A, Keshishian A, Dina O, Dhamane A, Nadkarni A, Carda E, et al. Comparative clinical outcomes between direct oral anticoagulants and warfarin among elderly patients with non-valvular atrial fibrillation in the CMS medicare population. J Thromb Thrombolysis. 2019;48(2):240-9.

3. Arihiro S, Todo K, Koga M, Furui E, Kinoshita N, Kimura K, et al. Three-month risk-benefit profile of anticoagulation after stroke with atrial fibrillation: The SAMURAI-Nonvalvular Atrial Fibrillation (NVAF) study. International Journal of Stroke. 2016;11(5):565-74.

4. Chan YH, Kuo CT, Yeh YH, Chang SH, Wu LS, Lee HF, et al. Thromboembolic, Bleeding, and Mortality Risks of Rivaroxaban and Dabigatran in Asians With Nonvalvular Atrial Fibrillation. J Am Coll Cardiol. 2016;68(13):1389-401.

5. Chan YH, Lee HF, See LC, Tu HT, Chao TF, Yeh YH, et al. Effectiveness and Safety of Four Direct Oral Anticoagulants in Asian Patients With Nonvalvular Atrial Fibrillation. Chest. 2019;156(3):529-43.

6. Chan YH, Yen KC, See LC, Chang SH, Wu LS, Lee HF, et al. Cardiovascular, Bleeding, and Mortality Risks of Dabigatran in Asians With Nonvalvular Atrial Fibrillation. Stroke. 2016;47(2):441-9.

7. Chao TF, Chiang CE, Liao JN, Chen TJ, Lip GYH, Chen SA. Comparing the Effectiveness and Safety of Nonvitamin K Antagonist Oral Anticoagulants and Warfarin in Elderly Asian Patients With Atrial Fibrillation: A Nationwide Cohort Study. Chest. 2020;157(5):1266-77.

8. Cho MS, Yun JE, Park JJ, Kim YJ, Lee J, Kim H, et al. Outcomes After Use of Standard- and Low-Dose Non-Vitamin K Oral Anticoagulants in Asian Patients With Atrial Fibrillation. Stroke. 2018:STROKEAHA118023093.

9. Coleman CI, Antz M. Real-world evidence with apixaban for stroke prevention in patients with nonvalvular atrial fibrillation in Germany: a retrospective study (REASSESS). Intern Emerg Med. 2017;12(3):419-22.

10. Coleman CI, Kreutz R, Sood NA, Bunz TJ, Eriksson D, Meinecke AK, et al. Rivaroxaban Versus Warfarin in Patients With Nonvalvular Atrial Fibrillation and Severe Kidney Disease or Undergoing Hemodialysis. Am J Med. 2019;132(9):1078-83.

11. Coleman CI, Peacock WF, Bunz TJ, Alberts MJ. Effectiveness and Safety of Apixaban, Dabigatran, and Rivaroxaban Versus Warfarin in Patients With Nonvalvular Atrial Fibrillation and Previous Stroke or Transient Ischemic Attack. Stroke. 2017;48(8):2142-9.

12. Deitelzweig S, Luo X, Gupta K, Trocio J, Mardekian J, Curtice T, et al. Comparison of effectiveness and safety of treatment with apixaban vs. other oral anticoagulants among elderly nonvalvular atrial fibrillation patients. Curr Med Res Opin. 2017;33(10):1745-54.

13. Fauchier L, Blin P, Sacher F, Dureau-Pournin C, Bernard MA, Lassalle R, et al. Reduced dose of rivaroxaban and dabigatran vs. vitamin K antagonists in very elderly patients with atrial fibrillation in a nationwide cohort study. Europace. 2020;22(2):205-15.

14. Friberg L, Oldgren J. Efficacy and safety of non-vitamin K antagonist oral anticoagulants compared with warfarin in patients with atrial fibrillation. Open Heart. 2017;4(2).

15. Goriacko PV, K. T. Safety of direct oral anticoagulants vs warfarin in patients with chronic liver disease and atrial fibrillation. Eur J Haematol. 2018;100(5):488-93.

16. Gupta K, Trocio J, Keshishian A, Zhang Q, Dina O, Mardekian J, et al. Effectiveness and safety of direct oral anticoagulants compared to warfarin in treatment naive non-valvular atrial fibrillation patients in the US Department of defense population. BMC Cardiovasc Disord. 2019;19(1):142.

17. Gupta K, Trocio J, Keshishian A, Zhang Q, Dina O, Mardekian J, et al. Real-World Comparative Effectiveness, Safety, and Health Care Costs of Oral Anticoagulants in Nonvalvular Atrial Fibrillation Patients in the U.S. Department of Defense Population. J Manag Care Spec Pharm. 2018;24(11):1116-27.

18. Halvorsen S, Ghanima W, Fride Tvete I, Hoxmark C, Falck P, Solli O, et al. A nationwide registry study to compare bleeding rates in patients with atrial fibrillation being prescribed oral anticoagulants. Eur Heart J Cardiovasc Pharmacother. 2017;3(1):28-36.

19. Hernandez I, Baik SH, Piñera A, Zhang Y. Risk of Bleeding With Dabigatran in Atrial Fibrillation. JAMA Internal Medicine. 2015;175(1).

20. Hernandez I, Zhang Y. Comparing Stroke and Bleeding with Rivaroxaban and Dabigatran in Atrial Fibrillation: Analysis of the US Medicare Part D Data. American Journal of Cardiovascular Drugs. 2016;17(1):37-47.

21. Ho JC, Chang AM, Yan BP, Yu CM, Lam YY, Lee VW. Dabigatran compared with warfarin for stroke prevention with atrial fibrillation: experience in Hong Kong. Clin Cardiol. 2012;35(12):E40-5.

22. Hohnloser SH, Basic E, Hohmann C, Nabauer M. Effectiveness and Safety of Non-Vitamin K Oral Anticoagulants in Comparison to Phenprocoumon: Data from 61,000 Patients with Atrial Fibrillation. Thromb Haemost. 2018;118(3):526-38.

23. Huybrechts KFG, C.: Bartels, D. B.: Zint, K.: Gurusamy, V. K.: Landon, J.: Schneeweiss, S. Safety and Effectiveness of Dabigatran and Other Direct Oral Anticoagulants Compared With Warfarin in Patients With Atrial Fibrillation. Clin Pharmacol Ther. 2020;107(6):1405-19.

24. Jeong HK, Lee KH, Park HW, Yoon NS, Kim MC, Lee N, et al. Real World Comparison of Rivaroxaban and Warfarin in Korean Patients with Atrial Fibrillation: Propensity Matching Cohort Analysis. Chonnam Med J. 2019;55(1).

25. Kohsaka S, Katada J, Saito K, Terayama Y. Safety and effectiveness of apixaban in comparison to warfarin in patients with nonvalvular atrial fibrillation: a propensity-matched analysis from Japanese administrative claims data. Curr Med Res Opin. 2018;34(9):1627-34.

26. Kohsaka S, Murata T, Izumi N, Katada J, Wang F, Terayama Y. Bleeding risk of apixaban, dabigatran, and low-dose rivaroxaban compared with warfarin in Japanese patients with non-valvular atrial fibrillation: a propensity matched analysis of administrative claims data. Curr Med Res Opin. 2017;33(11):1955-63.

27. Korenstra J, Wijtvliet EP, Veeger NJ, Geluk CA, Bartels GL, Posma JL, et al. Effectiveness and safety of dabigatran versus acenocoumarol in 'real-world' patients with atrial fibrillation. Europace. 2016;18(9):1319-27.

28. Laliberte F, Cloutier M, Nelson WW, Coleman CI, Pilon D, Olson WH, et al. Real-world comparative effectiveness and safety of rivaroxaban and warfarin in nonvalvular atrial fibrillation patients. Curr Med Res Opin. 2014;30(7):1317-25.

29. Lamberts M, Staerk L, Olesen JB, Fosbol EL, Hansen ML, Harboe L, et al. Major Bleeding Complications and Persistence With Oral Anticoagulation in Non-Valvular Atrial Fibrillation: Contemporary Findings in Real-Life Danish Patients. J Am Heart Assoc. 2017;6(2).

30. Larsen TB, Gorst-Rasmussen A, Rasmussen LH, Skjoth F, Rosenzweig M, Lip GY. Bleeding events among new starters and switchers to dabigatran compared with warfarin in atrial fibrillation. Am J Med. 2014;127(7):650-6 e5.

31. Larsen TB, Skjoth F, Nielsen PB, Kjaeldgaard JN, Lip GY. Comparative effectiveness and safety of non-vitamin K antagonist oral anticoagulants and warfarin in patients with atrial fibrillation: propensity weighted nationwide cohort study. BMJ. 2016;353:i3189.

32. Laugesen EK, Staerk L, Carlson N, Kamper AL, Olesen JB, Torp-Pedersen C, et al. Non-vitamin K antagonist oral anticoagulants vs. vitamin-K antagonists in patients with atrial fibrillation and chronic kidney disease: a nationwide cohort study. Thromb J. 2019;17:21.

33. Lee HF, Chan YH, Tu HT, Kuo CT, Yeh YH, Chang SH, et al. The effectiveness and safety of low-dose rivaroxaban in Asians with non-valvular atrial fibrillation. Int J Cardiol. 2018;261:78-83.

34. Lee SR, Choi EK, Kwon S, Han KD, Jung JH, Cha MJ, et al. Effectiveness and Safety of Contemporary Oral Anticoagulants Among Asians With Nonvalvular Atrial Fibrillation. Stroke. 2019;50(8):2245-9.

35. Li X, Deitelzweig S, Keshishian A, Hamilton M, Horblyuk R, Gupta K, et al. Effectiveness and safety of apixaban versus warfarin in non-valvular atrial fibrillation patients in “real-world” clinical practice. Thrombosis and Haemostasis. 2017;117(06):1072-82.

36. Lin J, Trocio J, Gupta K, Mardekian J, Lingohr-Smith M, Menges B, et al. Major bleeding risk and healthcare economic outcomes of non-valvular atrial fibrillation patients newly-initiated with oral anticoagulant therapy in the real-world setting. J Med Econ. 2017;20(9):952-61.

37. Lip GY, Keshishian A, Kamble S, Pan X, Mardekian J, Horblyuk R, et al. Real-world comparison of major bleeding risk among non-valvular atrial fibrillation patients initiated on apixaban, dabigatran, rivaroxaban, or warfarin. A propensity score matched analysis. Thromb Haemost. 2016;116(5):975-86.

38. Lip GYH, Keshishian A, Li X, Hamilton M, Masseria C, Gupta K, et al. Effectiveness and Safety of Oral Anticoagulants Among Nonvalvular Atrial Fibrillation Patients. Stroke. 2018;49(12):2933-44.

39. Loo SY, Coulombe J, Dell'Aniello S, Brophy JM, Suissa S, Renoux C. Comparative effectiveness of novel oral anticoagulants in UK patients with non-valvular atrial fibrillation and chronic kidney disease: a matched cohort study. BMJ Open. 2018;8(1):e019638.

40. Mueller S, Groth A, Spitzer SG, Schramm A, Pfaff A, Maywald U. Real-world effectiveness and safety of oral anticoagulation strategies in atrial fibrillation: a cohort study based on a German claims dataset. Pragmat Obs Res. 2018;9:1-10.

41. Nielsen PB, Skjøth F, Søgaard M, Kjældgaard JN, Lip GYH, Larsen TB. Effectiveness and safety of reduced dose non-vitamin K antagonist oral anticoagulants and warfarin in patients with atrial fibrillation: propensity weighted nationwide cohort study. BMJ. 2017;356:j510.

42. Noseworthy PA, Yao X, Abraham NS, Sangaralingham LR, McBane RD, Shah ND. Direct Comparison of Dabigatran, Rivaroxaban, and Apixaban for Effectiveness and Safety in Nonvalvular Atrial Fibrillation. Chest. 2016;150(6):1302-12.

43. Okumura Y, Yokoyama K, Matsumoto N, Tachibana E, Kuronuma K, Oiwa K, et al. Three-Year Clinical Outcomes Associated With Warfarin vs. Direct Oral Anticoagulant Use Among Japanese Patients With Atrial Fibrillation- Findings From the SAKURA AF Registry. Circ J. 2018;82(10):2500-9.

44. Palamaner Subash Shantha G, Bhave PD, Girotra S, Hodgson-Zingman D, Mazur A, Giudici M, et al. Sex-Specific Comparative Effectiveness of Oral Anticoagulants in Elderly Patients With Newly Diagnosed Atrial Fibrillation. Circ Cardiovasc Qual Outcomes. 2017;10(4).

45. Ramagopalan S, Allan V, Saragoni S, Esposti LD, Alessandrini D, Perrone V, et al. Patient characteristics and bleeding events in nonvalvular atrial fibrillation patients treated with apixaban or vitamin K antagonists: real-world evidence from Italian administrative databases. J Comp Eff Res. 2018;7(11):1063-71.

46. Russo-Alvarez G, Martinez KA, Valente M, Bena J, Hu B, Luxenburg J, et al. Thromboembolic and Major Bleeding Events With Rivaroxaban Versus Warfarin Use in a Real-World Setting. Annals of Pharmacotherapy. 2017;52(1):19-25.

47. Siontis KC, Zhang X, Eckard A, Bhave N, Schaubel DE, He K, et al. Outcomes Associated With Apixaban Use in Patients With End-Stage Kidney Disease and Atrial Fibrillation in the United States. Circulation. 2018;138(15):1519-29.

48. Sjogren V, Bystrom B, Renlund H, Svensson PJ, Oldgren J, Norrving B, et al. Non-vitamin K oral anticoagulants are non-inferior for stroke prevention but cause fewer major bleedings than well-managed warfarin: A retrospective register study. PLoS One. 2017;12(7):e0181000.

49. Vinogradova Y, Coupland C, Hill T, Hippisley-Cox J. Risks and benefits of direct oral anticoagulants versus warfarin in a real world setting: cohort study in primary care. BMJ. 2018;362:k2505.

50. Wang CL, Wu VC, Kuo CF, Chu PH, Tseng HJ, Wen MS, et al. Efficacy and Safety of Non-Vitamin K Antagonist Oral Anticoagulants in Atrial Fibrillation Patients With Impaired Liver Function: A Retrospective Cohort Study. J Am Heart Assoc. 2018;7(15):e009263.

51. Weir MR, Berger JS, Ashton V, Laliberte F, Brown K, Lefebvre P, et al. Impact of renal function on ischemic stroke and major bleeding rates in nonvalvular atrial fibrillation patients treated with warfarin or rivaroxaban: a retrospective cohort study using real-world evidence. Curr Med Res Opin. 2017;33(10):1891-900.

52. Weir MR, Haskell L, Berger JS, Ashton V, Laliberte F, Crivera C, et al. Evaluation of clinical outcomes among nonvalvular atrial fibrillation patients treated with rivaroxaban or warfarin, stratified by renal function. Clin Nephrol. 2018;89(5):314-29.

53. Yamashita Y, Uozumi R, Hamatani Y, Esato M, Chun YH, Tsuji H, et al. Current Status and Outcomes of Direct Oral Anticoagulant Use in Real-World Atrial Fibrillation Patients- Fushimi AF Registry. Circ J. 2017;81(9):1278-85.

54. Yao X, Abraham NS, Sangaralingham LR, Bellolio MF, McBane RD, Shah ND, et al. Effectiveness and Safety of Dabigatran, Rivaroxaban, and Apixaban Versus Warfarin in Nonvalvular Atrial Fibrillation. J Am Heart Assoc. 2016;5(6).

55. Yap LB, Eng DT, Sivalingam L, Rusani BI, Umadevan D, Muhammad Z, et al. A Comparison of Dabigatran With Warfarin for Stroke Prevention in Atrial Fibrillation in an Asian Population. Clin Appl Thromb Hemost. 2016;22(8):792-7.
