# Supplementary material for: Major Hemorrhage Risk Associated with Direct Oral Anticoagulants in Non-Valvular Atrial Fibrillation: A Systematic Review and Meta-Analysis
Source: Rev Cardiovasc Med. 2022 Oct 10;23(10):334. doi: 10.31083/j.rcm2310334 (PMC11267317; doi:10.31083/j.rcm2310334)
Supplement: Supplementary file 1 [file 2153-8174-23-10-334-s1.zip › MOOSE_2020_Checklist.docx]

**Major Hemorrhage risk associated with Direct Oral Anticoagulants in Non-Valvular Atrial Fibrillation: A Systematic Review and Meta-analysis**

**Meta-analyses Of Observational Studies in Epidemiology Checklist**

| **Checklist Items** | | **Status** |
| --- | --- | --- |
| Reporting of background should include | | |
|  | Problem definition | COMPLETED |
|  | Hypothesis statement | DEFERRED |
|  | Description of study outcomes | COMPLETED |
|  | Type of exposure or intervention used | COMPLETED |
|  | Type of study designs used | COMPLETED |
|  | Study population | COMPLETED |
| Reporting of search strategy should include | | |
|  | Qualifications of searchers (eg, librarians and investigators) | COMPLETED |
|  | Search strategy, including time period included in the synthesis and keywords | COMPLETED |
|  | Effort to include all available studies, including contact with authors | COMPLETED |
|  | Databases and registries searched | COMPLETED |
|  | Search software used, name and version, including special features used (eg, explosion) | COMPLETED |
|  | Use of hand searching (eg, reference lists of obtained articles) | COMPLETED |
|  | List of citations located and those excluded, including justification | COMPLETED |
|  | Method of addressing articles published in languages other than English | DEFERRED |
|  | Method of handling abstracts and unpublished studies | DEFERRED |
|  | Description of any contact with authors | DEFERRED |
| Reporting of methods should include | | |
|  | Description of relevance or appropriateness of studies assembled for assessing the hypothesis to be tested | COMPLETED |
|  | Rationale for the selection and coding of data (eg, sound clinical principles or convenience) | COMPLETED |
|  | Documentation of how data were classified and coded (eg, multiple raters, blinding, and interrater reliability) | COMPLETED |
|  | Assessment of confounding (eg, comparability of cases and controls in studies where appropriate) | COMPLETED |
|  | Assessment of study quality, including blinding of quality assessors; stratification or regression on possible predictors of study results | COMPLETED |
|  | Assessment of heterogeneity | COMPLETED |
|  | Description of statistical methods (eg, complete description of fixed or random effects models, justification of whether the chosen models account for predictors of study results, dose-response models, or cumulative meta-analysis) in sufficient detail to be replicated | COMPLETED |
|  | Provision of appropriate tables and graphics | COMPLETED |
| Reporting of results should include | | |
|  | Graphic summarizing individual study estimates and overall estimate | COMPLETED |
|  | Table giving cescriptive information for each study included | COMPLETED |
|  | Results of sensitivity testing (eg, subgroup analysis) | COMPLETED |
|  | Indication of statistical uncertainty of findings | COMPLETED |
| Reporting of discussion should include | | |
|  | Quantitative assessment of bias (eg, publication bias) | COMPLETED |
|  | Justification for exclusion (eg, exclusion of non—English-language citations) | COMPLETED |
|  | Assessment of quality of included studies | COMPLETED |
| Reporting of conclusions should include | | |
|  | Consideration of alternative explanations for observed results | COMPLETED |
|  | Generalization of the conclusions (ie, appropriate for the cata presented and within the domain of the literature review) | COMPLETED |
|  | Guidelines for future research | COMPLETED |
|  | Disclosure of funding source | COMPLETED |
